# Supplementary material for: Population dynamics and biological feasibility of sustainable harvesting as a conservation strategy for tropical and temperate freshwater turtles
Source: PLoS One. 2020 Feb 27;15(2):e0229689. doi: 10.1371/journal.pone.0229689 (PMC7046234; doi:10.1371/journal.pone.0229689)
Supplement: S1 Table — (DOCX) [file pone.0229689.s001.docx]

S1 Table. The life history traits data obtained from literature review. Carapace length (CL) and plastron length (PL) are in mm. Body mass (BM), egg mass (EM), and clutch mass (CM) are in gram. Age is age at sexual maturity for female in year. Other abbreviations are clutch size (CS), clutch frequency (CF), and adult survival rate (AS). Missing values were estimated when possible. Numbers inside parenthesis are range values of each life history traits. Asteriks indicate life history traits data from captive breeding.

| **No** | **Species** | **Family** | **CL** | **PL** | **BM** | **CS** | **EM** |
| --- | --- | --- | --- | --- | --- | --- | --- |
| 1 | *Acanthochelys pallidipectoris* | Chelidae | (130 - 140) |  | (325 - 400) | (2 - 5) | 10.34 |
| 2 | *Acanthochelys radiolata* | Chelidae | 174  (152 - 195) |  |  | 3.3  (2 - 4) | 9.87  (5.2 - 12.1) |
| 3 | *Acanthochelys spixii* | Chelidae | 139.78 | 118.76 | 330.87 |  |  |
| 4 | *Actinemys marmorata* | Emydidae | 144.8 |  | 448.7 | 5.2  (3 - 7) |  |
| 5 | *Actinemys marmorata* | Emydidae | 144  (132 - 160) |  | 523  (400 - 750) | 4.46  (3 - 6) |  |
| 6 | *Actinemys marmorata* | Emydidae |  | 106 | 282 | 3 | 8.26 |
| 7 | *Actinemys marmorata* | Emydidae | 152  (140 - 164) |  |  | 5.7  (3 - 8) |  |
| 8 | *Actinemys marmorata* | Emydidae | 149.84  (135.7 - 170.1) |  |  |  |  |
| 9 | *Actinemys marmorata* | Emydidae |  |  |  |  |  |
| 10 | *Apalone ferox* | Trionychidae | 339.3  (302 - 381) |  | 3326 | 16.14 | 12.08 |
| 11 | *Apalone mutica* | Trionychidae | 211 |  | 819 | 10.4 | 6.72 |
| 12 | *Apalone spinifera* | Trionychidae |  |  |  |  |  |
| 13 | *Apalone spinifera* | Trionychidae |  |  |  | 10  (3 - 13) |  |
| 14 | *Apalone spinifera* | Trionychidae | 372.5 |  | 4765 | 31.5 | 9.32 |
| 15 | *Batagur affinis* | Geoemydidae | 560 |  |  | 26.9  (7 - 43) |  |
| 16 | *Batagur baska* | Geoemydidae | 488 |  | 17900 | 26 | 65.15 |

| **CM** | **CF** | **Age** | **AS** | **Latitude** | **Longitude** | **Source** |
| --- | --- | --- | --- | --- | --- | --- |
|  |  |  |  | -25.82 | -61.19 | Richard (1991); Horne (1993) in Vinke et al. (1) & in Iverson et al. (2) |
| 32.57 |  |  |  | -22.91 | -43.20 | Mocelin et al. (3) |
|  |  |  | 0.82 | -15.68 | -47.93 | Fraxe Neto et al. (4) |
|  | (1 - 2) | (4 - 8) |  | 34.73 | -120.57 | Germano and Rathbun (5) |
|  |  |  |  | 35.11 | -116.06 | Lovich and Meyer (6) |
| 24.8 |  |  |  | 35.27 | -120.66 | Congdon and Gibbons (7) |
|  | 1.3  (0 - 2) |  |  | 35.38 | 120.45 | Scott et al. (8) |
|  |  |  |  | 43.22 | -123.36 | Germano and Bury (9) |
|  |  |  | 0.88 | 45.63 | -122.00 | Haegen et al. (10) |
| 194.9 | (4 - 6) |  |  | 28.10 | -81.60 | Iverson (11); Iverson et al. (2) |
| 69.9 | 2 |  |  | 39.08 | -95.65 | Fitch and Plummer (12); Plummer, 1977 in Iverson et al. (2) |
|  |  |  | 0.84 | 35.26 | -91.73 | Plummer et al. (13) |
|  | 1 |  |  | 41.00 | -86.00 | Baker et al. (14) |
| 293.6 |  |  |  | 42.00 | -102.00 | J. B. Iverson, unpublished data in Iverson et al. (2) |
|  |  |  |  | 5.62 | 102.79 | Chan and Chen (15); Cox et al. (16) |
| 1694 |  |  |  | 3.13 | 101.70 | Moll and Moll (17) |

| **No** | **Species** | | | **Family** | | **CL** | | | **PL** | | **BM** | **CS** | **EM** |
| --- | --- | --- | --- | --- | --- | --- | --- | --- | --- | --- | --- | --- | --- |
| 17 | *Batagur baska* | | | Geoemydidae | | 610 | | |  | |  | 10.3 |  |
| 18 | *Batagur baska* | | | Geoemydidae | |  | | |  | |  | 31.6  (19 - 48) | 69 |
| 19 | *Batagur borneoensis* | | | Geoemydidae | | 466 | | |  | | 16900 | 11.4 | 70.4 |
| 20 | *Batagur dhongoka* | | | Geoemydidae | | 406 | | |  | |  | 30.35 |  |
| 21 | *Batagur dhongoka* | | | Geoemydidae | | 420 | | |  | | 7270 | 23 | 35.2 |
| 22 | *Batagur kachuga* | | | Geoemydidae | | 300 | | |  | |  | 10.2 |  |
| 23 | *Batagur kachuga* | | | Geoemydidae | | 560 | | |  | | 21100 | 17 | 55.3 |
| 24 | *Carettochelys insculpta* | | | Carettochelydae | | 555.10 | | |  | |  | 22.39 |  |
| 25 | *Carettochelys insculpta* | | | Carettochelydae | | 526  (420 - 650) | | |  | | 14300 | 24.6  (13 - 37) | 48  (39.3 - 66.6) |
| 26 | *Carettochelys insculpta* | | | Carettochelydae | | 477  (404 - 520) | | |  | | 11200 | 23.6  (19 - 29) | 36.4  (33.8 - 40.1) |
| 27 | *Carettochelys insculpta* | | | Carettochelydae | | 457 | | |  | | 10500 | 15  (14 - 16) | 40.2 |
| 28 | *Carettochelys insculpta* | | | Carettochelydae | |  | | |  | |  | 10.4 | 35.2 |
| 29 | *Chelodina canni* | | | Chelidae | | 193 | | |  | | 719 | 10 | 8.6 |
| 30 | *Chelodina colliei* | | | Chelidae | |  | | |  | |  | 7.3  (2 - 15) |  |
| 31 | *Chelodina colliei* | | | Chelidae | | 198.33  (147 - 247) | | |  | |  | 14.67  (13 - 16) |  |
| 32 | *Chelodina colliei* | | | Chelidae | | 125.1  (113 - 134.2) | | |  | | 1132.5  (670 - 1440) | 9  (6 - 12) | 9.9 (6.3 - 11.5) |
| 33 | Chelodina expansa | | | Chelidae | |  | | |  | |  | 12.4  (9 - 17) |  |
| 34 | *Chelodina expansa* | | | Chelidae | | 362 | | |  | | 4744 | 28 | 18.34 |
| 35 | *Chelodina expansa* | | | Chelidae | |  | | |  | |  |  |  |
| 36 | Chelodina longicollis | | | Chelidae | |  | | |  | |  | 14  (6 - 23) |  |
| 37 | *Chelodina longicollis* | | | Chelidae | | 242.73  (162.5 - 228.7) | | |  | |  | 8.4  (5 - 13) |  |
| 38 | *Chelodina longicollis* | | | Chelidae | |  | | |  | |  |  |  |
| 39 | *Chelodina longicollis* | | | Chelidae | |  | | |  | |  |  |  |
| **CM** | | **CF** | **Age** | | **AS** | | **Latitude** | **Longitude** | | **Source** | | | |
|  | |  |  | |  | | 21.76 | 87.60 | | Das (1985) in Premkishore and Chandran (18) | | | |
| 2180.4 | |  |  | |  | | 22.25 | 88.76 | | Moll, 1990b in Moll et al. (19) | | | |
| 798.6 | |  |  | |  | | 6.00 | 101.00 | | E.O. Moll, unpublished data in Iverson et al. (2) | | | |
|  | |  |  | |  | | 25.80 | 83.91 | | Das (1985) in Premkishore and Chandran (18) | | | |
| 809 | |  |  | |  | | 30.90 | 79.12 | | Moll and Moll (17) | | | |
|  | |  |  | |  | | 25.81 | 83.09 | | Das (1985) in Premkishore and Chandran (18) | | | |
| 940 | |  |  | |  | | 30.90 | 79.12 | | Moll and Moll (17) | | | |
|  | |  |  | |  | | -7.31 | 144.25 | | Eisemberg et al. (20) | | | |
| 1131.3  (308 - 1669) | | 2 |  | |  | | -7.65 | 144.29 | | Georges et al. (21) | | | |
| 858  (797.2 - 946.1) | | 3 |  | |  | | -7.65 | 144.29 | | Georges et al. (21) | | | |
| 603  (562.8 - 634.2) | | 2 |  | |  | | -13.57 | 132.58 | | Georges and Kennett (22) | | | |
| 360.9 | | 1.8  (1 - 2) |  | |  | | -14.08 | 131.25 | | Doody et al. (23) | | | |
| 86 | |  |  | |  | | -16.18 | 133.53 | | Kennett et al. (24) | | | |
|  | |  |  | |  | | -31.76 | 115.79 | | Nicholson, 1975 in Kennett (25) | | | |
|  | | (2 - 3) |  | |  | | -31.95 | 115.86 | | Kuchling (26) | | | |
| 89.1 | | (1 - 2) |  | |  | | -32.17 | 115.83 | | Clay (27) | | | |
|  | |  |  | |  | | -27.00 | 152.00 | | Georges (1984) in Kennett (25) | | | |
| 513.5 | |  | 12 | |  | | -35.93 | 144.23 | | Goode and Russell (28); Banks (1983) in Iverson et al. (2); Spencer (29) | | | |
|  | |  |  | | 0.92 | | -36.13 | 146.00 | | Spencer and Thompson (30) | | | |
|  | | (1 - 2) | (10 - 11) | | 0.98 | | -30.50 | 151.65 | | Parmenter (1985) in Kennett (25); Parmenter (1976) in Kennett (31) & in Shine and Iverson (32) | | | |
|  | |  |  | |  | | -35.15 | 150.72 | | Kennett and Georges (33) | | | |
|  | |  |  | | (0.85 - 0.94) | | -35.15 | 150.72 | | Roe et al. (34) | | | |
|  | |  |  | | 0.95 | | -35.16 | 149.16 | | Rees et al. (35) | | | |

| **No** | **Species** | **Family** | **CL** | **PL** | **BM** | **CS** | **EM** |
| --- | --- | --- | --- | --- | --- | --- | --- |
| 40 | *Chelodina longicollis* | Chelidae |  |  |  | 11.3  (9 - 16) | 7.3 |
| 41 | *Chelodina longicollis* | Chelidae |  |  |  | 19  (13 - 24) |  |
| 42 | *Chelodina longicollis* | Chelidae | 233 |  | 1239 | 13.9  (7 - 18) | 7.38 |
| 43 | *Chelodina longicollis* | Chelidae |  |  |  | 10  (6 - 15) |  |
| 44 | Chelodina longicollis | Chelidae |  |  |  | 9.6  (6 - 12) |  |
| 45 | *Chelodina Mccordi** | Chelidae | 191  (150 - 214) |  |  | 12.2  (7 - 16) |  |
| 46 | *Chelodina Mccordi** | Chelidae |  |  |  | 9.9  (7 - 15) |  |
| 47 | *Chelodina oblonga* | Chelidae | 230 |  | 1217 | 10.5 | 16.36 |
| 48 | *Chelodina oblonga* | Chelidae | (140 - 220) |  |  |  |  |
| 49 | *Chelodina oblonga* | Chelidae | 217.7  (206.7 - 228.6) |  |  |  |  |
| 50 | *Chelodina oblonga* | Chelidae | 210.5  (204.4 - 213.5) |  |  | (10 - 15) | 14.9  (10.6 - 20) |
| 51 | Chelodina steindachneri | Chelidae | 154.75  (146 - 161) |  |  | 7.5  (7 - 8) |  |
| 52 | Chelus fimbriata | Chelidae | 275 |  | 5000 | 12 | 28.81 |
| 53 | *Chelydra acutirostris* | Chelydridae | 312 | 245 | 6953 | 27 | 20.42 |
| 54 | *Chelydra acutirostris* | Chelydridae | 350 | 265 | 10752 | 35.2 | 20.35 |
| 55 | *Chelydra serpentina* | Chelydridae | 216 | 169 | 2254 | 15.3  (6 - 21) | 9.9 |
| 56 | *Chelydra serpentina* | Chelydridae | 232 | 181 | 2798 | 20.5  (16 - 31) | 11.42 |
| 57 | *Chelydra serpentina* | Chelydridae |  |  |  |  |  |
| 58 | *Chelydra serpentina* | Chelydridae | 224 | 175 | 2870 | 19.9  (12 - 42) |  |

| **CM** | **CF** | **Age** | **AS** | **Latitude** | **Longitude** | **Source** |
| --- | --- | --- | --- | --- | --- | --- |
| 82.49 |  |  |  | -35.19 | 149.11 | Palmer-Allen et al. (36) |
|  | 1 |  |  | -35.31 | 149.12 | Vestjens (1969) in Kennett (25) |
| 102.6 | 2 | 9 |  | -35.56 | 138.89 | Chessman (1978) in Iverson et al. (2) |
|  |  |  |  | -35.93 | 144.23 | Goode and Russell (1968) in Kennett (25) |
|  | (1 - 2) | 12 |  | -37.85 | 147.58 | Chessman (1978) in Kennett (31) & in (25) |
|  |  |  |  | -10.73 | 123.12 | Maran and Coutard (2003)* in Rhodin et al. (37); Rhodin (38) |
|  |  |  |  | -10.73 | 123.12 | Symanski (2004)* in Rhodin et al. (37) |
| 171.8 |  |  |  | -9.00 | 143.00 | Rhodin and mittermeier (1976) in Iverson et al. (2) |
|  |  | 5.1 | 0.882  (0.834 - 0.93) | -12.00 | 134.00 | Fordham et al. (39) |
|  |  | 7  (5.8 - 8.7) |  | -12.43 | 130.95 | Kennett (31) |
|  | (2 - 4) | 6.2  (5.6 - 6.5) |  | -12.43 | 130.95 | Kennett (25) |
|  | 1 |  |  | -25.00 | 120.00 | Kuchling (26) |
| 345.7 |  |  |  | 6.49 | -67.13 | Mondolfi (40); Hausmann, (1968) in Iverson et al. (2) |
| 551.3 |  |  |  | 8.00 | -77.00 | Medem (1962, 1977) in Iverson et al. (41) |
| 716.3 |  |  |  | 10.50 | -83.50 | D. Moll, pers. comm in Iverson et al. (41) |
| 151.8 |  |  |  | 27.24 | -82.32 | Punzo (1975, pers.comm) in Iverson et al. (41) |
| 234.1 |  |  |  | 31.50 | -91.50 | E. Moll, pers.comm in Iverson et al. (41) |
|  |  |  | 0.91 | 35.23 | -80.84 | Eskew et al. (42) |
|  |  |  |  | 36.00 | -86.50 | White and Murphy (1973) in Iverson et al. (41) |

| **No** | **Species** | | **Family** | **CL** | **PL** | **BM** | **CS** | **EM** |
| --- | --- | --- | --- | --- | --- | --- | --- | --- |
| 59 | | *Chelydra serpentina* | Chelydridae | 261 | 204 | 4295 | 30.4  (10 - 43) |  |
| 60 | | *Chelydra serpentina* | Chelydridae | 224.1 |  | 2856 | 23.6 | 9.63 |
| 61 | | *Chelydra serpentina* | Chelydridae | 281 | 211 | 5300 | 55 | 12.6 |
| 62 | | *Chelydra serpentina* | Chelydridae | 268 | 204 | 4441 | 31.8  (29 - 37) | 11.63 |
| 63 | | *Chelydra serpentina* | Chelydridae | 260 | 203 | 3963 | 30  (10 - 47) | 11.71 |
| 64 | | *Chelydra serpentina* | Chelydridae |  |  |  |  |  |
| 65 | | *Chelydra serpentina* | Chelydridae | 246 | 192 | 3336 | 35  (18 - 48) |  |
| 66 | | *Chelydra serpentina* | Chelydridae | 261 | 202 | 4220 | 32.3  (15 - 52) | 9.69 |
| 67 | | *Chelydra serpentina* | Chelydridae | 295 | 208 | 5857 | 30  (15 - 50) | 10.3 |
| 68 | | *Chelydra serpentina* | Chelydridae |  |  |  | 20  (8 - 25) |  |
| 69 | | *Chelydra serpentina* | Chelydridae | 281 | 220 | 5046 | 40  (34 - 51) | 12.27 |
| 70 | | *Chelydra serpentina* | Chelydridae |  |  |  |  |  |
| 71 | | *Chelydra serpentina* | Chelydridae | 237 | 185 | 2995 | 20.8  (12 - 30) | 12.54 |
| 72 | | *Chelydra serpentina* | Chelydridae | 325 | 256 | 7878 | 46.8  (20 - 73) | 11.4 |
| 73 | | *Chelydra serpentina* | Chelydridae | 238 | 186 | 3033 | 23  (16 - 37) |  |
| 74 | | *Chelydra serpentina* | Chelydridae | 319 | 247 | 7364 | 49  (31 - 87) |  |
| 75 | | *Chelydra serpentina* | Chelydridae | 268 |  |  | 41.5 | 10.74 |
| 76 | | *Chelydra serpentina* | Chelydridae | 296 | 231 | 5918 | 35.5  (26 - 40) |  |
| 77 | | *Chelydra serpentina* | Chelydridae |  |  |  |  |  |
| 78 | | *Chelydra serpentina* | Chelydridae | 259 | 196 | 4370 | 30.9  (16 - 59) | 11.1 |
| 79 | | *Chelydra serpentina* | Chelydridae | 283 |  |  | 33 | 12.79 |

| **CM** | **CF** | **Age** | **AS** | **Latitude** | **Longitude** | **Source** |
| --- | --- | --- | --- | --- | --- | --- |
|  |  |  |  | 36.00 | -81.00 | Brown (1992) in Iverson et al. (41) |
| 227.2 | 1 | 12 |  | 36.10 | -80.26 | Congdon and Gibbons (7) |
| 693 |  |  |  | 36.65 | -75.93 | Mithcell and Pague (1991) in Iverson et al. (41) |
| 385.6 |  |  |  | 37.00 | -93.00 | Thomas and D. Moll, pers.comm in Iverson et al. (41) |
| 304 |  |  |  | 38.50 | -77.00 | Wilgenbusch, pers.comm in Iverson et al. (41) |
|  |  |  | 0.97 | 39.02 | -79.47 | Flaherty et al. (43) |
|  |  |  |  | 39.50 | -88.50 | E.Moll pers.comm in Iverson et al. (41) |
| 309.8 |  |  |  | 40.50 | -74.50 | Hotaling (1990) in Iverson et al. (41) |
| 310 |  |  |  | 40.50 | -76.50 | Ernst, pers.comm in Iverson et al. (41) |
|  | 1 |  |  | 41.00 | -86.00 | Baker et al. (14) |
|  |  |  |  | 42.00 | -90.00 | Janzen pers.comm in Iverson et al. (41) |
|  | 0.85 | 12 | 0.93 | 42.46 | -83.96 | Congdon et al. (44) |
| 260.8 |  |  |  | 42.50 | -76.50 | Pell (1941) in Iverson et al. (41) |
| 526.9 |  |  |  | 42.50 | -102.50 | Iverson et al. (1997) |
|  |  |  |  | 42.50 | -88.00 | E.Moll pers.comm in Iverson et al. (41) |
|  |  |  |  | 43.00 | -101.50 | Hammer (1969) in Iverson et al. (41); Iverson (45) |
| 445.67 |  |  |  | 43.28 | -79.88 | Brown et al. (46) |
|  |  |  |  | 43.50 | -89.50 | E.Moll pers.comm in Iverson et al. (41) |
|  |  |  | 0.944  (0.857 - 1) | 43.73 | -91.24 | Paisley et al. (47) |
| 308 |  |  |  | 44.50 | -75.00 | Petrokas and Alexander (1980) in Iverson et al. (41) |
| 422.2 |  |  |  | 45.58 | -78.50 | Brown et al. (46) |

| **No** | **Species** | **Family** | **CL** | **PL** | **BM** | **CS** | **EM** |
| --- | --- | --- | --- | --- | --- | --- | --- |
| 80 | *Chelydra serpentina* | Chelydridae | 344 |  |  |  |  |
| 81 | *Chelydra serpentina* | Chelydridae | 284 |  | 5170 | 34 | 11.6 |
| 82 | *Chelydra serpentina* | Chelydridae | 258  (201 - 297) | 201.3  (159 - 235) | 3875 | 27.9  (12 - 41) | 17 |
| 83 | *Chitra indica* | Trionychidae | 1110 |  | 108000 | 102 | 20 |
| 84 | *Chitra indica* | Trionychidae | 644.4  (560 - 795) |  | 30166.36  (14500 - 57000) | 118  (65 - 187) | 10.4  (10 - 18) |
| 85 | *Chrysemys picta* | Emydidae |  | 164  (150 - 174) |  | 9.9  (6 - 14) |  |
| 86 | *Chrysemys picta* | Emydidae | 141.8 |  | 361 | 5 | 6.17 |
| 87 | *Chrysemys picta* | Emydidae |  | 183.3  (177 - 192) |  |  |  |
| 88 | *Chrysemys picta* | Emydidae |  | 182.7  (165 - 202) |  |  |  |
| 89 | *Chrysemys picta* | Emydidae | 132.7 |  | 289 | 4.2 | 4.69 |
| 90 | *Chrysemys picta* | Emydidae | (124 - 150) |  |  |  |  |
| 91 | *Chrysemys picta* | Emydidae |  |  |  | 6  (4 - 14) |  |
| 92 | *Chrysemys picta* | Emydidae |  |  |  | 15.3  (12 - 19) |  |
| 93 | *Chrysemys picta* | Emydidae | 168.5  (148 - 187) |  | 603.1  (400 - 780) | 9  (3 - 14) | 6.66  (5.24 - 8.23) |
| 94 | *Chrysemys picta* | Emydidae | 155.9  (141 - 179) |  | 515.7  (340 - 750) | 8.3  (3 - 12) | 6.12  (4.17 - 7.8) |
| 95 | *Chrysemys picta* | Emydidae | 168.9  (147 - 191) |  | 646.5 (390 - 920) | 11.8  (5 - 18) | 5.4  (3.5 - 7.51) |
| 96 | *Chrysemys picta* | Emydidae | 181.8  (155 - 211) | 174.7  (148 - 203) | 864.9  (520 - 1270) | 13.9  (6 - 21) | 5.9  (3.28 - 9.03) |
| 97 | *Chrysemys picta* | Emydidae |  | (130 - 180) |  |  |  |
| 98 | *Chrysemys picta* | Emydidae |  | 156  (135 - 176) |  | 10.5  (6 - 16) |  |

| **CM** | **CF** | **Age** | **AS** | **Latitude** | **Longitude** | **Source** |
| --- | --- | --- | --- | --- | --- | --- |
|  |  |  | 0.97 | 45.58 | -78.50 | Galbraith and Brooks (48); Galbraith et al. (1989) in Shine and Iverson (32) |
| 394.4 |  |  |  | 45.80 | -78.70 | Obbard (1983) ; Galbraith (1986); Brooks et al. (1986) in Iverson et al. (2) |
| 474.3 | 1 | 12 |  | 48.47 | -84.00 | Congdon et al. (49) |
| 2140 | (3 - 4) |  |  | 23.70 | 90.35 | Wirot (1979) in in Iverson et al. (2) |
| 1227.2 |  |  |  | 30.90 | 79.12 | Rashid and Swingland (50); Bhaduria et. al. (1990) in Das and Singh (51) |
|  |  |  |  | 33.77 | -106.90 | Morjan (52) |
| 30.9 |  |  |  | 33.95 | -83.38 | Congdon and Gibbons (7) |
|  |  | (8 - 9) |  | 37.17 | -107.83 | Cooley et al. (53) |
|  |  | 7 |  | 37.33 | -107.87 | Cooley et al. (53) |
| 19.5 | 2 | 9 | 0.96 | 37.53 | -77.47 | Mitchell (54) & Mitchell (55) |
|  |  | 7 | 0.92 | 40.80 | -73.33 | Zweifel (1989) in Shine and Iverson (32) |
|  | (1 - 3) |  |  | 41.00 | -86.00 | Baker et al. (14) |
|  |  |  |  | 41.00 | -102.00 | Costanzo et al. (56) |
| 62.5  (31.5 - 95.1) |  |  |  | 41.05 | -100.75 | Rowe (57) |
| 50.7  (16.1 - 78.6) |  |  |  | 41.56 | -100.48 | Rowe (57) |
| 65.1  (23.7 - 104.8) |  |  |  | 41.57 | -101.70 | Rowe (57) |
| 82.01  (19.7 - 153.5) | 2.78 | 6 | 0.92 | 41.73 | -102.34 | Iverson and Smith (58); Spencer and Janzen (59) |
|  |  | 5 | 0.83 | 41.95 | -90.12 | Spencer and Janzen (59) |
|  |  |  |  | 41.95 | -90.12 | Morjan (52) |

| **No** | **Species** | **Family** | **CL** | **PL** | **BM** | **CS** | **EM** |
| --- | --- | --- | --- | --- | --- | --- | --- |
| 99 | *Chrysemys picta* | Emydidae | 173.3  (153 - 195) |  | 727.7  (340 - 1060) | 13.8  (7 - 22) | 5.77  (3.46 - 82.3) |
| 100 | *Chrysemys picta* | Emydidae | 140 | 130.7 | 395 | 7.55 | 4.14 |
| 101 | *Chrysemys picta* | Emydidae | 148.6  (128 - 173) | 140.1  (120 - 165) | 409  (280 - 630) | 6.2  (3 - 10) | 6.28  (4.5 - 8.1) |
| 102 | *Chrysemys picta* | Emydidae |  | 178.1 (163 - 207) |  | 15.8  (8 - 21) |  |
| 103 | *Chrysemys picta* | Emydidae |  | 180.1  (162 - 206) |  | 13.4  (10 - 17) |  |
| 104 | *Claudius angustatus* | Staurotypidae | 104 |  | 200 | 4 | 3.5 |
| 105 | *Claudius angustatus* | Staurotypidae | 109.1 |  |  | 2.4  (1 - 6) | 6  (4.3 - 7.9) |
| 106 | *Clemmys guttata* | Emydidae | 103.7 | 91.4 | 193 | 2.7 | 6.8 |
| 107 | *Clemmys guttata* | Emydidae |  | 89.1  (75 - 99) |  | 3.2  (2 - 5) |  |
| 108 | *Clemmys guttata* | Emydidae |  | 89.2 | 165.4 | 3.58 | 5.8 |
| 109 | *Clemmys guttata* | Emydidae | 118.7 | 103.4 | 257.3 | 5.3 | 5.9 |
| 110 | *Cuora amboinensis* | Geoemydidae | 175 |  | 1000 | 1 | 19.5 |
| 111 | *Cuora flavomarginata* | Geoemydidae | 152 |  | 499 | 2 | 13.8 |
| 112 | *Cuora flavomarginata* | Geoemydidae | 155.1  (138 - 174.8) | 148.5  (128.2 - 168.5) | 593.1  (334 - 805) | 1.6  (1 - 3) | 21.5  (12 - 26.7) |
| 113 | *Cuora mccordi* | Geoemydidae | 137.1 |  | 375 | 1.5 | 13.5 |
| 114 | *Cuora mouhotii* | Geoemydidae | 170  (156 - 185) |  | 610.6  (495 - 700) | 3.9  (2 - 5) | 18.1  (11.3 - 23) |
| 115 | *Cuora mouhotii* | Geoemydidae | 158  (103 - 184) |  | 505.6  (200 - 800) | 2.4  (1- 4) | 22.5  (17.2 - 28.7) |
| 116 | *Cuora trifasciata* | Geoemydidae | 214 |  | 1241 | 6 | 14.7 |
| 117 | *Cyclemys dentata* | Geoemydidae | 200 |  | 1250 | 3 | 29.7 |

| **CM** | **CF** | **Age** | **AS** | **Latitude** | **Longitude** | **Source** |
| --- | --- | --- | --- | --- | --- | --- |
| 74  (30.5 - 141.5) |  |  |  | 42.00 | -101.73 | Rowe (57) |
| 31.3 | 0.8 | 8 | 0.76 | 42.47 | -84.00 | Tinkle et al. (60); Wilbur (1975) in Shine and Iverson (32); Congdon and Tinkle (61) |
| 38.86  (14.2 - 64) |  |  |  | 45.60 | -85.58 | Rowe et al. (62) |
|  |  | (7 - 8) |  | 46.75 | -116.93 | Lindeman (63) |
|  |  | (9 - 10) |  | 47.45 | -117.57 | Lindeman (63) |
| 14 |  |  |  | 18.00 | -95.00 | Pauler (1981) in Iverson et al. (2) |
| 14.4  (5.2 - 27.2) | (1 - 2) |  |  | 18.64 | -95.63 | Flores-Villela and Zug (64) |
| 18.4 |  |  |  | 33.23 | -80.36 | Litzgus and Mousseau (65) |
|  |  | 8 |  | 40.00 | -77.50 | Ernst and Zug (66) |
| 20.8 | 1 |  |  | 40.04 | -76.25 | Ernst (1970, 1975) in Iverson et al. (2) & in Shine and Iverson (1995) & in Iverson (45) |
| 31.27 | 0.7 | 12 | 0.97 | 45.00 | -80.00 | Litzgus and Brooks (67); Litzgus (68); Litzgus and Mousseau (2003) in (65) |
| 19.5 |  |  |  | 4.00 | 101.70 | Moll and Moll (17) |
| 27.6 |  |  |  | 24.00 | 121.00 | Fukada (1965) in Iverson et al. (2) |
| 34.4 | 1.9  (1 - 2) | 13 |  | 24.92 | 121.62 | Chen and Lue (69) |
| 20.25 |  |  |  | 24.00 | 108.00 | J. B. Iverson, unpublished data in Iverson et al. (2) |
| 70.59 |  |  |  | 18.73 | 109.87 | Ji-Chao et al. (70) |
| 54 |  |  |  | 18.86 | 110.02 | Ji-Chao et al. (70) |
| 88.3 |  |  |  | 18.85 | 108.64 | McCord and Iverson (1992) in Iverson et al. (2) |
| 89.1 |  |  |  | 4.00 | 101.70 | Moll and Moll (17) |

| **No** | **Species** | **Family** | **CL** | **PL** | **BM** | **CS** | **EM** |
| --- | --- | --- | --- | --- | --- | --- | --- |
| 118 | *Cyclemys dentata* | Geoemydidae | 260 |  |  | 2.4 |  |
| 119 | *Cycloderma frenatum* | Trionychidae | 560 |  | 14591 | 18 | 17.95 |
| 120 | *Cycloderma frenatum* | Trionychidae | 560 |  |  | (17 - 25) |  |
| 121 | *Deirochelys reticularia* | Emydidae | 194.8 | 176.3  (147 - 200) | 1040 | 9.5 | 10.7 |
| 122 | *Deirochelys reticularia* | Emydidae |  | 170.75  (151 - 209) | (648 - 1396) | 9.8  (5 - 17) | 10  (8.4 - 11.3) |
| 123 | *Deirochelys reticularia* | Emydidae | 176.8 |  | 781 | 8 | 9.06 |
| 124 | *Dermatemys mawii* | Dematemydidae | 466  (420 - 502) |  |  | 14.8  (10 - 24) | 49.4  (37 - 72) |
| 125 | *Dermatemys mawii* | Dermatemydidae | 470 |  | 9320 | 10 | 40.8 |
| 126 | *Elseya albagula* | Chelidae | 309.29 |  | 5700  (5200 - 6500) | 12.5  (10 - 16) | 32.5  (28.2 - 36.7) |
| 127 | *Elseya branderhorstii* | Chelidae | 413  (298 - 481) | 337  (250 - 390) |  | 23.5  (15 - 32) | 9 |
| 128 | *Elseya dentate* | Chelidae | 276.5  (264.1 - 284.4) |  |  | (8 - 12) | 27.3  (19.9 - 34.8) |
| 129 | *Elseya dentate* | Chelidae | 343 |  | 4295 | 5 | 15.7 |
| 130 | *Elseya lavarackorum* | Chelidae | 302.1  (203.1 - 352) |  | 2904.8 | (6 - 9) | 25  (18.5 - 28.9) |
| 131 | *Elseya novaeguineae* | Chelidae | 250  (230 - 280) | 199  (187 - 210) |  | 12  (9 - 14) |  |
| 132 | *Elusor macrurus* | Chelidae | 340 |  |  | 14.9  (2 - 22) |  |
| 133 | *Emydoidea blandingii* | Emydidae |  |  |  | 8  (7 - 9) |  |
| 134 | *Emydoidea blandingii* | Emydidae | 209.2  (177 - 235) | 203.4  (173 - 228) | 1336.4  (740 - 1820) | 14.9  (8 - 22) | 11.8  (8.9 - 14.1) |
| 135 | *Emydoidea blandingii* | Emydidae | 208.2  (175.3 - 231.1) |  |  | 15  (3 - 24) | 12.6  (8.6 - 15.9) |
| 136 | *Emydoidea blandingii* | Emydidae |  |  |  |  |  |
| 137 | *Emydoidea blandingii* | Emydidae | 186.9  (161 - 215) |  | 1022.6  (745 - 1432) | 10  (2 - 19) | 12  (5.4 - 14.9) |

| **CM** | **CF** | **Age** | **AS** | **Latitude** | **Longitude** | **Source** |
| --- | --- | --- | --- | --- | --- | --- |
|  |  |  |  | 21.00 | 78.00 | Das (1985) in Premkishore and Chandran (18) |
| 323.1 |  |  |  | -12.00 | 35.00 | Loveridge and Williams (1957) in Iverson et al. (2) |
|  |  |  |  | -14.70 | 35.40 | Broadley and Sachsse (71) |
| 101.7 | 3  (2 - 4) |  |  | 29.68 | -82.37 | Jackson (72) |
| 98 |  | 5 | 0.86 | 33.55 | -81.64 | Buhlmann et al. (73) |
| 72.5 | 3 | 5 |  | 33.55 | -81.64 | Gibbons (74); Congdon et al. (1983a) in Iverson et al. (2) |
| 514.1  (294 - 690) | 2.6  (1 - 4) |  |  | 17.53 | -91.64 | Vogt unpublished data in Vogt et al. (75) |
| 408 |  |  |  | 18.00 | -96.00 | Holman (1963) in Iverson et al. (2) |
| 406.25 |  |  |  | -24.77 | 152.42 | Eiby and Booth (76); Thomson et al. (77) |
| 206.5 |  |  |  | -8.28 | 141.90 | Georges et al. (78) |
| 273 | (1 - 2) | 13.5  (11.4 - 15.4) |  | -13.82 | 131.20 | Kennett (31); Kennett (25) |
| 78.5 |  |  |  | -23.50 | 143.00 | Legler and Cann (1980) in Iverson et al. (2) |
| 187.5 |  |  |  | -18.70 | 138.49 | Freeman et al. (79) |
|  |  |  |  | -8.00 | 141.00 | Georges et al. (78) |
|  |  |  |  | -26.00 | 152.00 | Micheli-Campbell et al. (80); Berry and Shine (81) |
|  | 1 |  |  | 41.00 | -86.00 | Baker et al. (14) |
| 168.4  (92.4 - 235.9) |  |  |  | 42.00 | -101.00 | Rowe (82) |
| 189 |  |  | 0.59 | 42.00 | -101.00 | Ruane et al. (83) |
|  |  |  | 0.96 | 42.46 | -83.96 | Congdon et al. (84) |
| 120 | 0.8  (0 - 1) | 17.77  (14 - 21) |  | 42.47 | -84.00 | Congdon et al. (85) |

| **No** | **Species** | **Family** | **CL** | **PL** | **BM** | **CS** | **EM** |
| --- | --- | --- | --- | --- | --- | --- | --- |
| 138 | *Emydoidea blandingii* | Emydidae | 194.8 |  | 988 | 10.2 | 12.2 |
| 139 | *Emydoidea blandingii* | Emydidae |  |  |  | 10.6  (4 - 15) |  |
| 140 | *Emydura macquarii* | Chelidae | 198.6 | 165.9 | 1071 |  |  |
| 141 | *Emydura macquarii* | Chelidae | 223.8 | 186.1 | 1494.5 |  |  |
| 142 | *Emydura macquarii* | Chelidae | 194.6 | 164.8 | 1283.5 |  |  |
| 143 | *Emydura macquarii* | Chelidae | 196.5 | 165.3 | 1217.2 |  |  |
| 144 | *Emydura macquarii* | Chelidae | 182 |  | 679.3 | 6.18 | 7.44 |
| 145 | *Emydura macquarii* | Chelidae | 223.3  (189 - 247) | 184.2  (153 - 215) | 1125 (620 - 1630) | 16.1 |  |
| 146 | *Emydura macquarii* | Chelidae |  |  |  |  |  |
| 147 | *Emydura macquarrii* | Chelidae | 272 |  | 2187 | 16.4 | 9.75 |
| 148 | *Emydura macquarrii* | Chelidae | 257 |  | 1740 | 18.2  (10 -38) | 10.42 |
| 149 | *Emydura subglobosa* | Chelidae | 189  (152 - 255) | 152  (121 - 209) |  | 7  (4 -11) | 7.68 |
| 150 | *Emydura subglobosa* | Chelidae | 210 |  | 1030 | 10.25 | 9.74 |
| 151 | *Emydura victoriae** | Chelidae | 300 |  | 922.75  (615 - 1100) | 10  (6 - 18) | 8.7  (4.63 - 11.35) |
| 152 | *Emys orbicularis* | Emydidae | 140.46  (115.3 - 166.6) |  | 480.34  (240.1 - 750.7) |  |  |
| 153 | *Emys orbicularis* | Emydidae | 130.56  (80 - 174) | 120.97  (71 - 147) | 407.81  (84 - 700) | 5 |  |
| 154 | *Emys orbicularis* | Emydidae |  |  |  | 12.2  (6 - 16) |  |
| 155 | *Emys orbicularis* | Emydidae | 179  (160 - 210) |  | 881.3  (647 - 1541) | 13.3  (8 - 22) | 8.1  (6.1 - 9.4) |
| 156 | *Geoclemys hamiltonii* | Geoemydidae | 301.4  (207 - 405) |  | 3500  (1450 - 6000) | (18 - 30) | 11.5 |
| 157 | *Geoemyda spengleri* | Geoemydidae | 101 |  | 196 | 1 | 8.2 |
| 158 | *Glyptemys insclupta* | Emydidae | 174.69 |  |  | 7.17  (1 - 10) |  |

| **CM** | **CF** | **Age** | **AS** | **Latitude** | **Longitude** | **Source** |
| --- | --- | --- | --- | --- | --- | --- |
| 111.7 | 0.6 | 17 |  | 43.00 | -84.00 | Congdon et al. (86); Congdon and van Loben Sels (1991) in Iverson et al. (2) |
|  | (0 - 1) |  |  | 44.38 | -65.25 | Standing et al. (87) |
|  |  | (8 - 10) | 0.81 | -19.27 | 146.75 | Trembath (88) |
|  |  |  | 0.60 | -19.31 | 146.77 | Trembath (88) |
|  |  |  | 0.52 | -19.32 | 146.84 | Trembath (88) |
|  |  |  | 0.57 | -19.44 | 146.95 | Trembath (88) |
| 45.98 | 3 | (7 - 8) |  | -25.22 | 153.13 | Georges (1982, 1983) in Iverson et al. (2); Kennett (31); Kennett (25) |
|  |  |  |  | -30.50 | 153.03 | Blamires et al. (89) |
|  |  |  | (0.945 - 0.977) | -36.13 | 146.00 | Spencer and Thompson (30) |
| 159.9 | (2 - 3) |  |  | -23.50 | 150.79 | Legler and Cann (1980) & Georges, 1982 in Iverson et al. (2); Kennett (25) |
| 189.6 | (2 - 3) | 8 |  | -35.56 | 138.89 | Chessman (1978), Thompson (1987) & Banks (1983) in Iverson et al. (2); Spencer (29) |
| 53.76 | 3.11 |  |  | -8.00 | 141.00 | Georges et al. (78) |
| 99.84 |  |  |  | -9.51 | 147.22 | Lovich et al. (1983) in Iverson et al. (2) |
| 87 | (2 - 3) | (6 - 7) |  | -15.80 | 128.73 | Gaikhorst et al. (90)*; Ernst and Barbour (91) |
|  |  |  | 0.82 | 38.05 | 28.77 | Ayaz et al. (92) |
|  |  |  |  | 43.63 | 10.34 | Zulfi et al. (93) |
|  | 2 |  |  | 48.38 | 21.78 | Novotny et al. (94) |
| 107.73 |  | 17.1  (8 - 23) |  | 52.27 | 27.55 | Drobenkov (95) |
| 276 | 2 | (6 - 8) |  | 23.10 | 90.35 | Rashid and Swingland (50); Gurley (2003) in Das and Bhupathy (96) |
| 8.2 |  |  |  | 22.00 | 105.00 | Rudloff (1986) in Iverson et al. (2) |
|  |  | 12 |  | 42.00 | -74.03 | Hunsinger (97) |

| **No** | **Species** | **Family** | **CL** | **PL** | **BM** | **CS** | **EM** |
| --- | --- | --- | --- | --- | --- | --- | --- |
| 159 | *Glyptemys insclupta* | Emydidae | 179.86 |  |  | 9.29  (7 - 12) |  |
| 160 | *Glyptemys insclupta* | Emydidae | 172 |  | 711.4 | 7.8  (6 - 9) |  |
| 161 | *Glyptemys insclupta* | Emydidae | 200  (187 - 221) | 180  (177 - 196) | 1131  (980 - 1320) | 9.2  (5 - 14) |  |
| 162 | *Glyptemys insclupta* | Emydidae | 201  (181 - 224) | 193  (177 - 214) |  | 10.03  (5 - 15) |  |
| 163 | *Glyptemys insculpta* | Emydidae | 198.3 |  | 968.2 | 11 | 14.38 |
| 164 | *Glyptemys muhlenbergii* | Emydidae |  |  |  |  |  |
| 165 | *Glyptemys muhlenbergii* | Emydidae | 91.82 |  | 132.1 | 4 | 4.64 |
| 166 | *Graptemys barbouri* | Emydidae | 220 |  | 1256 | 8.5 | 18.9 |
| 167 | *Graptemys barbouri* | Emydidae |  |  |  | 8.5  (4 - 14) |  |
| 168 | *Graptemys ernsti* | Emydidae | 285 |  |  | 7.2 |  |
| 169 | *Graptemys ernsti* | Emydidae |  | 149.3  (68 -231) |  |  |  |
| 170 | *Graptemys flavimaculata* | Emydidae | 190.49 |  |  | 4.7  (3 - 9) |  |
| 171 | *Graptemys geographica* | Emydidae | 226  (201 - 258) |  | 1138  (761 - 1700) | 10.1  (6 - 15) | 11.23 |
| 172 | *Graptemys geographica* | Emydidae |  |  |  | 10.6  (6 - 15) |  |
| 173 | *Graptemys geographica* | Emydidae |  |  |  | 9  (6 - 14) |  |
| 174 | *Graptemys geographica* | Emydidae |  | 203.8  (169 - 228) |  | 11.9  (3 - 21) | 10.34  (7.39 - 11.95) |
| 175 | *Graptemys nigrinoda* | Emydidae | 155 |  | 415.7 | 5.5 | 12.03 |
| 176 | *Graptemys oculifera* | Emydidae | 148 |  | 359.3 | 3 | 10.6 |
| 177 | *Graptemys oculifera* | Emydidae | 164.6  (130 - 215) |  | 627.9  (319 - 1457) | 3.66  (1- 10) | 11.9  (5 - 17.4) |

| **CM** | **CF** | **Age** | **AS** | **Latitude** | **Longitude** | **Source** |
| --- | --- | --- | --- | --- | --- | --- |
|  |  | 14 |  | 41.20 | -74.26 | Hunsinger (97) |
|  |  |  |  | 43.00 | -71.00 | Tuttle and Carroll (98); Tuttle (99) |
|  |  |  |  | 46.00 | -81.00 | Greaves and Litzgus (100) |
|  |  |  |  | 46.82 | -71.22 | Walde et al. (101) |
| 158.2 |  |  |  | 43.76 | -70.55 | Graham and Forsberg (102) |
|  |  |  | 0.893  (0.853 - 0.924) | 35.30 | -81.18 | Pittman et al. (103) |
| 18.56 |  |  |  | 39.58 | -75.64 | Arndt (1977) & M. A. Ewert, unpublished data in Iverson et al. (2) |
| 160.6 |  |  |  | 31.22 | -84.19 | Wahlquist and Folkerts (1973) in Iverson et al. (2) |
|  |  |  |  | 32.00 | -83.00 | Ewert and Jackson (104) |
|  | 4 | 14 |  | 32.70 | -86.70 | Shealy (1976) in Lindeman (105); Shealy (1978) in Lovich et al. (106); Lovich et al. (107) |
|  |  |  |  | 37.01 | -88.27 | Lindeman (108) |
|  | 1.16  (1 - 3) | 10 |  | 30.76 | -88.67 | Horne et al. (109) |
| 113.42 | 2 | 9 |  | 37.78 | -92.87 | Vogt (110); White and Moll (111); M. A. Ewert, unpublished data in Iverson et al. (2) |
|  |  |  |  | 40.41 | -77.98 | Nagle et al. (112) |
|  | (1 - 2) |  |  | 41.00 | -86.00 | Baker et al. (14) |
| 119.98  (81.3 - 176.8) |  |  |  | 42.16 | -80.10 | Ryan and Lindeman (113) |
| 66.17 | (3 - 4) | (8 - 9) |  | 30.79 | -87.93 | Lahanas (1982) in Iverson et al. (2); Blankenship et al. (114) |
| 31.8 |  |  |  | 32.00 | -89.00 | Cagle (1953) in Iverson et al. (2) |
| 43.55 | 1.1  (0.96 - 142) | (10 - 16) |  | 32.00 | -89.00 | Jones (115); Jones and Selman (116) |

| **No** | **Species** | **Family** | **CL** | **PL** | **BM** | **CS** | **EM** |
| --- | --- | --- | --- | --- | --- | --- | --- |
| 178 | *Graptemys ouachitensis* | Emydidae |  | 145.4  (89 - 185) |  |  |  |
| 179 | *Graptemys ouachitensis* | Emydidae | 205  (163 - 242) |  | 1136  (557 - 2300) | 10.5  (8 - 19) | 9.76 |
| 180 | *Graptemys pseudogeographica* | Emydidae | 225 |  | 1477 | 14.1  (8 - 19) | 9.78 |
| 181 | *Graptemys pulchra* | Emydidae | 247 |  | 1519 | 7.2 | 14.18 |
| 182 | *Graptemys pulchra* | Emydidae | 273 |  |  | (4 - 7) |  |
| 183 | *Graptemys versa* | Emydidae | 150.3  (128 - 183) | 133.8  (115 - 163) |  | 5.6  (4 - 9) |  |
| 184 | *Hardella thurjii* | Geoemydidae | 362.1  (230 - 472) |  | 790.25  (575 - 1100) | (12 -16) | 45.2 |
| 185 | *Heosemys spinosa* | Geoemydidae | 186 |  | 950 | 1 | 48.7 |
| 186 | *Hydromedusa maximiliani* | Chelidae | (100 - 200) |  | (120 - 150) | (1 - 3) |  |
| 187 | *Hydromedusa maximiliani* | Chelidae | 142.7 | 107.8 | 253.6 | (1 - 3) |  |
| 188 | *Kinosternon acutum* | Kinosternidae | 120 | 115 |  | 1.6  (1 - 3) | 6.2  (4.4 - 9.1) |
| 189 | *Kinosternon alamosae* | Kinosternidae | 105  (89.2 - 120.3) |  | 145 | 4  (3 - 5) | 3.66 |
| 190 | *Kinosternon angustipons* | Kinosternidae | 112 |  | 201 | 1.5 | 11.94 |
| 191 | *Kinosternon baurii* | Kinosternidae | 98.8  (79 - 118) | 91.1  (69 - 109) | 171.5  (78 - 269.7) | 2.7  (1 - 6) | 5.6  (3.8 - 8) |
| 192 | *Kinosternon baurii* | Kinosternidae | 91.6 | 82.6 | 143 | 2.55 | 4.45 |
| 193 | *Kinosternon creaseri* | Kinosternidae | 116 |  | 207 | 1 | 8.9 |
| 194 | *Kinosternon dunni* | Kinosternidae | 150 |  | 496 | 2 | 15.98 |
| 195 | *Kinosternon flavescens* | Kinosternidae | 134.5 |  | 391 | 4.7 | 6.31 |
| 196 | *Kinosternon flavescens* | Kinosternidae | 106.3  (95 - 117) |  | 223.2  (150.7 - 275.5) | 4.86 | 4.84 |
| 197 | *Kinosternon flavescens* | Kinosternidae | 102.5  (88.3 - 117) | 101.3  (87.1 - 113.4) | 271.3  (185 - 349) | 6.5  (4 - 9) | 4.25  (3.05 - 5.83) |
| 198 | *Kinosternon herrerai* | Kinosternidae | 143.1 |  | 486 | 2 | 6.95 |

| **CM** | **CF** | **Age** | **AS** | **Latitude** | **Longitude** | **Source** |
| --- | --- | --- | --- | --- | --- | --- |
|  |  |  |  | 37.01 | -88.27 | Lindeman (108) |
| 102.5 | 2 | 9 |  | 43.67 | -93.22 | Vogt (1980) in Iverson et al. (2) & in Lindeman (105) |
| 137.9 |  |  |  | 43.67 | -93.22 | Vogt (1980) in Iverson et al. (2) |
| 102.1 | 4 | 14 |  | 32.00 | -86.00 | Shealey (1976) in Iverson et al. (2) & in Iverson (45) |
|  | (6 - 7) |  |  | 32.84 | -87.11 | Lovich et al. (117) |
|  | (1 - 4) |  |  | 30.45 | -99.81 | Lindeman (105) |
| 632.8 |  |  |  | 23.00 | 90.00 | Rashid and Swingland (50) |
| 48.7 |  |  |  | 4.00 | 101.00 | Moll and Moll (17) |
|  |  | 9  (5.7 - 11.9) | 0.92  (0.919 - 0.921) | -24.13 | -47.96 | Martins and Souza (118); Martins and Souza (119); Souza and Martins (120) |
|  |  |  |  | -24.25 | -47.75 | Famelli et al. (121) |
| 9.92 | (2 - 4) | (5 - 8) |  | 17.68 | -92.49 | Iverson and Vogt (122) |
| 14.6 | 2 | 5 |  | 28.00 | -111.00 | Iverson (1989a) in Iverson et al. (2) |
| 17.91 |  |  |  | 8.00 | -9.00 | Legler (1966) in Iverson et al. (2) |
| 15.12 | 1.6  (1 - 3) |  |  | 27.00 | -82.00 | Wilson et al. (123) |
| 11.35 | 5 | 5 |  | 30.00 | -82.00 | Iverson (1979b) in Iverson et al. (2) |
| 8.9 | 3 | 12 |  | 20.00 | -89.00 | Iverson (1988) in Iverson et al. (2) |
| 31.96 |  |  |  | 5.00 | -74.00 | Medem (1962a) in Iverson et al. (2) |
| 29.66 | 2 | 8 |  | 30.00 | -110.00 | Iverson (1989b) in Iverson et al. (2) |
| 23.5 | 1 | 8 |  | 34.00 | -101.00 | Long (124) |
| 27.6 | 0.75 | 11 | 0.95 | 42.00 | -102.00 | Iverson (125) |
| 13.9 |  |  |  | 23.18 | -97.78 | Carr and Mast (1988) in Iverson et al. (2) |

| **No** | **Species** | | | **Family** | | | **CL** | | **PL** | | **BM** | **CS** | **EM** | |
| --- | --- | --- | --- | --- | --- | --- | --- | --- | --- | --- | --- | --- | --- | --- |
| 199 | *Kinosternon hirtipes* | | | Kinosternidae | | | 107.4  (72.3 - 131.1) | | 96.3  (61.25 - 116.8) | | 202.6  (92 - 325) | 3  (1 - 6) | 4.82 | |
| 200 | *Kinosternon integrum* | | | Kinosternidae | | |  | |  | |  |  |  | |
| 201 | *Kinosternon integrum* | | | Kinosternidae | | |  | |  | |  |  |  | |
| 202 | *Kinosternon integrum* | | | Kinosternidae | | | 142.7  (90 - 195) | | 119.5  (73 - 155) | | 281.35  (60.65 - 654.2) | 4  (1 - 8) | 5.14  (3.41 - 6.57) | |
| 203 | *Kinosternon integrum* | | | Kinosternidae | | | 146.9  (131 - 168) | | 141.4  (123 - 161) | |  | 5  (2 -10) | 5.8 | |
| 204 | *Kinosternon leucostomum* | | | Kinosternidae | | | 137 | |  | | 373 | 1 | 9.12 | |
| 205 | *Kinosternon scorpioides* | | | Kinosternidae | | | 115 | |  | | 266 | 3 | 7.03 | |
| 206 | *Kinosternon scorpioides* | | | Kinosternidae | | | 110  (97 - 141) | |  | | 225.55 | 2.2  (1 -4) | 5.47  (4.37 - 6.55) | |
| 207 | *Kinosternon sonoriense* | | | Kinosternidae | | | 130  (106 - 160) | |  | | 340 | 6.7  (2 - 11) |  | |
| 208 | *Kinosternon sonoriense* | | | Kinosternidae | | |  | |  | |  |  |  | |
| 209 | *Kinosternon sonoriense* | | | Kinosternidae | | | 100 | |  | |  | 4 |  | |
| 210 | *Kinosternon sonoriense* | | | Kinosternidae | | | 111  (86.1 - 152.7) | |  | |  |  |  | |
| 211 | *Kinosternon sonoriense* | | | Kinosternidae | | | 135 | |  | | 351 | 5.3 | 4.2 | |
| 212 | *Kinosternon sonoriense* | | | Kinosternidae | | | 124 | |  | | 326 | 4.5 | 5.03 | |
| 213 | *Kinosternon sonoriense* | | | Kinosternidae | | | 138 | |  | | 449 | 5.35 | 5.84 | |
| 214 | *Kinosternon sonoriense* | | | Kinosternidae | | | 112 | |  | | 245 | 3.09 | 5.22 | |
| 215 | *Kinosternon sonoriense* | | | Kinosternidae | | | 126 | |  | |  |  |  | |
| 216 | *Kinosternon sonoriense* | | | Kinosternidae | | | 130 | |  | |  |  |  | |
| 217 | *Kinosternon sonoriense* | | | Kinosternidae | | | 146 | |  | |  |  |  | |
| 218 | *Kinosternon sonoriense* | | | Kinosternidae | | | 142 | |  | |  |  |  | |
| 219 | *Kinosternon sonoriense* | | | Kinosternidae | | | 104 | |  | | 161 | 2.8  (2 - 4) | 4.2 | |
| 220 | *Kinosternon sonoriense* | | | Kinosternidae | | | 125 | |  | |  | 4.96 (1 - 8) |  | |
| 221 | *Kinosternon subrubrum* | | | Kinosternidae | | |  | |  | |  | 3.1 (1 - 6) |  | |
| **CM** | | **CF** | **Age** | | **AS** | **Latitude** | | **Longitude** | | **Source** | | | |  |
| 14.5  (6.72 - 29.94) | | 4 | 7 | |  | 30.00 | | -106.00 | | Iverson et al. (126) | | | |  |
|  | |  |  | | 0.78 | 18.75 | | -100.60 | | Macip-Rios et al. (127) | | | |  |
|  | |  |  | | 0.77 | 18.75 | | -99.68 | | Macip-Rios et al. (127) | | | |  |
| 20.56 | | 2.26 |  | |  | 18.80 | | -99.67 | | Macip-Rios et al. (128) | | | |  |
|  | | (2 - 4) |  | |  | 21.18 | | -102.66 | | Iverson (129) | | | |  |
| 9.12 | |  |  | |  | 9.00 | | -79.00 | | Moll and Legler (1971) in Iverson et al. (2) | | | |  |
| 21.1 | |  |  | |  | 10.00 | | -84.00 | | Castillo Centeno, 1986 & J. B. Iverson, unpublished data in Iverson et al. (2) | | | |  |
| 11.85  (9.13 - 16.1) | | 5 | (9 - 10) | |  | 18.85 | | -89.13 | | Iverson (130) | | | |  |
|  | | 2 | 5 | |  | 31.93 | | -109.38 | | van Loben Sels et al. (131) | | | |  |
|  | |  |  | | 0.95 | 31.94 | | -113.06 | | Riedle et al. (132) | | | |  |
|  | | (1.4 - 1.6) |  | |  | 31.94 | | -113.02 | | Rosen and Lowe (1996) in Lovich et al. (106) | | | |  |
|  | |  |  | | 0.92 | 32.40 | | -109.15 | | Stone (133) | | | |  |
| 22.3 | | 1 | 12 | |  | 33.51 | | -112.48 | | Hulse (1982) in Iverson et al. (2) | | | |  |
| 22.1 | | 4 | 6 | |  | 34.00 | | -111.00 | | Rosen (1987) in Iverson et al. (2) | | | |  |
| 31.3 | | 3 | 6 | |  | 34.00 | | -111.00 | | Rosen (1987) in Iverson et al. (2) | | | |  |
| 16.4 | | 4 | 6 | |  | 34.00 | | -111.00 | | Rosen (1987) in Iverson et al. (2) | | | |  |
|  | |  |  | | 0.86 | 34.00 | | -111.00 | | Rosen (1987) in Shine and Iverson (32) | | | |  |
|  | |  |  | | 0.94 | 34.00 | | -111.00 | | Rosen (1987) in Shine and Iverson (32) | | | |  |
|  | |  |  | | 0.86 | 34.00 | | -111.00 | | Rosen (1987) in Shine and Iverson (32) | | | |  |
|  | |  |  | | 0.83 | 34.00 | | -111.00 | | Rosen (1987) in Shine and Iverson (32) | | | |  |
| 11.8 | | 2 | 8 | |  | 34.56 | | -112.54 | | Hulse (1982) in Iverson et al. (2) | | | |  |
|  | | 2 |  | |  | 34.61 | | -111.83 | | Lovich et al. (106) | | | |  |
|  | | 1.2  (1 - 3) | 4 | | 0.88 | 33.00 | | -81.00 | | Frazer et al. (134); Gibbons (1983) in Shine and Iverson (32) | | | |  |

| **No** | **Species** | | | **Family** | | **CL** | | **PL** | | | **BM** | **CS** | **EM** |
| --- | --- | --- | --- | --- | --- | --- | --- | --- | --- | --- | --- | --- | --- |
| 222 | *Kinosternon subrubrum* | | | Kinosternidae | |  | | 87.49 | | |  | 3.4 |  |
| 223 | *Kinosternon subrubrum* | | | Kinosternidae | |  | |  | | |  |  |  |
| 224 | *Kinosternon subrubum* | | | Kinosternidae | | 91.4 | | 86 | | | 140 | 3.4 | 3.93 |
| 225 | *Kinosternon subrubum* | | | Kinosternidae | | 96.3 | |  | | | 164.7 | 3.1 | 4.25 |
| 226 | *Leucocephalon yuwonoi* | | | Geoemydidae | | 191.33  (184 - 200) | |  | | |  | 1.33  (1 - 2) |  |
| 227 | *Lissemys punctata* | | | Trionychidae | | 205 | |  | | | 1149 | 5.83 | 11.35 |
| 228 | *Lissemys punctata* | | | Trionychoidea | | 182 | |  | | | 945 | 5.95 | 13.21  (8.8 - 18.9) |
| 229 | *Lissemys punctata* | | | Trionychoidea | |  | |  | | |  | 6.14 |  |
| 230 | *Lissemys punctata* | | | Trionychoidea | |  | |  | | |  | (10 - 12) |  |
| 231 | *Lissemys punctata* | | | Trionychoidea | |  | |  | | |  | (8 - 12) |  |
| 232 | *Lissemys punctata* | | | Trionychoidea | | 258.1  (244 -290) | |  | | | 1851.18  (1400 - 3000) | (7 - 14) | 14.75 |
| 233 | *Macrochelys temminckii* | | | Chelydridae | | 400 | |  | | | 11700 | 24.5 | 29.06 |
| 234 | *Macrochelys temminckii* | | | Chelydridae | | 366  (301 - 465) | |  | | | 13400  (7000 - 23500) |  |  |
| 235 | *Malaclemys terrapin* | | | Emydidae | | 181  (118 - 201) | |  | | | 1008.6  (272 - 1458) |  |  |
| 236 | *Malaclemys terrapin* | | | Emydidae | |  | |  | | | 886 | 6.7  (5 - 10) | 12.48  (11.2 - 13.2) |
| 237 | *Malaclemys terrapin* | | | Emydidae | |  | |  | | |  | 10.9  (3 - 18) |  |
| 238 | *Malaclemys terrapin* | | | Emydidae | |  | |  | | |  |  |  |
| 239 | *Malayemys macrocephala* | | | Geoemydidae | | 148.6  (114.4 - 187) | | 132.28 | | |  | 5.64 |  |
| 240 | *Mauremys annamensis* | | | Geoemydidae | | 212  (189 - 285) | | 184  (155 - 257) | | | 1717  (1302 - 2126) | 5.5 | 15.5 |
| 241 | *Mauremys japonica* | | | Geoemydidae | | 150 | |  | | | 494.2 | 6 | 8.33 |
| 242 | *Mauremys leprosa* | | | Geoemydidae | | 145.8 | |  | | |  |  |  |
| 243 | *Mauremys leprosa* | | | Geoemydidae | | 168.8 | |  | | | 608 | 5.8 | 13.2 |
| 244 | *Mauremys leprosa* | | | Geoemydidae | | 179.2  (137.3 - 218) | |  | | |  | 6.47  (1 - 13) |  |
| **CM** | | **CF** | **Age** | | **AS** | | **Latitude** | | **Longitude** | **Source** | | | |
|  | | 1.32  (1 - 3) |  | |  | | 33.00 | | -81.00 | Wilkinson and Gibbons (135) | | | |
|  | |  |  | | 0.57 | | 35.23 | | -80.84 | Eskew et al. (42) | | | |
| 13.42 | |  |  | |  | | 33.00 | | -81.00 | Congdon and Gibbons (1985) in Iverson et al. (2) | | | |
| 13.17 | | 3 | 6 | |  | | 34.58 | | -93.13 | Iverson (1979a) in Iverson et al. (2) | | | |
|  | |  | (7 - 10) | |  | | 0.52 | | 121.00 | Riyanto (136); Innis (2003) in Hagen et al. (137) | | | |
| 66.17 | |  |  | |  | | 13.00 | | 78.00 | Deraniyagala (1939) & Das (1991) in Iverson et al. (2) | | | |
| 78.60 | |  |  | |  | | 13.09 | | 80.27 | Premkishore and Chandran (18) | | | |
|  | |  |  | |  | | 21.00 | | 78.00 | Das (1985) in Premkishore and Chandran (18) | | | |
|  | |  |  | |  | | 21.00 | | 78.00 | Smith (1931) in Iverson et al. (2) | | | |
|  | |  |  | |  | | 21.00 | | 78.00 | Yadav and Prasad (1982) in Iverson et al. (2) | | | |
|  | |  |  | |  | | 23.00 | | 90.00 | Rashid and Swingland (50) | | | |
| 712.1 | |  | (11 - 13) | |  | | 31.63 | | -91.83 | Dobie (1971) in Iverson et al. (2)) | | | |
|  | |  |  | | 0.88 | | 35.17 | | -92.30 | Howey and Dinkelacker (138) | | | |
|  | |  | (4 - 5) | |  | | 25.27 | | -81.15 | Hart and McIvor (139) | | | |
| 83.62 | |  |  | |  | | 28.52 | | -80.67 | Seigel (1980, 1984) & Burger (1977) in Iverson et al. (2) | | | |
|  | | 2 |  | |  | | 40.45 | | -74.00 | Feinberg and Burke (140) | | | |
|  | |  |  | | (0.94 - 0.959) | | 41.70 | | -71.50 | Mitro (141) | | | |
|  | |  |  | |  | | 14.37 | | 100.49 | Brophy (142); Pewphong et al. (143) | | | |
| 85.25 | | (1 - 4) | (5 - 7) | |  | | 15.91 | | 107.77 | McCormack et al. (144) | | | |
| 49.98 | |  |  | |  | | 34.00 | | 136.06 | Fukada (1965), Fukada and Ishihara (1974) in Iverson et al. (2) | | | |
|  | |  |  | | 0.53 | | 41.82 | | 1.47 | Bertolero and Oro (145) | | | |
| 76.6 | |  | 8 | | 0.79 | | 37.00 | | -6.50 | Perez et al. (1979) & Andreu and Villamor (1989) in Iverson et al. (2) & in Shine and Iverson (32) | | | |
|  | |  |  | |  | | 37.00 | | -6.50 | Keller (146) | | | |

| **No** | **Species** | | | | | **Family** | | | | **CL** | | | | | **PL** | | | | **BM** | | **CS** | | **EM** |
| --- | --- | --- | --- | --- | --- | --- | --- | --- | --- | --- | --- | --- | --- | --- | --- | --- | --- | --- | --- | --- | --- | --- | --- |
| 245 | *Mauremys mutica* | | | | | Geoemydidae | | | | 130 | | | | |  | | | | 319.5 | |  | | 12.7 |
| 246 | *Mauremys mutica** | | | | | Geoemydidae | | | | 179.5 | | | | |  | | | |  | | 4.05  (1 - 8) | |  |
| 247 | *Mauremys nigricans** | | | | | Geoemydidae | | | | 184.67  (175 - 193) | | | | |  | | | | 911.67  (750 - 1065) | | 5.16  (1 -13) | |  |
| 248 | *Mauremys nigricans** | | | | | Geoemydidae | | | | 195  (187 - 200) | | | | |  | | | | 955  (828 - 1091) | | 4.4 | | 11.9  (10 - 13.6) |
| 249 | *Mauremys reevesii* | | | | | Geoemydidae | | | | 195 | | | | |  | | | | 858 | | 6.7 | | 11.2 |
| 250 | *Mauremys sinensis* | | | | | Geoemydidae | | | | 221.7  (198.9 - 248.6) | | | | | 210.1  (188 - 238.5) | | | | 1444  (1144 - 1750) | | 12.6  (7 - 17) | | 8  (6.2 - 10) |
| 251 | *Melanochelys tricarinata* | | | | | Geoemydidae | | | | 163 | | | | |  | | | |  | | (1 - 3) | |  |
| 252 | *Melanochelys trijuga* | | | | | Geoemydidae | | | | 223 | | | | |  | | | | 1316 | | 6.09 | | 19.1 |
| 253 | *Melanochelys trijuga* | | | | | Geoemydidae | | | | 208 | | | | |  | | | |  | | 4 | |  |
| 254 | *Melanochelys trijuga* | | | | | Geoemydidae | | | | 229 | | | | |  | | | |  | | 3.8 | |  |
| 255 | *Melanochelys trijuga* | | | | | Geoemydidae | | | | 175 | | | | |  | | | | 760 | | 3 | | 15 |
| 256 | *Mesoclemmys dahli* | | | | | Chelidae | | | | 191 | | | | |  | | | | 777 | | 4 | | 15.16 |
| 257 | *Mesoclemmys gibba* | | | | | Chelidae | | | | 161.3 | | | | |  | | | |  | | (2 - 4) | | 25.2 |
| 258 | *Mesoclemmys nasuta* | | | | | Chelidae | | | | 253 | | | | |  | | | |  | | (6 - 8) | |  |
| 259 | *Mesoclemmys zuliae* | | | | | Chelidae | | | | 263 | | | | |  | | | | 1983 | | 7 | | 18.36 |
| 260 | *Morenia ocellata* | | | | | Geoemydidae | | | | 220 | | | | |  | | | |  | | (5 - 8) | |  |
| 261 | *Morenia petersi* | | | | | Geoemydidae | | | | 195.6  (180 - 222) | | | | |  | | | | 1010.71  (950 - 1150) | | (6 - 10) | | 10.33 |
| 262 | *Myuchelys georgesi* | | | | | Chelidae | | | | 203.9  (154 - 231) | | | | |  | | | | 977.3  (375 - 1430) | | 13.5 | |  |
| 263 | *Myuchelys georgesi* | | | | | Chelidae | | | |  | | | | |  | | | |  | |  | |  |
| 264 | *Myuchelys georgesi* | | | | | Chelidae | | | |  | | | | |  | | | |  | |  | |  |
| 265 | *Myuchelys latisternum* | | | | | Chelidae | | | | 232 | | | | |  | | | | 1377 | | 17 | | 12.05 |
| 266 | *Nilssonia gangetica* | | | | | Trionychoidea | | | | 710 | | | | |  | | | |  | | 25.35 | |  |
| 267 | *Nilssonia gangetica* | | | | | Trionychoidea | | | | 675 | | | | |  | | | | 19000 | | 30.2 | | 25.3 |
| 268 | *Nilssonia gangetica* | | | | | Trionychoidea | | | |  | | | | |  | | | |  | | 10 | |  |
| **CM** | | **CF** | | **Age** | | | | **AS** | | | **Latitude** | | | **Longitude** | | | **Source** | | | | | | |
|  | |  | |  | | | |  | | | 23.50 | | | 121.00 | | | Mao (1971) & M. A. Ewert, unpublished data in Iverson et al. (2) | | | | | | |
|  | | 2.29  (1 - 4) | |  | | | |  | | | 23.50 | | | 121.00 | | | Cheng et al. (147)* | | | | | | |
|  | | (1 - 4) | |  | | | |  | | | 23.00 | | | 108.00 | | | Artner (148)* | | | | | | |
| 52.36 | |  | |  | | | |  | | | 23.00 | | | 108.00 | | | Grosse et al. (149)* | | | | | | |
| 75 | | 2 | | (9 - 12) | | | |  | | | 35.00 | | | 139.00 | | | Fukada (1965) & Fukada & Ishihara (1974) in Iverson et al. (2); Ishihara (1986) in Lovich et al. (150) | | | | | | |
| 100.8 | |  | | (8 - 9) | | | |  | | | 25.10 | | | 121.68 | | | Chen and Lue (151) | | | | | | |
|  | |  | |  | | | |  | | | 26.44 | | | 86.41 | | | Das (152); Bonin et al. (153) | | | | | | |
| 116.319 | |  | |  | | | |  | | | 13.09 | | | 80.27 | | | Premkishore and Chandran (18) | | | | | | |
|  | |  | |  | | | |  | | | 21.00 | | | 78.00 | | | Das (1985) in Premkishore and Chandran (18) | | | | | | |
|  | |  | |  | | | |  | | | 21.00 | | | 78.00 | | | Das (1985) in Premkishore and Chandran (18) | | | | | | |
| 45 | |  | |  | | | |  | | | 27.00 | | | 78.00 | | | Moll and Moll (17) | | | | | | |
| 60.6 | |  | |  | | | |  | | | 9.00 | | | -75.00 | | | Medem (1966) in Iverson et al. (2) | | | | | | |
|  | |  | |  | | | |  | | | 4.58 | | | -53.30 | | | Bohm (154); Métrailler and Le Gratiet (1996), Mittermeier et al. (1978) & Medem (1973) in Iverson et al. (2) | | | | | | |
|  | |  | |  | | | |  | | | 4.58 | | | -53.30 | | | Bohm (2010); Vogt (110) | | | | | | |
| 128.5 | |  | |  | | | |  | | | 10.00 | | | -66.00 | | | Pritchard and Trebbau (1984) in Iverson et al. (2) | | | | | | |
|  | |  | |  | | | |  | | | 19.89 | | | 95.69 | | | Das (155); Bonin et al. (2006) | | | | | | |
| 82.64 | |  | |  | | | |  | | | 23.00 | | | 90.00 | | | Rashid and Swingland (50) | | | | | | |
|  | |  | | 7.9 | | | | 0.86 | | | -30.50 | | | 153.03 | | | Blamires et al. (89) | | | | | | |
|  | |  | |  | | | | 0.68  (0.61 - 0.74) | | | -30.50 | | | 153.03 | | | Blamires and Spencer (156) | | | | | | |
|  | |  | |  | | | | 0.63  (0.56 - 0.69) | | | -30.50 | | | 153.03 | | | Blamires and Spencer (156) | | | | | | |
| 20.49 | |  | |  | | | |  | | | -23.50 | | | 143.00 | | | Legler and Cann (1980) in Iverson et al. (2) | | | | | | |
|  | |  | |  | | | |  | | | 21.00 | | | 78.00 | | | Das (1985) in Premkishore and Chandran (18) | | | | | | |
| 764.1 | |  | |  | | | |  | | | 23.27 | | | 77.60 | | | Rao (157); Das (1991) in Iverson et al. (2) | | | | | | |
|  | |  | |  | | | |  | | | 26.00 | | | 91.00 | | | Baruah et al. (158) | | | | | | |
| **No** | **Species** | | | | | | **Family** | | | | | | **CL** | | | **PL** | | | | **BM** | **CS** | **EM** | |
| 269 | *Nilssonia hurum* | | | | | | Trionychoidea | | | | | | 342  (245 - 410) | | |  | | | | 4890  (2500 - 7700) | (20 - 30) | 15.04 | |
| 270 | *Nilssonia nigricans* | | | | | | Trionychoidea | | | | | | 436 | | |  | | | | 6971 | 20.2  (16 - 24) | 26  (22.3 - 29.3) | |
| 271 | *Nilssonia nigricans* | | | | | | Trionychoidea | | | | | |  | | |  | | | |  | 12 |  | |
| 272 | *Orlitia borneensis* | | | | | | Geoemydidae | | | | | | 475 | | |  | | | | 12400 | 12 | 94.3 | |
| 273 | *Pangshura smithii* | | | | | | Geoemydidae | | | | | | 229 | | |  | | | |  | 5.8 |  | |
| 274 | *Pangshura smithii* | | | | | | Geoemydidae | | | | | | 216.1  (195 - 240) | | |  | | | | 710  (650 - 900) | (5 - 6) | 10.9 | |
| 275 | *Pangshura smithii* | | | | | | Geoemydidae | | | | | | 190 | | |  | | | | 919 | 6.7 | 12.4 | |
| 276 | *Pangshura sylhetensis* | | | | | | Geoemydidae | | | | | |  | | |  | | | |  | 5.19 |  | |
| 277 | *Pangshura sylhetensis* | | | | | | Geoemydidae | | | | | | 200 | | |  | | | |  | (6 - 12) |  | |
| 278 | *Pangshura tecta* | | | | | | Geoemydidae | | | | | | 229 | | |  | | | |  | 4.8 |  | |
| 279 | *Pangshura tecta* | | | | | | Geoemydidae | | | | | | 211.1  (192 - 240) | | |  | | | | 990  (830 - 1120) | (8 - 14) | 10.75 | |
| 280 | *Pangshura tentoria* | | | | | | Geoemydidae | | | | | | 186.7  (96 - 240) | | |  | | | | 978.5  (320 - 1585) | 6  (4 -8) | 11.48 | |
| 281 | *Pangshura tentoria* | | | | | | Geoemydidae | | | | | |  | | |  | | | |  | 8.48 |  | |
| 282 | *Pangshura tentoria* | | | | | | Geoemydidae | | | | | | 225.9  (185 - 260) | | | 228  (188 - 248) | | | | 1583.8  (285 - 1898) | 6  (5 - 8) |  | |
| 283 | *Pangshura tentoria* | | | | | | Geoemydidae | | | | | | 227 | | | 201 | | | | 1335 | 6.5 | 19 | |
| 284 | *Pangshura tentoria* | | | | | | Geoemydidae | | | | | | 198 | | |  | | | | 1033 | 6 | 21 | |
| 285 | *Pelochelys bibroni* | | | | | | Trionychoidea | | | | | | 600 | | |  | | | |  | 20.28 |  | |
| 286 | *Pelochelys cantorii* | | | | | | Trionychidae | | | | | | (700 - 1000) | | |  | | | |  | (40 - 70) |  | |
| 287 | *Pelodiscus sinensis* | | | | | | Trionychidae | | | | | | 230 | | |  | | | | 2327.5 | 14.5 | 5.4 | |
| 288 | *Pelomedusa subrufa* | | | | | | Pelomedusidae | | | | | | 270 | | |  | | | | 2273 | 36.5 | 12.38 | |
| 289 | *Peltocephalus dumerilianus* | | | | | | Pelomedusidae | | | | | | 324 | | |  | | | | 3600 | 10.7 | 46.6 | |
| 290 | *Pelusios adansonii* | | | | | | Pelomedusidae | | | | | | 220 | | |  | | | |  | (7 - 10) |  | |
| 291 | *Pelusios castanoides* | | | | | | Pelomedusidae | | | | | | (124 - 212) | | |  | | | |  | (3 - 13) | (6.6 - 10.5) | |
| 292 | *Pelusios castanoides* | | | | | | Pelomedusidae | | | | | | 220 | | |  | | | | 800 | 25 | 8.83 | |
| 293 | *Pelusios rhodesianus* | | | | | | Pelomedusidae | | | | | | 169.33  (139 - 199) | | |  | | | |  | 14  (11 - 18) |  | |
| **CM** | | | **CF** | | **Age** | | | | **AS** | | | **Latitude** | | | **Longitude** | | | **Source** | | | | | |
| 376 | | |  | |  | | | |  | | | 23.00 | | | 90.00 | | | Rashid and Swingland (50) | | | | | |
| 525.2 | | |  | |  | | | |  | | | 22.37 | | | 91.80 | | | Ahsan et al. (1991) in Iverson et al. (2) | | | | | |
|  | | |  | |  | | | |  | | | 26.00 | | | 91.00 | | | Baruah et al. (158) | | | | | |
| 1132 | | |  | |  | | | |  | | | 4.00 | | | 101.00 | | | Moll and Moll (17) | | | | | |
|  | | |  | |  | | | |  | | | 21.00 | | | 78.00 | | | Das (1985) in Premkishore and Chandran (18) | | | | | |
| 59.95 | | |  | |  | | | |  | | | 23.00 | | | 90.00 | | | Rashid and Swingland (50) | | | | | |
| 82.8 | | | (1 - 2) | |  | | | |  | | | 31.00 | | | 70.00 | | | Minton (159); Auffenberg and Khan (160) | | | | | |
|  | | |  | |  | | | |  | | | 26.00 | | | 91.00 | | | Baruah et al. (158) | | | | | |
|  | | | 2 | |  | | | |  | | | 26.05 | | | 92.77 | | | Das et al. (161); Ernst et al. (162) | | | | | |
|  | | |  | |  | | | |  | | | 21.00 | | | 78.00 | | | Das (1985) in Premkishore and Chandran (18) | | | | | |
| 118.25 | | |  | |  | | | |  | | | 23.00 | | | 90.00 | | | Rashid and Swingland (50) | | | | | |
| 68.88 | | |  | |  | | | |  | | | 23.00 | | | 90.00 | | | Rashid and Swingland (50) | | | | | |
|  | | |  | |  | | | |  | | | 26.00 | | | 91.00 | | | Baruah et al. (158) | | | | | |
|  | | |  | |  | | | |  | | | 26.85 | | | 80.91 | | | Narain et al. (163) | | | | | |
| 123.5 | | |  | |  | | | |  | | | 27.00 | | | 78.00 | | | Bhadauria and Misra (1988) in Iverson et al. (2) | | | | | |
| 126 | | |  | |  | | | |  | | | 27.00 | | | 78.00 | | | Moll (1987) in Iverson et al. (2) | | | | | |
|  | | |  | |  | | | |  | | | 21.00 | | | 78.00 | | | Das (1985) in Premkishore and Chandran (18) | | | | | |
|  | | |  | |  | | | |  | | | 29.17 | | | 121.30 | | | Das (164) | | | | | |
| 78.33 | | |  | |  | | | |  | | | 35.00 | | | 139.00 | | | S. Ishhara pers. comm in Iverson et al. (2) | | | | | |
| 451.9 | | |  | |  | | | |  | | | -29.50 | | | 25.00 | | | Jaques (1966) & Boycott and Bourquin (1988) in Iverson et al. (2) | | | | | |
| 498.6 | | |  | |  | | | |  | | | 5.50 | | | -67.50 | | | Medem (1983) in Iverson et al. (2) | | | | | |
|  | | | (4 - 5) | |  | | | |  | | | 14.77 | | | -17.06 | | | Bour (165) | | | | | |
|  | | |  | | (2 - 3)* | | | |  | | | -4.62 | | | 55.45 | | | Gerlach (166); Gerlach (167) | | | | | |
| 22.08 | | |  | |  | | | |  | | | -27.22 | | | 31.95 | | | Boycott and Bourquin (1988) in Iverson et al. (2) | | | | | |
|  | | |  | |  | | | |  | | | -9.30 | | | 24.48 | | | Broadley and Boycott (168) | | | | | |

| **No** | **Species** | **Family** | **CL** | **PL** | **BM** | **CS** | **EM** |
| --- | --- | --- | --- | --- | --- | --- | --- |
| 294 | *Pelusios rhodesianus* | Pelomedusidae | 230 |  | 900 | 12.5 | 9.48 |
| 295 | *Pelusios sinuatus* | Pelomedusidae | 400 |  | 7000 | (7 - 30) | (18 - 21) |
| 296 | *Pelusios subniger* | Pelomedusidae | (100 - 166) |  |  | (6 - 12) | (6.8 - 9.7) |
| 297 | *Phrynops geoffroanus* | Chelidae | 350 |  |  | 13.1  (9 - 17) |  |
| 298 | *Phrynops geoffroanus* | Chelidae |  |  |  | 9.2  (7 - 14) | 17.81  (15.2 - 22) |
| 299 | *Phrynops geoffroanus* | Chelidae |  |  |  | 12.8  (10 - 16) | 18.56  (15.4 - 22.5) |
| 300 | *Phrynops gibbus* | Chelidae | 184.5 |  | 706.8 | 3 | 25.88 |
| 301 | *Phrynops hilarii* | Chelidae | 280 |  | 2380 | 18 | 18.6 |
| 302 | *Phrynops hilarii* | Chelidae |  |  |  | (1 - 23) | 21.47  (17 - 26.3) |
| 303 | *Platemys platychepala* | Chelidae | 150 |  | 325 | 1 | 27 |
| 304 | *Platysternon megacephalum* | Platysternidae | 125.1  (110.5 - 152. 9) | 103.7  (90.3 - 127.2) | 393.3  (276 - 624) | 3.6  (2 - 8) | 11 |
| 305 | *Podocnemis erythrocephala* | Podocnemididae |  |  |  | (5 - 12) |  |
| 306 | *Podocnemis erythrocephala* | Podocnemididae |  |  |  | (5 - 10) |  |
| 307 | *Podocnemis erythrocephala* | Podocnemididae |  |  |  | (5 - 14) |  |
| 308 | *Podocnemis erythrocephala* | Podocnemididae | 320 |  |  | 8.7  (2 - 16) | 17.66  (9 - 23.3) |
| 309 | *Podocnemis erythrocephala* | Podocnemididae |  |  |  | (4 - 18) |  |
| 310 | *Podocnemis expansa* | Podocnemididae |  |  |  |  |  |
| 311 | *Podocnemis expansa* | Podocnemididae | 548  (440 - 660) |  |  | 103.1  (50 -184) | 41.8  (24.9 - 51.9) |
| 312 | *Podocnemis expansa* | Podocnemididae | 660  (500 - 800) | 545  (400 - 630) | 25800  (15000 - 46000) | 91.5  (63 - 134) | 32.6 |
| 313 | *Podocnemis expansa* | Podocnemididae | 719 (620 - 790) |  | 29100  (19000 - 41000) | 106  (36 - 141) | 36.4 |
| 314 | *Podocnemis expansa* | Podocnemididae |  |  |  | 105.9 |  |
| 315 | *Podocnemis expansa* | Podocnemididae |  |  |  | 107.5 |  |

| **CM** | **CF** | **Age** | **AS** | **Latitude** | **Longitude** | **Source** |
| --- | --- | --- | --- | --- | --- | --- |
| 118.5 |  |  |  | -27.02 | 31.66 | Boycott and Bourquin 1988 in Iverson et al. (2) |
|  |  |  |  | -12.18 | 34.37 | Broadley and Boycott (169) |
|  |  | (4 - 5)* |  | -4.62 | 55.45 | Gerlach (170); Gerlach (167) |
|  |  |  |  | -11.93 | -53.47 | Junior et al. (171); Ernst et al. (162) |
| 163.85 |  |  |  | -12.00 | -64.00 | Schneider et al. (172) |
| 237.57 |  |  |  | -12.00 | -64.00 | Schneider et al. (172) |
| 77.64 |  |  |  | 5.00 | -58.00 | Grossman (1989) in Iverson et al. (2) |
| 334.8 |  |  |  | -27.00 | -64.00 | Grossman (1986) in Iverson et al. (2) |
| 257.64 |  |  |  | -30.03 | -51.25 | Bujes and Verrastro (173) |
| 27 |  |  |  | 1.00 | -74.00 | Medem (1983) in Iverson et al. (2) |
| 39.6 | 1* | 13 |  | 22.38 | 114.17 | Sung et al. (174); Sung et al. (175); Bonin et al. (153) |
|  |  |  |  | 4.00 | -74.00 | Castano – Mora (1997) in Batistella and Vogt (176) |
|  |  |  |  | 4.00 | -74.00 | Castano - Mora et al. (2003) in Batistella and Vogt (176) |
|  |  |  |  | 6.00 | -75.00 | Mittemeir and Wilson (1974) in Batistella and Vogt (176) |
| 152.65 |  |  |  | -0.40 | -65.02 | Batistella and Vogt (176); Bonin et al. (153) |
|  |  |  |  | -0.98 | -62.92 | Vogt (2001) in Batistella and Vogt (176) |
|  |  |  | 0.90 | 2.32 | -63.36 | Mogollones et al. (177) |
| 4309.58 |  |  |  | -0.83 | -71.80 | Valuenza (178) |
| 3673 |  |  |  | -1.33 | -56.75 | Alho and Padua (179) |
|  |  |  |  | -9.97 | -50.08 | Portelinha et al. (180) |
|  |  |  |  | -9.98 | -50.08 | Junior and Castro (181) |
|  |  |  |  | -9.98 | -50.08 | Junior and Castro (181) |

| **No** | **Species** | | | **Family** | | | **CL** | | **PL** | | **BM** | **CS** | **EM** |
| --- | --- | --- | --- | --- | --- | --- | --- | --- | --- | --- | --- | --- | --- |
| 316 | *Podocnemis expansa* | | | Podocnemididae | | |  | |  | |  | 94.42 |  |
| 317 | *Podocnemis expansa* | | | Podocnemididae | | |  | | 224.26 | |  | 103.4 |  |
| 318 | *Podocnemis expansa* | | | Podocnemididae | | |  | | 225.56 | |  | 104.1 |  |
| 319 | *Podocnemis lewyana* | | | Podocnemididae | | | 475 | |  | | 9599 | 16.5 | 23.1 |
| 320 | *Podocnemis lewyana* | | | Podocnemididae | | |  | |  | |  | 22 |  |
| 321 | *Podocnemis lewyana* | | | Podocnemididae | | | 401  (374 - 443) | |  | | 5600  (4600 - 7800) | 20.5  (20 - 31) | 24  (17.4 - 31.6) |
| 322 | *Podocnemis lewyana* | | | Podocnemididae | | |  | |  | |  | 18 |  |
| 323 | *Podocnemis sextuberculata* | | | Podocnemididae | | | 330 | |  | |  | 15.8  (6 - 25) | 19.6  (12 - 29) |
| 324 | *Podocnemis unifilis* | | | Podocnemididae | | |  | |  | |  | 14  (4 - 26) | 26.8  (15 - 38.2) |
| 325 | *Podocnemis unifilis* | | | Podocnemididae | | |  | |  | |  | 20.1  (7 - 35) |  |
| 326 | *Podocnemis unifilis* | | | Podocnemididae | | |  | |  | |  | 30.31  (9 - 42) |  |
| 327 | *Podocnemis unifilis* | | | Podocnemididae | | |  | |  | |  | 28.7  (6 - 48) |  |
| 328 | *Podocnemis unifilis* | | | Podocnemididae | | | 360.2  (270 - 420) | | 336.7  (266 - 420) | | 6380  (4000 - 8500) | 27.3  (22 - 41) | 24.4 |
| 329 | *Podocnemis unifilis* | | | Podocnemididae | | |  | |  | |  | 13.6 |  |
| 330 | *Podocnemis unifilis* | | | Podocnemididae | | |  | |  | |  | 12.7 |  |
| 331 | *Podocnemis vogli* | | | Podocnemididae | | | 271 | |  | | 2013 | 13.5 | 18.42 |
| 332 | *Pseudemydura umbrina* | | | Chelidae | | | 125 | |  | | 324.1 | 4.5 | 8.6 |
| 333 | *Pseudemydura umbrina* | | | Chelidae | | |  | |  | |  | (3 - 5) |  |
| 334 | *Pseudemys alabamensis* | | | Emydidae | | | 319  (259 - 358) | |  | |  | 13.3  (9 - 17) |  |
| 335 | *Pseudemys alabamensis* | | | Emydidae | | |  | |  | |  | 10.5 |  |
| 336 | *Pseudemys concinna* | | | Emydidae | | |  | | 341  (304 - 382) | |  | 16.7 |  |
| 337 | *Pseudemys concinna* | | | Emydidae | | | 289 | | 281 | | 2992 | 17 | 11.99 |
| 338 | *Pseudemys concinna* | | | Emydidae | | |  | | 242  (216 - 270) | |  | 12.6 |  |
| **CM** | | **CF** | **Age** | | **AS** | **Latitude** | | **Longitude** | | **Source** | | | |
|  | |  |  | |  | -13.30 | | -50.60 | | Alves - Junior et al. (182) | | | |
|  | |  |  | |  | -13.35 | | -50.65 | | Bonach et al. (183) | | | |
|  | |  |  | |  | -13.57 | | -50.25 | | Bonach et al. (183) | | | |
| 381.2 | |  |  | |  | 9.00 | | -75.00 | | Castano – Mora (1986) in Iverson et al. (2) | | | |
|  | |  |  | |  | 9.17 | | -74.68 | | Paez et al. (184) | | | |
| 492 | |  |  | |  | 9.23 | | -74.24 | | Corea - H et al. (185) | | | |
|  | |  |  | |  | 9.23 | | -74.24 | | Paez et al. (184) | | | |
| 308  (107 - 476) | |  |  | |  | -2.87 | | -64.90 | | Pezzuti and Vogt (186); Bonin et al. (153) | | | |
| 381.5  (106.8 - 768.5) | |  |  | |  | 0.75 | | -51.42 | | Arraes and Tavares - Dias (187) | | | |
|  | |  |  | |  | 6.00 | | -66.00 | | Escalona and Fa (188) | | | |
|  | |  |  | |  | -2.15 | | -54.63 | | Pignati et al. (189) | | | |
|  | |  |  | |  | -2.15 | | -54.63 | | Pignati et al. (189) | | | |
| 666.1 | |  |  | |  | -4.22 | | -69.93 | | Foote (1978) in Iverson et al. (2) | | | |
|  | |  |  | |  | -9.98 | | -50.08 | | Junior and Castro (181) | | | |
|  | |  |  | |  | -9.98 | | -50.08 | | Junior and Castro (181) | | | |
| 248.7 | |  | 10 | | 0.92 | 7.80 | | -66.00 | | Ramo (1982) in Iverson et al. (2) & in Shine and Iverson (32) | | | |
| 38.7 | |  | (10 - 15) | |  | -31.95 | | 115.86 | | Burbridge (1981) in Iverson et al. (2) & in Kennett (31) | | | |
|  | | 1 |  | |  | -31.95 | | 115.86 | | Kuchling and Bradshaw (1993) in Kennett (25) | | | |
|  | | (2 - 3) |  | |  | 30.75 | | -87.94 | | Nelson et al. (190) | | | |
|  | |  |  | |  | 32.00 | | -86.00 | | Lahanas (1982) in Lindeman (191) | | | |
|  | |  |  | |  | 28.10 | | -81.60 | | Jackson and Walker (1997) in Lindeman (191) | | | |
| 203.8 | |  |  | |  | 33.00 | | -81.00 | | Congdon and Gibbons (1985) in Iverson et al. (2) | | | |
|  | |  |  | |  | 34.80 | | -92.20 | | Iverson (2001) in Lindeman (191) | | | |

| **No** | **Species** | | | | **Family** | | **CL** | | **PL** | | **BM** | **CS** | **EM** | |
| --- | --- | --- | --- | --- | --- | --- | --- | --- | --- | --- | --- | --- | --- | --- |
| 339 | *Pseudemys concinna* | | | | Emydidae | |  | | 272.2  (223 - 300) | |  | 21.3 |  | |
| 340 | *Pseudemys concinna* | | | | Emydidae | |  | | 268.3  (236 - 304) | |  | 18 |  | |
| 341 | *Pseudemys floridana* | | | | Emydidae | |  | | 255  (215 - 294) | |  | 12.4 |  | |
| 342 | *Pseudemys floridana* | | | | Emydidae | |  | | 238 | | 2211 | 11.5 | 11.49 | |
| 343 | *Pseudemys floridana* | | | | Emydidae | |  | | 234.92 | |  | 11.53 |  | |
| 344 | *Pseudemys nelsoni* | | | | Emydidae | | 221.7 | |  | | 1960 | 11.43  (6 - 14) |  | |
| 345 | *Pseudemys nelsoni* | | | | Emydidae | |  | |  | |  | 12.6 |  | |
| 346 | *Pseudemys nelsoni* | | | | Emydidae | |  | |  | | 3041 |  |  | |
| 347 | *Pseudemys nelsoni* | | | | Emydidae | | 298 | | 290.2  (278 - 303) | | 4020 | 14.3  (7 - 26) | 10.23 | |
| 348 | *Pseudemys peninsularis* | | | | Emydidae | | 264 | |  | | 2690 | 13.75  (9 - 21) |  | |
| 349 | *Pseudemys peninsularis* | | | | Emydidae | |  | | 287.6  (250 - 332) | | 3920 | 16.8  (10 - 23) | 13.09  (9.65 - 16.29) | |
| 350 | *Pseudemys peninsularis* | | | | Emydidae | |  | |  | | 4225 |  |  | |
| 351 | *Pseudemys rubriventris* | | | | Emydidae | | 304 | |  | | 3477 | 17 | 11.9 | |
| 352 | *Pseudemys texana* | | | | Emydidae | | 245.9  (223 - 274) | | 224.1  (213 - 241) | |  | 8.4  (7 - 9) |  | |
| 353 | *Rafetus euphraticus* | | | | Trionychoidea | |  | |  | |  | 37 |  | |
| 354 | *Rafetus euphraticus* | | | | Trionychoidea | | 680 | |  | |  | 32 | 13.6  (13 - 14.2) | |
| 355 | *Rheodytes leukops* | | | | Chelidae | | 249 | |  | | 1691 | 18.8 | 7.5 | |
| 356 | *Rhinoclemmys areolata* | | | | Geoemydidae | | 166.6 | |  | | 731.5 | 1 | 33.58 | |
| 357 | *Rhinoclemmys diadermata* | | | | Geoemydidae | | 203 | |  | | 1126 | 2 | 33.5 | |
| 358 | *Rhinoclemmys funurea* | | | | Geoemydidae | | 273 | |  | | 1946 | 3.2 | 47.2 | |
| 359 | *Rhinoclemmys melanosterna* | | | | Geoemydidae | | 243 | |  | | 2700 | 5 | 37.4 | |
| 360 | *Rhinoclemmys nasuta* | | | | Geoemydidae | | 218 | |  | | 1284 | 1 | 52.7 | |
| 361 | *Sacalia quadriocellata** | | | | Geoemydidae | | 145 | |  | |  | 2.47  (1 -4) | 12.8  (8.9 - 16.9) | |
| 362 | *Siebenrockiella crassicollis* | | | | Geoemydidae | | 186 | |  | | 940 | 1.3 | 28.5 | |
| **CM** | | **CF** | **Age** | **AS** | | **Latitude** | | **Longitude** | | **Source** | | | |  |
|  | |  |  |  | | 38.50 | | -92.50 | | Turner (1995) in Lindeman (191) | | | |  |
|  | |  |  |  | | 38.50 | | -92.50 | | Turner (1995) in Lindeman (191) | | | |  |
|  | |  |  |  | | 30.53 | | -84.32 | | Aresco (2004) in Lindeman (191) | | | |  |
| 132.1 | |  |  |  | | 33.00 | | -81.00 | | Congdon and Gibbons (7) | | | |  |
|  | |  |  |  | | 33.00 | | -81.00 | | Wilkinson and Gibbons (135) | | | |  |
|  | |  | (7 - 8) |  | | 28.47 | | -81.35 | | Bancroft et al. (192) | | | |  |
|  | |  |  |  | | 28.10 | | -81.60 | | Kushlan and Kushlan (1980) in Lindeman (191) | | | |  |
|  | |  |  | 0.61 | | 28.70 | | -81.45 | | Hrycyshyn (193) | | | |  |
| 146.3 | | (3 - 6) | 8 |  | | 29.65 | | -82.33 | | Jackson (1988) in Iverson et al. (2) | | | |  |
|  | | 2 | (15 - 16) |  | | 28.47 | | -81.35 | | Bancroft et al. (192) | | | |  |
| 219.9 | | (3 - 6) |  |  | | 29.65 | | -82.33 | | Jackson (1988) in Iverson et al. (2) | | | |  |
|  | |  |  | 0.65  (0.506 - 0.835) | | 28.70 | | -81.45 | | Hrycyshyn (193) | | | |  |
| 202.3 | | 2 | 10 |  | | 41.96 | | -70.67 | | Graham (1971a, 1971b) in Iverson et al. (2) | | | |  |
|  | |  |  |  | | 30.45 | | -99.81 | | Lindeman (191) | | | |  |
|  | |  |  |  | | 32.56 | | 53.00 | | Ghaffari et al. (194) | | | |  |
| 435.2 | |  |  |  | | 37.00 | | 32.00 | | Biricik and Turga (195); Ernst et al. (162) | | | |  |
| 121 | | (2 - 3) |  |  | | -23.50 | | 150.79 | | Legler and Cann (1980) in Iverson et al. (2) & in Kennett (25) | | | |  |
| 33.58 | |  |  |  | | 19.00 | | -89.00 | | M.A. Ewert & J. B. Iverson, unpublished data in Iverson et al. (2) | | | |  |
| 66.97 | |  |  |  | | 10.00 | | -66.00 | | Pritchard and Trebbau (1984) in Iverson et al. (2) | | | |  |
| 141 | |  |  |  | | 9.00 | | -79.00 | | Moll and Legler (1971) in Iverson et al. (2) | | | |  |
| 187 | |  |  |  | | 8.97 | | -79.53 | | Moll and Moll (1990) in Iverson et al. (2) | | | |  |
| 52.7 | |  | 12 |  | | 5.00 | | -74.00 | | Medem (1962a, 1962b) & Perez unpublished data in Iverson et al. (2) | | | |  |
| 31.616 | | 1 |  |  | | 19.20 | | 109.75 | | Bin et al. (196)*; Ernst et al. (162) | | | |  |
| 37 | |  |  |  | | 4.00 | | 101.00 | | Moll and Moll (1990) in Iverson et al. (2) | | | |  |

| **No** | **Species** | | | | | | **Family** | | | | **CL** | | | | | **PL** | | | | | **BM** | | **CS** | | **EM** |  |
| --- | --- | --- | --- | --- | --- | --- | --- | --- | --- | --- | --- | --- | --- | --- | --- | --- | --- | --- | --- | --- | --- | --- | --- | --- | --- | --- |
| 363 | *Siebenrockiella leytensis* | | | | | | Geoemydidae | | | | 190.6  (172 - 212) | | | | | 161.1  (140 - 178) | | | | | 1081.1  (770 - 1400) | | (1 - 2) | | 24  (18 - 30) |  |
| 364 | *Staurotypus salvinii* | | | | | | Staurotypidae | | | | 180 | | | | |  | | | | | 900 | | 5.36 | | 10.57 |  |
| 365 | *Staurotypus triporcatus* | | | | | | Staurotypidae | | | | 285 | | | | |  | | | | | 4200 | | 10.61 | | 16.24 |  |
| 366 | *Sternotherus carinatus* | | | | | | Kinosternidae | | | | 102  (86 - 121) | | | | | 74  (60 - 85) | | | | | 161  (98 - 238) | | 2.3  (1 - 3) | | 5.2  (3.76 - 6.41) |  |
| 367 | *Sternotherus carinatus* | | | | | | Kinosternidae | | | | 117  (95 - 155) | | | | | 88  (72 - 119) | | | | | 237  (136 - 348) | | 3.8  (2 - 7) | | 5.7  (3.77 - 7.1) |  |
| 368 | *Sternotherus depressus* | | | | | | Kinosternidae | | | | 95 | | | | |  | | | | | 113 | | 2.2 | | 5.54 |  |
| 369 | *Sternotherus depressus* | | | | | | Kinosternidae | | | | 89.8 | | | | |  | | | | |  | | (1 - 3) | | 5.5 |  |
| 370 | *Sternotherus minor* | | | | | | Kinosternidae | | | | 105.1 | | | | |  | | | | | 165 | | 3  (1 - 5) | | 5.27 |  |
| 371 | *Sternotherus minor* | | | | | | Kinosternidae | | | |  | | | | |  | | | | | 106 | |  | |  |  |
| 372 | *Sternotherus minor* | | | | | | Kinosternidae | | | | 100 | | | | |  | | | | | 144 | | 2.46 | | 5.25 |  |
| 373 | *Sternotherus odoratus* | | | | | | Kinosternidae | | | |  | | | | |  | | | | | 67 | |  | |  |  |
| 374 | *Sternotherus odoratus* | | | | | | Kinosternidae | | | |  | | | | | 78.2 | | | | |  | | 4.98 | |  |  |
| 375 | *Sternotherus odoratus* | | | | | | Kinosternidae | | | | 108.5 | | | | |  | | | | | 181 | | 4.5 | | 4.04 |  |
| 376 | *Sternotherus odoratus* | | | | | | Kinosternidae | | | | 85.9 | | | | |  | | | | | 94.8 | | 3.05 | | 2.66 |  |
| 377 | *Sternotherus odoratus* | | | | | | Kinosternidae | | | | 104 | | | | |  | | | | | 187.7 | |  | |  |  |
| 378 | *Sternotherus odoratus* | | | | | | Kinosternidae | | | |  | | | | |  | | | | |  | | 4  (2 - 7) | |  |  |
| 379 | *Sternotherus odoratus* | | | | | | Kinosternidae | | | | (83 - 128) | | | | |  | | | | |  | |  | |  |  |
| 380 | *Terrapene carolina* | | | | | | Emydidae | | | |  | | | | |  | | | | |  | |  | |  |  |
| 381 | *Terrapene carolina* | | | | | | Emydidae | | | | 133.2  (104 - 153) | | | | | 117.6  (90 - 138) | | | | | 444.2  (195 - 605) | | 2.4  (1 - 5) | |  |  |
| 382 | *Terrapene carolina* | | | | | | Emydidae | | | | 127.92 | | | | |  | | | | | 404.82 | | 3.07  (2 - 5) | |  |  |
| 383 | *Terrapene carolina* | | | | | | Emydidae | | | | 134 | | | | | 129 | | | | | 372 | | 3.4 | | 9.02 |  |
| 384 | *Terrapene carolina* | | | | | | Emydidae | | | | 161.9 | | | | |  | | | | |  | |  | |  |  |
| 385 | *Terrapene carolina* | | | | | | Emydidae | | | |  | | | | |  | | | | |  | |  | |  |  |
| 386 | *Terrapene carolina* | | | | | | Emydidae | | | |  | | | | |  | | | | |  | |  | |  |  |
| 387 | *Terrapene carolina* | | | | | | Emydidae | | | | 115 | | | | |  | | | | |  | |  | |  |  |
| **CM** | | | **CF** | **Age** | | | | **AS** | | | | **Latitude** | | | **Longitude** | | | | **Source** | | | | | | |  |
|  | | |  |  | | | |  | | | | 10.53 | | | 119.29 | | | | Diesmos et al. (197) | | | | | | |  |
| 56.66 | | |  |  | | | |  | | | | 17.00 | | | -99.00 | | | | M. Goode (1991) in Iverson et al. (2) | | | | | | |  |
| 172.31 | | |  |  | | | |  | | | | 18.00 | | | -99.00 | | | | M. Goode (1991) in Iverson et al. (2) | | | | | | |  |
| 11.6  (3.8 - 17.8) | | | 2.6  (2 - 4) | (5 - 6) | | | |  | | | | 34.02 | | | -94.00 | | | | Iverson (198) | | | | | | |  |
| 21.6  (10.6 - 40.5) | | | 2.4  (1 - 3) | 8 | | | |  | | | | 34.58 | | | -93.00 | | | | Iverson (198) | | | | | | |  |
| 12.19 | | |  |  | | | |  | | | | 33.00 | | | -86.00 | | | | Estridge (1970), Marion (1983) & Dodd et al. (1988) in Iverson et al. (2) | | | | | | |  |
|  | | | 2 | (6 - 8) | | | |  | | | | 33.78 | | | -87.11 | | | | Dodd (199) | | | | | | |  |
| 15.81 | | | 4 | 6 | | | |  | | | | 28.00 | | | -81.00 | | | | Etchberger and Ehrhart (1987) in Iverson et al. (2) | | | | | | |  |
|  | | |  |  | | | | 0.45 | | | | 28.70 | | | -81.45 | | | | Hrycyshyn (193) | | | | | | |  |
| 12.92 | | | 3 | 5 | | | |  | | | | 30.00 | | | -83.00 | | | | Iverson (1978) in in Iverson et al. (2) | | | | | | |  |
|  | | |  |  | | | | 0.46 | | | | 28.70 | | | -81.45 | | | | Hrycyshyn (193) | | | | | | |  |
|  | | |  |  | | | |  | | | | 33.00 | | | -81.00 | | | | Wilkinson and Gibbons (135) | | | | | | |  |
| 18.4 | | |  |  | | | |  | | | | 33.00 | | | -81.00 | | | | Congdon and Gibbons (1985) in Iverson et al. (2) | | | | | | |  |
| 8.11 | | | 3 | 4 | | | | 0.84 | | | | 38.00 | | | -77.00 | | | | Mithcell, (1985b, 1988) in Iverson et al. (2) & in Shine and Iverson (32) | | | | | | |  |
|  | | |  |  | | | | 0.99 | | | | 38.23 | | | -85.47 | | | | Attum et al. (200) | | | | | | |  |
|  | | | 1 |  | | | |  | | | | 41.00 | | | -86.00 | | | | Baker et al. (14) | | | | | | |  |
|  | | |  |  | | | | 0.92  (0.75 - 1) | | | | 45.49 | | | -76.39 | | | | Bellau (201) | | | | | | |  |
|  | | |  |  | | | | 0.87 | | | | 27.60 | | | -82.76 | | | | Dodd et al. (202) | | | | | | |  |
|  | | | 2.14  (1 - 3) |  | | | |  | | | | 27.60 | | | -82.76 | | | | Dodd (203) | | | | | | |  |
|  | | |  |  | | | |  | | | | 30.74 | | | -95.57 | | | | Buchman et al., (204) | | | | | | |  |
| 30.7 | | |  |  | | | |  | | | | 33.00 | | | -81.00 | | | | Congdon and Gibbons (1985) in Iverson et al. (2) | | | | | | |  |
|  | | |  | 8 | | | |  | | | | 35.50 | | | -98.00 | | | | Clair (205) | | | | | | |  |
|  | | |  |  | | | | (0.93 - 0.94) | | | | 38.00 | | | -76.00 | | | | Stickel (1978) in Iverson (45) | | | | | | |  |
|  | | |  |  | | | | 0.92  (0.813 - 0.977) | | | | 38.50 | | | -75.67 | | | | Nazdrowicz et al. (206) | | | | | | |  |
|  | | |  | 10 | | | | 0.86  (0.61 - 1) | | | | 38.50 | | | -92.50 | | | | Schwartz et al. (207) | | | | | | |  |
| **No** | **Species** | | | | | **Family** | | | | **CL** | | | | **PL** | | | | **BM** | | | | **CS** | | **EM** | |  |
| 388 | *Terrapene carolina* | | | | | Emydidae | | | |  | | | |  | | | |  | | | |  | |  | |  |
| 389 | *Terrapene carolina* | | | | | Emydidae | | | |  | | | |  | | | |  | | | |  | |  | |  |
| 390 | *Terrapene carolina* | | | | | Emydidae | | | | 129 | | | |  | | | |  | | | | 4.1  (1 - 6) | |  | |  |
| 391 | *Terrapene coahuila* | | | | | Emydidae | | | | 101.6 | | | |  | | | | 259.8 | | | | 2.3 | | 5.66 | |  |
| 392 | *Terrapene nelsoni* | | | | | Emydidae | | | | 134 | | | |  | | | | 372 | | | | 2.7 | | 18.46 | |  |
| 393 | *Terrapene ornata* | | | | | Emydidae | | | | 120 | | | |  | | | |  | | | |  | |  | |  |
| 394 | *Terrapene ornata* | | | | | Emydidae | | | | 118.46  (117.9 - 119.2) | | | |  | | | |  | | | | 2.93  (1 - 5) | |  | |  |
| 395 | *Terrapene ornata* | | | | | Emydidae | | | | 119  (107.8 - 127.9) | | | |  | | | | 447.3  (327 - 590) | | | | 2.68  (1 - 4) | |  | |  |
| 396 | *Terrapene ornata* | | | | | Emydidae | | | | 135.3 | | | |  | | | |  | | | |  | |  | |  |
| 397 | *Terrapene ornata* | | | | | Emydidae | | | | 117 | | | |  | | | | 391 | | | | 4.7 | | 10.09 | |  |
| 398 | *Terrapene ornata* | | | | | Emydidae | | | | 110.52 | | | | 115.71 | | | | 351.87 | | | | 2.6  (2 - 4) | |  | |  |
| 399 | *Terrapene ornata* | | | | | Emydidae | | | |  | | | |  | | | |  | | | |  | |  | |  |
| 400 | *Terrapene ornata* | | | | | Emydidae | | | |  | | | |  | | | |  | | | | 3.5 | |  | |  |
| 401 | *Terrapene ornata* | | | | | Emydidae | | | | 117.8 | | | | 123.9 | | | |  | | | | 4.3  (2 - 5) | |  | |  |
| 402 | *Trachemys callirostris* | | | | | Emydidae | | | | (190 - 250) | | | |  | | | |  | | | | 11  (5 - 21) | |  | |  |
| 403 | *Trachemys decorata* | | | | | Emydidae | | | | 261 | | | |  | | | | 2522 | | | | 12 | | 16.8 | |  |
| 404 | *Trachemys decussata* | | | | | Emydidae | | | | 171 | | | |  | | | | 630.1 | | | | 5 | | 13.3 | |  |
| 405 | *Trachemys dorbigni* | | | | | Emydidae | | | | 260 | | | |  | | | | 2497 | | | | 14 | | 18.4 | |  |
| 406 | *Trachemys dorbigni* | | | | | Emydidae | | | | 235  (214 - 278) | | | |  | | | |  | | | | 12.1  (6 - 19) | | 14.9  (11.8 - 17.8) | |  |
| 407 | *Trachemys dorbigni* | | | | | Emydidae | | | | 205.9  (176 - 239) | | | |  | | | |  | | | | 8.2  (4 - 15) | |  | |  |
| 408 | *Trachemys gaigeae* | | | | | Emydidae | | | | (228 - 266) | | | |  | | | |  | | | | (6 - 29) | | 10.7 | |  |
| 409 | *Trachemys scripta* | | | | | Emydidae | | | |  | | | | 210.3  (183 - 230) | | | | 1950 | | | | 9.7  (4 - 15) | | 10.99  (8.7 - 12.83) | |  |
| 410 | *Trachemys scripta* | | | | | Emydidae | | | |  | | | |  | | | |  | | | |  | |  | |  |
| 411 | *Trachemys scripta* | | | | | Emydidae | | | | 258 | | | |  | | | |  | | | | 6.25  (5 - 15) | |  | |  |
| **CM** | | **CF** | | | **Age** | | | | **AS** | | | | **Latitude** | | | | **Longitude** | | | **Source** | | | | | | |
|  | |  | | |  | | | | 0.964  (0.954 - 0.978) | | | | 39.32 | | | | -86.41 | | | Currylow et al. (208) | | | | | | |
|  | |  | | |  | | | | (0.932 - 0.934) | | | | 39.78 | | | | -87.21 | | | Williams and Parker (1987) in Iverson (45) | | | | | | |
|  | | 1 | | |  | | | |  | | | | 40.78 | | | | -72.88 | | | Burke and Capitano (209) | | | | | | |
| 13.02 | | 3 | | |  | | | |  | | | | 26.92 | | | | -102.13 | | | Brown (1974) in Iverson et al. (2) | | | | | | |
| 49.9 | |  | | |  | | | |  | | | | 22.00 | | | | -105.23 | | | Milstead and Tinkle (210); Milstead (211) | | | | | | |
|  | |  | | | 9 | | | | 0.91 | | | | 31.00 | | | | -100.00 | | | Blair (1976) in Shine and Iverson (32) | | | | | | |
|  | |  | | |  | | | |  | | | | 34.33 | | | | -106.83 | | | Germano (212) | | | | | | |
|  | |  | | |  | | | |  | | | | 34.33 | | | | -106.83 | | | Nieuwolt-Dacanay (213) | | | | | | |
|  | |  | | | 8 | | | |  | | | | 35.50 | | | | -98.00 | | | Clair (205) | | | | | | |
| 47.4 | | 1.3 | | | 10.5 | | | | 0.83 | | | | 39.00 | | | | -104.00 | | | Legler (1960), Metcalf and Metcalf (1985) in Iverson et al. (2) | | | | | | |
|  | |  | | |  | | | | 0.93 | | | | 41.00 | | | | -102.00 | | | Converse et al. (214); Converse et al. (215) | | | | | | |
|  | |  | | |  | | | | 0.99  (0.87 - 1) | | | | 42.88 | | | | -91.10 | | | Bowen et al. (216) | | | | | | |
|  | |  | | |  | | | | 0.81 | | | | 43.00 | | | | -89.00 | | | Doroff and Keith (217) | | | | | | |
|  | |  | | | 9 | | | |  | | | | 43.33 | | | | -102.55 | | | Quinn et al. (218) | | | | | | |
|  | |  | | |  | | | |  | | | | 9.23 | | | | -74.24 | | | Corea – H (2006) in Bock et al. (219) | | | | | | |
| 201.3 | |  | | |  | | | |  | | | | 18.00 | | | | -71.21 | | | Inchausteguj Mirando (1973) in Iverson et al. (2) | | | | | | |
| 66.5 | |  | | |  | | | |  | | | | 22.35 | | | | -81.67 | | | Sampedro Marin et al. (1983) in Iverson et al. (2) | | | | | | |
| 257.4 | |  | | |  | | | |  | | | | -30.00 | | | | -58.00 | | | Freiburg (1971) in Iverson et al. (2) | | | | | | |
| 180.29 | | 1.44  (1 - 3) | | |  | | | |  | | | | -32.54 | | | | -52.54 | | | Bager et al. (220) | | | | | | |
|  | |  | | |  | | | |  | | | | -31.77 | | | | -52.37 | | | Fagundes et al. (221) | | | | | | |
|  | |  | | |  | | | |  | | | | 33.77 | | | | -106.90 | | | Morjan and Stuart (222); Stuart & Painter (1997) in Stuart and Ward (223) | | | | | | |
| 108.8 | | 4 | | |  | | | |  | | | | 30.00 | | | | -82.00 | | | Jackson (72) | | | | | | |
|  | |  | | |  | | | | 0.79 | | | | 33.00 | | | | -81.00 | | | Gibbons and Semlitsch (1982) in Iverson (45) | | | | | | |
|  | | 1.1  (1 - 2) | | | 8 | | | | 0.81 | | | | 33.00 | | | | -81.00 | | | Frazer et al. (224); Frazer et al. (225) | | | | | | |

| **No** | **Species** | **Family** | **CL** | **PL** | **BM** | **CS** | **EM** |
| --- | --- | --- | --- | --- | --- | --- | --- |
| 412 | *Trachemys scripta* | Emydidae |  | 205 | 1895 | 7.1 | 10.52 |
| 413 | *Trachemys scripta* | Emydidae |  |  |  |  |  |
| 414 | *Trachemys scripta* | Emydidae | 188.17 | 171.51 | 971.69 |  |  |
| 415 | *Trachemys scripta* | Emydidae | 177.83 | 164.45 | 843.1 |  |  |
| 416 | *Trachemys scripta* | Emydidae | 194 | 179.78 | 1110.16 |  |  |
| 417 | *Trachemys scripta* | Emydidae |  | 212  (172 -238) | 1790  (1100 - 2700) | 13.6  (8 - 27) | 10.5  (6.04 - 13.55) |
| 418 | *Trachemys scripta* | Emydidae |  | 214  (167 - 240) | 1910  (980 - 2600) | 14.9  (6 - 26) | 10.64  (6.41 - 13.45) |
| 419 | *Trachemys scripta* | Emydidae |  | 215  (170 - 251) | 1870  (1000 - 2850) | 13.5  (6 - 26) | 10.03  (6.25 - 13.26) |
| 420 | *Trachemys scripta* | Emydidae |  | 214  (200 -233) | 1780  (1350 - 2250) | 13.3  (11 - 17) | 10.22  (9.02 - 11.85) |
| 421 | *Trachemys scripta* | Emydidae |  | 212  (182 - 250) | 1840  (1200 - 3300) | 12.5  (8 - 20) | 10.76  (7.71 - 12.75) |
| 422 | *Trachemys scripta* | Emydidae |  | 216  (191 - 250) | 1980  (1350 - 3130) | 15.1  (9 - 30) | 11.68  (8.49 - 14.93) |
| 423 | *Trachemys scripta* | Emydidae |  | 211  (181 - 240) | 1870  (1130 - 2630) | 14.2  (8 - 22) | 11.21  (6.91 - 13.23) |
| 424 | *Trachemys scripta* | Emydidae |  | 214  (187 - 258) | 1790  (1100 - 2650) | 12.7  (7 - 21) | 10.74  (7.91 - 14.02) |
| 425 | *Trachemys scripta* | Emydidae |  | 215  (185 - 248) | 1970  (1350 - 2800) | 13  (6 - 23) | 11.13  (8.03 - 15.07) |
| 426 | *Trachemys scripta* | Emydidae |  |  |  | 13  (7 - 22) |  |
| 427 | *Trionyx triunguis* | Trionychidae | 506 |  | 10818 | 40 | 17.82 |
| 428 | *Trionyx triunguis* | Trionychoidea |  |  |  | 31 |  |
| 429 | *Vijayachelys silvatica* | Geoemydidae | 116.9  (85. 5 - 170) | 103.7  (77 - 160) | 190.9  (76.5 - 280) | (1 - 4) | 15.74  (13.5 - 22.2) |
| 430 | *Vijayachelys silvatica* | Geoemydidae | 121 | 108 | 230 | 2 | 15.25 |

| **CM** | **CF** | **Age** | **AS** | **Latitude** | **Longitude** | **Source** |
| --- | --- | --- | --- | --- | --- | --- |
| 74.7 | 3 | 7 |  | 33.00 | -81.00 | Gibbons et al. (226); Congdon and Gibbons (7) |
|  |  |  | 0.73 | 35.23 | -80.84 | Eskew et al. (42) |
|  |  |  | 0.74 | 35.44 | -95.02 | Hays and McBee (227) |
|  |  |  | 0.86 | 36.08 | -97.23 | Hays and McBee (227) |
|  |  |  | 0.86 | 36.84 | -94.81 | Hays and McBee (227) |
| 142  (48.8 - 300.8) |  |  |  | 38.99 | -90.00 | Tucker et al. (228) |
| 158.7  (52.5 - 292.6) |  |  |  | 38.99 | -90.00 | Tucker et al. (228) |
| 136.3  (43 - 295.6) |  |  |  | 38.99 | -90.00 | Tucker et al. (228) |
| 135.8  (102.4 - 189.1) |  |  |  | 39.16 | -90.00 | Tucker et al. (228) |
| 134.4  (77.1 - 225.8) |  |  |  | 39.16 | -90.00 | Tucker et al. (228) |
| 177.3  (96.5 - 362.7) |  |  |  | 39.16 | -90.00 | Tucker et al. (228) |
| 159.2  (68.4 - 233.8) |  |  |  | 39.16 | -90.00 | Tucker et al. (228) |
| 136.8  (75.5 - 277) |  |  |  | 39.16 | -90.00 | Tucker et al. (228) |
| 144.7  (64.2 - 234.5) |  |  |  | 39.16 | -90.00 | Tucker et al. (228) |
|  | (1 - 2) |  |  | 41.00 | -86.00 | Baker et al. (14) |
| 712.8 |  |  |  | 31.00 | 35.00 | Atatur (1979) &; Leshem & Dmi'el (1987) in Iverson et al. (2) |
|  |  |  |  | 36.77 | 28.80 | Gidis and Kaska (229) |
| 39.35 | (1 - 2) |  |  | 8.51 | 76.97 | Whitaker and Vijaya (230) |
| 30.5 |  |  |  | 10.37 | 77.13 | Moll et al. (231) |

**References**

1. Vinke T, Vinke S, Richard E, Cabrera MR, Pazko L, Marano P, et al. *Acanthochelys pallidipectoris* (Freiberg 1945) - Chaco Side-Necked Turtle. In: Rhodin AGJ, Pritchard PCH, van Dijk PP, Saumure RA, Buhlmann KA, Iverson JB, et al., editors. Conservation Biology of Freshwater Turtles and Tortoises: A Compilation Project of the IUCN/SSC Tortoise and Freshwater Turtle Specialist Group. 5: Chelonian Research Monographs; 2011. p. 065.1 - .7.

2. Iverson JB, Balgooyen CP, Byrd KK, Lyddan KK. Latitudinal Variation in Egg and Cutch Size in Turtles. Canadian Journal of Zoology. 1993;71:2448 - 61.

3. Mocelin MA, Fernandes R, Porto M, Fernandes DS. Reproductive Biology and Notes on Natural History of the Side-Necked Turtle *Acanthochelys radiolata* (Mikan, 1820) in Captivity (Testudines: Chelidae). South American Journal of Herpetology. 2008;3(3):223 - 8.

4. Fraxe Neto HJ, Brasil MA, de Freitas Horta G, Barros TO, Falcon GB, Colli GR. Demography of *Acanthochelys spixii* (Testudines, Chelidae) in the Brazilian Cerrado. Chelonian Conservation and Biology. 2011;10(1):82 - 90.

5. Germano DJ, Rathbun GB. Growth, Population Structure, and Reproduction of Western Pond Turtles (*Actinemys marmorata*) on the Central Coast of California. Chelonian Conservation and Biology. 2008;7(2):188 - 94.

6. Lovich JE, Meyer K. The Western Pond Turtle (*Clemmys marmorata*) in the Mojave River, California, USA: Highly Adapted Survivor or Tenuous Relict? Journal of Zoology. 2002;256:537 - 45.

7. Congdon JD, Gibbons JW. Egg Components and Reproductive Characteristics of Turtles: Relationships to Body Size. Herpetologica. 1985;41(2):194 - 205.

8. Scott NJ, Rathbun GB, Murphey TG, Harker MB. Reproduction of Pacific Pond Turtles (*Actinemys marmorata*) in Coastal Streams of Central California. Herpetological Conservation and Biology. 2008;3(2):143 - 8.

9. Germano DJ, Bury RB. Variation in Body Size, Growth, and Population Structure of *Actinemys marmorata* from Lentic and Lotic Habitats in Southern Oregon. Journal of Herpetology. 2009;43(3):510 - 20.

10. Haegen WMV, Clark SL, Perillo KM, Anderson DP, Allen HI. Survival and Causes of Mortality of Head-Started Western Pond Turtles on Pierce National Wildlife Refuge, Washington. The Journal of Wildlife Management. 2009;73(8):1402 - 6.

11. Iverson JB. Reproduction in the Florida Softshell Turtle, *Trionyx ferox* Florida Scientist. 1985;48(1):37 - 41.

12. Fitch HS, Plummer MV. A Preliminary Ecological Study of Soft-shelled Turtle, *Trionyx muticus*, in the Kansas River. Israel Journal of Zoology. 1975;24:28 - 42.

13. Plummer MV, Krementz DG, Powell LA, Mills NE. Effects of Habitat Disturbance on Survival Rates of Softshell Turtles (*Apalone spinifera*) in an Urban Stream. Journal of Herpetology. 2008;42(3):555 - 63.

14. Baker PJ, Costanzo JP, Iverson JB, Lee Jr RE. Seasonality and Interspecific and Intraspecific Asynchrony in Emergence From the Nest by Hatchling Freshwater Turtles. Canadian Journal of Zoology. 2013;91:451 - 61.

15. Chan EH, Chen PN. Nesting Activity and Clutch Size of *Batagur affinis edwardmolli* from the Setiu River, Trengganu, Malaysia. Chelonian Conservation and Biology. 2011;10(1):129 - 32.

16. Cox MJ, van Dijk PP, Nabhitabhata J, Thirakhupt K. A Photographic Guide to Snakes and Other Reptiles of Peninsular Malaysia, Singapore and Thailand. 2nd ed. London: New Holland Publishers; 2010.

17. Moll D, Moll EO. The Slider Turtle in the Neotropics: Adaptation of a Temperate Species to a Tropical Environment. In: Gibbons JW, editor. Life History and Ecology of the Slider Turtle. Washington, D. C.: Smithsonian Institution Press; 1990. p. 152 - 61.

18. Premkishore G, Chandran MR. Nesting Studies of Two Freshwater Turtles (*Lissemys punctata punctata* and *Melanochelys trijuga trijuga*) of Tamil Nadu, India, in the Context of Their Conservation. Annales des Sciences Naturelles Zoologie et Biologie Animale. 1996;17(3):99 - 104.

19. Moll EO, Platt K, Platt SG, Praschag P, van Dijk PP. *Batagur baska* (Gray 1830) - Northern River Terrapin. In: Rhodin AGJ, Pritchard PCH, van Dijk PP, Saumure RA, Buhlmann KA, Iverson JB, et al., editors. Conservation Biology of Freshwater Turtles and Tortoises: A Compilation Project of the IUCN/SSC Tortoise and Freshwater Turtle Specialist Group. 5: Chelonian Research Monographs; 2009. p. 037.1 - .10.

20. Eisemberg CC, Rose M, Yaru B, Georges A. Demonstrating Decline of an Iconic Species Under Sustained Indigenous Harvest - The Pig - Nosed Turtle (*Carettochelys insculpta*) in Papua New Guinea. Biological Conservation. 2011;144:2282 - 8.

21. Georges A, Alacs E, Pauza M, Kinginapi F, Ona A, Eisemberg C. Freshwater Turtles of the Kikori Drainage, Papua New Guinea, With Special Reference to the Pig-Nosed Turtle, *Carettochelys insculpta*. Wildlife Research. 2008;35:700 - 11.

22. Georges A, Kennett R. Dry-Season Distribution and Ecology of *Carretochelys insculpta* (Chelonia: Carrethochelydidae) in Kakadu National Park, Northern Australi. Australian Wildlife Research. 1989;16:323 - 35.

23. Doody JS, Georges A, Young JE. Twice Every Second year: Reproduction in the Pig-nosed Turtle, *Carettochelys insculpta*, in the Wet-dry Tropics of Australia. Journal of Zoology. 2003;259:179 - 88.

24. Kennett RM, Georges A, Thomas K, Georges TC. Distribution of the Long-necked Freshwater Turtle *Chelodina novaeguineae* and New Information on Its Ecology. Memoirs of the Queensland Museum. 1992;32:179 - 82.

25. Kennett R. Reproduction of two species of freshwater turtle, *Chelodina rugosa* and *Elseya dentata*, from the wet-dry tropics of northern Australia. Journal of Zoology. 1999;247:457 - 73.

26. Kuchling G. Gonadal cycles of the western Australian long-necked turtles *Chelodina oblonga* and *Chelodina steindachneri* (Chelonia: Chelidae). Records of the Western Australian Museum. 1988;14(2):189 - 98.

27. Clay BT. Observations on the Breeding Biology and Behaviour of the Long-necked Tortoise, *Chelodina oblonga*. Journal of the Royal Society of Western Australia. 1981;4(1):27 - 32.

28. Goode J, Russell J. Incubation of Eggs of Three Species of Chelid Tortoises, and Notes on Their Embryological Development. Australian Journal of Zoology. 1968;16(5):749 - 61.

29. Spencer RJ. Growth Patterns of Two Widely Distributed Freshwater Turtles and a Comparison of Common Methods Used to Estimate Age. Australian Journal of Zoology. 2002;50:477 - 90.

30. Spencer RJ, Thompson MB. Experimental Analysis of the Impact of Foxes on Freshwater Turtle Populations. Conservation Biology. 2005;19(3):845 - 54.

31. Kennett R. Growth Models for Two Species of Freshwater Turtle, *Chelodina rugosa* and *Elseya dentata*, From the Wet-Dry Tropics of Northern Australia. Herpetologica. 1996;52(3):383 - 95.

32. Shine R, Iverson JB. Patterns of survival, growth and maturation in turtles. OIKOS. 1995;72:343 - 8.

33. Kennett R, Georges A. Habitat Utilization and Its Relationship to Growth and Reproduction of the Eastern Long-Necked Turtle, *Chelodina longicollis* (Testudinata: Chelidae) From Australia. Herpetologica. 1990;46(1):22 - 33.

34. Roe JH, Brinton AC, Georges A. Temporal and Spatial Variation in Landscape Connectivity For A Freshwater Turtle in A Temporally Dynamic Wetland System. Ecological Applications. 2009;19(5):1288 - 99.

35. Rees M, Roe JH, Georges A. Life in the Suburbs: Behavior and Survival of A Freshwater Turtle in Response to Drought and Urbanization. Biological Conservation. 2009;142:3172 - 81.

36. Palmer-Allen M, Beynon F, Georges A. Hatching Sex Ratios Are Independent of Temperature in Field Nests of the Long-Necked Turtle, Chelodina longicollis (Testudinata: Chelidae). Wildlife Research. 1991;18:225 - 31.

37. Rhodin AGJ, Ibarrondo BR, Kuchling G. *Chelodina mccordi* Rhodin 1994 - Roti Island Snake-Necked Turtle, McCord's Snake-Necked Turtle, Kura-Kura Rote. In: Rhodin AGJ, Pritchard PCH, van Dijk PP, Saumure RA, Buhlmann KA, Iverson JB, editors. Conservation Biology of Freshwater Turtles and Tortoises: A Compilation Project of the IUCN/SSC Tortoise and Freshwater Turtle Specialist Group. 5: Chelonian Research Monographs; 2008. p. 008.1 - .8.

38. Rhodin AGJ. Chelid turtles of the Australasian Archipelago: II. A new species of *Chelodina* from Roti Island, Indonesia. Breviora. 1994;498:1 - 31.

39. Fordham DA, Georges A, Brook BW. Demographic Response of Snake - Necked Turtles Correlates with Indigenous Harvest and Feral Pig Predation in Tropical Northern Australia. Journal of Animal Ecology. 2007;76:1231 - 43.

40. Mondolfi E. Anotaciones Sobre la Biologia de Tres Quelonios de los Llanos de Venezuela. Memoria De La Sociedad of Cienciass Naturales La Salle. 1955;42:177 - 83.

41. Iverson JB, Higgins H, Sirulink A, Griffiths C. Local and Geographic Variation in the Reproductive Biology of the Snapping Turtle (*Chelydra serpentina*). Herpetologica. 1997;53(1):96 - 117.

42. Eskew EA, Price SJ, Dorcas ME. Survival and Recruitment of Semi-Aquatic Turtles in An Urbanized Region. Urban Ecosystem. 2010;13:365 - 74.

43. Flaherty KL, Anderson JT, Michael ED. Adult Survivorship and Capture Probability of the Common Snapping Turtle (*Chelydra serpentina*). Journal of Herpetology. 2008;42(1):202 - 5.

44. Congdon JD, Dunham AE, van Loben Sels RC. Demographics of Common Snapping Turtles (*Chelydra serpentina*): Implications for Conservation and Management of Long - Lived Organisms. American Zoologist. 1994;34(3):397 - 408.

45. Iverson JB. Patterns of survivorship in turtles (order Testudines). Canadian Journal of Zoology. 1991;69:385 - 91.

46. Brown GP, Bishop CA, Brooks RJ. Growth Rate, Reproductive Output, and Temperature Selection of Snapping Turtles in Habitats of Different Productivities. Journal of Herpetology. 1994;28(4):405 - 10.

47. Paisley RN, Wetzel JF, Nelson JS, Stetzer C, Hamernick MG, Anderson BP. Survival and Spatial Ecology of the Snapping Turtle on the Upper Mississipi River. The Canadian Field-Naturalist. 2009;123(329 - 337).

48. Galbraith DA, Brooks RJ. Survivorship of Adult Females in a Northern Population of Common Snapping Turtles, *Chelydra serpentina*. Canadian Journal of Zoology. 1987;65(7):1581 - 6.

49. Congdon JD, Breitenbach GL, van Loben Sels RC, Tinkle DW. Reproduction and Nesting Ecology of Snapping Turtles (*Chelydra serpentina*) in Southeastern Michigan. Herpetologica. 1987;43(1):39 - 54.

50. Rashid SMA, Swingland IR, editors. On the Ecology of Some Freshwater Turtles in Bangladesh. Conservation, Restoration, and Management of Tortoises and Turtles - An International Conference; 1997; New York: New York Turtle and Tortoise Society.

51. Das I, Singh S. *Chitra indica* (Gray 1830) - Narrow-Headed Softshell Turtle. In: Rhodin AGJ, Pritchard PCH, van Dijk PP, Saumure RA, Buhlmann KA, Iverson JB, et al., editors. Conservation Biology of Freshwater Turtles and Tortoises: A Compilation Project of the IUCN/SSC Tortoise and Freshwater Turtle Specialist Group. 5: Chelonian Research Monographs; 2009. p. 027.1 - .7.

52. Morjan CL. Variation in Nesting Patterns Affecting Nest Temperatures in Two Populations of Painted Turtles (*Chrysemys picta*) With Temperature-Dependent Sex Determination. Behavioral Ecology and Sociobiology. 2003;53:254 - 61.

53. Cooley CR, Floyd AO, Dolinger A, Tucker PB. Demography and Diet of the Painted Turtle (*Chrysemys picta*) at High-Elevation Sites in Southwestern Colorado. The Southwestern Naturalist. 2003;48(1):47 - 53.

54. Mitchell JC. Female Reproductive Cycle and Life History Attributes in a Virginia Population of PaintedTurtles, *Chrysemys picta*. Journal of Herpetology. 1985;19(2):218 - 26.

55. Mitchell JC. Population Ecology and Life Histories of the Freshwater Turtles Chrysemys picta andSternotherus odoratus in an Urban Lake. Herpetological Monographs. 1988;2:40 - 61.

56. Costanzo JP, Iverson JB, Wright MF, Lee and Jr RE. Cold Hardiness and Overwintering Strategies of Hatchlings in an Assemblage of Northern Turtles. Ecology. 1995;76(6):1772 - 85.

57. Rowe JW. Reproductive Variation and the Egg Size-Clutch Size Trade-Off Within and Among Populations of Painted Turtles (*Chrysemys picta bellii*). Oecologia. 1994;99(1):35 - 44.

58. Iverson JB, Smith GR. Reproductive Ecology of the Painted Turtle (*Chrysemys picta*) in the Nebraska Sandhills and across Its Range. Copeia. 1993;1:1 - 21.

59. Spencer RJ, Janzen FJ. Demographic Consequences of Adaptive Growth and the Ramifications For Conservation of Long-Lived Organisms. Biological Conservation. 2010;143:1951 - 9.

60. Tinkle DW, Congdon JD, Rosen PC. Nesting Frequency and Success: Implications for the Demography of Painted Turtles. Ecology. 1981;62(6):1426 - 32.

61. Congdon JD, Tinkle DW. Reproductive Energetics of the Painted Turtle (Chrysemys picta). Herpetologica. 1982;38(1):228 - 37.

62. Rowe JW, Coval KA, Campbell KC. Reproductive Characteristics of Female Midland Painted Turtles (*Chrysemys picta marginata*) From A Population on Beaver Island, Michigan. Copeia. 2003;2003(2):326 - 36.

63. Lindeman PV. Comparative Life History of Painted Turtles (*Chrysemys picta*) in Two Habitats in the Inland Pacific Northwest. Copeia. 1996;1996(1):114 - 30.

64. Flores-Villela OA, Zug GR. Reproductive Biology of the Chopontil, *Claudius angustatu*s (Testudines: Kinosternidae), in Southern Veracruz, Mexico. Chelonian Conservation and Biology. 1995;1(3):181 - 6.

65. Litzgus JD, Mousseau TA. Geographic Variation in Reproduction in A Freshwater Turtle (*Clemmys guttata*). Herpetologica. 2006;62(2):132 - 40.

66. Ernst CH, Zug GR. Observations on the Reproductive Biology of the Spotted Turtle, Clemmys guttata, in Southeastern Pennsylvania. Journal of Herpetology. 1994;28(1):99 - 102.

67. Litzgus JD, Brooks RJ. Reproduction in a Northern Population of Clemmys guttata Journal of Herpetology. 1998;32(2):252 - 9.

68. Litzgus JD. Sex Differences in Longevity in the Spotted Turtle (*Clemmys guttata*). Copeia. 2006;2006(2):281 - 8.

69. Chen T, Lue K. Population Characteristics and Egg Production of The Yellow - Margined Box Turtle, *Cuora flavomarginata flavomarginata*, in Northern Taiwan. Herpetologica. 1999;55(4):487 - 98.

70. Ji-Chao W, Shi-Ping G, Hai-Tao S, Yu-Xiang L, Er-Mi Z. Reproduction and Nesting of the Endangered Keeled Box Turtle (*Cuora mouhottii*) on Hainan Island, China. Chelonian Conservation and Biology. 2011;10(2):159 - 64.

71. Broadley DG, Sachsse W. *Cyclodernma frenatum* Peters 1854 - Zambezi Flapshell Turtle, Nkhasi. In: Rhodin AGJ, Pritchard PCH, van Dijk PP, Saumure RA, Buhlmann KA, Iverson JB, et al., editors. Conservation Biology of Freshwater Turtles and Tortoises: A Compilation Project of the IUCN/SSC Tortoise and Freshwater Turtle Specialist Group. 5: Chelonian Research Monographs; 2011. p. 055.1 - .5.

72. Jackson DR. Reproductive Strategies of Sympatric Freshwater Emydid Turtles in Northern Peninsular Florida. Bulletin of the Florida State Museum, Biological Sciences. 1988;33:113 - 58.

73. Buhlmann KA, Congdon JD, Gibbons JW, Greene JL. Ecology of Chicken Turtles (*Deirochelys reticularia*) in A Seasonal Wetland Ecosystem: Exploiting Resources and Refuge Environments. Herpetologica. 2009;65(1):39 - 53.

74. Gibbons JW. Ecology and Population Dynamics of the Chicken Turtle, *Deirochelys reticularia*. Copeia. 1969;1969(4):669 - 76.

75. Vogt RC, Polisar JR, Moll D, Gonzalez-Porter G. *Dermatemys mawii* Gray 1847 - Central American River Turtle, Tortuga Blanca, Hickatee. In: Rhodin AGJ, Pritchard PCH, van Dijk PP, Saumure RA, Buhlmann KA, Iverson JB, et al., editors. Conservation Biology of Freshwater Turtles and Tortoises: A Compilation Project of the IUCN/SSC Tortoise and Freshwater Turtle Specialist Group. 5: Chelonian Research Monographs; 2011. p. 058.1 - .12.

76. Eiby YA, Booth DT. Determining Optimal Incubation Temperature for a Head-Start Program: the Effect of Incubation Temperature on Hatchling Burnett River Snapping Turtles (*Elseya albagula*). Australian Journal of Zoology. 2011;59:18 - 25.

77. Thomson S, Georges A, Limpus CJ. A New Species of Freshwater Turtle in the Genus *Elseya* (Testudines: Chelidae) from Central Coastal Queensland, Australia. Chelonian Conservation and Biology. 2006;5(1):74 - 86.

78. Georges A, Guarino F, Bito B. Freshwater Turtles of the Transfly Region of Papua New Guineea - Notes on Diversity, Distribution, Reproduction, Harvest, and Trade. Wildlife Research. 2006;33:373 - 84.

79. Freeman A, Thomson S, Cann J. *Elseya lavarackorum* (White and Archer 1994) - Gulf Snapping Turtle, Gulf Snapper, Riversleigh Snapping Turtle, Lavarack's Turtle. In: Rhodin AGJ, Pritchard PCH, van Dijk PP, Saumure RA, Buhlmann KA, Iverson JB, et al., editors. Conservation Biology of Freshwater Turtles and Tortoises: A Compilation Project of the IUCN/SSC Tortoise and Freshwater Turtle Specialist Group. 5: Chelonian Research Monographs; 2014. p. 082.1 - .10.

80. Micheli-Campbell M, Baumgartl T, Booth DT, Campbell HA, Connell M, Franklin CE. Selectivity and Repreated Use of Nesting Sites in A Freshwater Turtle. Herpetologica. 2013;69(4):383 - 96.

81. Berry JF, Shine R. Sexual size dimorphism and sexual selection in turtles (Order Testudines). Oecologia. 1979;42:185 - 91.

82. Rowe JW. Observations of Body Size, Growth, and Reproduction in Blanding's Turtle (*Emydoidea blandingii*) from Western Nebraaska. Canadian Journal of Zoology. 1992;70(9):1690 - 5.

83. Ruane S, Dinkelacker SA, Iverson JB. Demographic and Reproductive Traits of Blanding's Turtles, *Emydoidea blandingii*, at the Western Edge of the Species' Range. Copeia. 2008;2008(4):771 - 9.

84. Congdon JD, Dunham AE, van Loben Sels RC. Delayed Sexual Maturity and Demographics of Blanding's Turtles (*Emydoidea blandingii*): Implications for Conservation and Management of Long - Lived Organisms. Conservation Biology. 1993;7(4):826 - 33.

85. Congdon JD, Nagle RD, Osentoski MF, Kinney OM, van Loben Sels RC. Life History and Demographic Aspects of Aging in the Long - Lived Turtle (*Emydoidea blandingii*). In: Finch CE, Robine J-M, Christen Y, editors. Brain and Longevity. Germany: Springer - Verlag; 2003.

86. Congdon JD, Tinkle DW, Breitenbach GL, van Loben Sels RC. Nesting Ecology and Hatching Success in the Turtle *Emydoidea blandingi*. Herpetologica. 1983;39(4):417 - 29.

87. Standing KL, Herman TB, Morrison IP. Nesting Ecology of Blanding's Turtle (*Emydoidea blandingii*) in Nova Scotia, the Northeastern Limit of the Species's Range. Canadian Journal of Zoology. 1999;77(10):1609 - 14.

88. Trembath DF. The comparative ecology of Krefft’s River Turtle *Emydura krefftii* in Tropical North Queensland. Australia: University of Canberra; 2005.

89. Blamires SJ, Spencer RJ, King P, Thompson MB. Population Parameters and Life - Table Analysis of Two Coexisting Freshwater Turtles: Are the Bellinger River Turtle Populations Threatened? Wildlife Research. 2005;32(4):339 - 47.

90. Gaikhorst GS, Clarke BR, McPharlin M, Larkin B, McLaughlin J, Mayes J. The Captive Husbandry and Reproduction of the Pink-Eared Turtle (*Emydura victoriae*) at Perth Zoo. Zoo Biology. 2011;30:79 - 94.

91. Ernst CH, Barbour RW. Turtles of the world. Washington: Smithsonian Institution Press; 1989. xii, 313 p., 16 p. of plates p.

92. Ayaz D, Fritz U, Atatur MK, Mermer A, Cicek K, Afsar M. Aspect of Population Structure of the European Pond Turtle (*Emys orbicularis*) in Lake Yayla, Western Anatolia, Turkey. Journal of Herpetology. 2008;42(3):518 - 22.

93. Zulfi MAL, Celani A, Foschi E, Tripepi S. Reproductive strategies and body shape in the European pond turtle (*Emys orbicularis*) from contrasting habitats in Italy. Journal of Zoology. 2007;271:218 – 24.

94. Novotny M, Danko S, Havas P. Activity Cycle and Reproductive Characteristics of the European Pond Turtle (*Emys orbicularis*) in the Tajba National Reserve, Slovakia. Biologiaa. 2004;59(14):113 - 21.

95. Drobenkov SM. Reproductive Ecology of the Pond Turtle (*Emys orbicularis* L.) in the Northeastern Part of the Species Range. Russian Journal of Ecology. 2000;31(1):49 - 54.

96. Das I, Bhupathy S. *Geoclemys hamiltonii* (Gray 1830) - Spotted Pond Turtle, Black Pond Turtle. In: Rhodin AGJ, Pritchard PCH, van Dijk PP, Saumure RA, Buhlmann KA, Iverson JB, et al., editors. Conservation Biology of Freshwater Turtles and Tortoises: A Compilation Project of the IUCN/SSC Tortoise and Freshwater Turtle Specialist Group. 5: Chelonian Research Monographs; 2010. p. 043.1 - .6.

97. Hunsinger TW. Demography and Life History of A Wood Turtle (*Clemmys insculpta*) Population in the Hudson River Watershed. . Hudson River Foundation, 2002.

98. Tuttle SE, Carroll DM. Ecology and Natural History of the Wood Turtle (*Clemmys insculpta*) in Southern New Hampshire. Chelonian Conservation and Biology. 1997;2(3):447 - 9.

99. Tuttle SE. Ecology and Natural History of the Wood Turtle (*Clemmys insclupta*) in Southern New Hampshire: Anthioch Universtiy New England; 1996.

100. Greaves WF, Litzgus JD. Variation in Life-History Characteristics Among Populations of North American Wood Turtles: A View From the North. Journal of Zoology. 2009;279:298 - 309.

101. Walde AD, Bider JR, Masse D, Saumure RA, Titman RD. Nesting Ecology and Hatching Success of the Wood Turtle, *Glyptemys insculpta*, in Quebec. Herpetological Conservation and Biology. 2007;2(1):49 - 60.

102. Graham TE, Forsberg JE. Clutch Size in Some Maine Turtles. Bulletion of the Maryland Herpetological Society. 1986;22:146 - 8.

103. Pittman SE, King TL, Faurby S, Dorcas ME. Demographic and Genetic Status of An Isolated Population of Bog Turtles (*Glyptemys muhlenbergii*): Implications For Managing Small Populations of Long-Lived Animals. Conservation Genetics. 2011;12:1589 - 601.

104. Ewert MA, Jackson DR. Nesting Ecology of the Alligator Snapping Turtle (*Macroclemys temminckii*) along the Lower Apalachicola River, Florida. Tallahassee, FL: Nongame Wildlife Program Florida Game and Fresh Water Fish Commission, 1994.

105. Lindeman PV. Aspects of the Life History of the Texas Map Turtle (*Graptemys versa*). The American Midland Naturalist. 2005;153(2):378 - 88.

106. Lovich JE, Madrak SV, Drost CA, Monatesti AJ, Casper D, Znari M. Optimal Egg Size in A Suboptimal Environment: Reproductive Ecology of Female Sonora Mud Turtles (*Kinosternon sonoriense*) in Central Arizona, USA. Amphibia - Reptilia. 2012;33:161 - 70.

107. Lovich JE, Godwin JC, McCoy CJ. *Graptemys ernsti* Lovich and McCoy 1992 - Escambia Map Turtle. In: Rhodin AGJ, Pritchard PCH, van Dijk PP, Saumure RA, Buhlmann KA, Iverson JB, et al., editors. Conservation Biology of Freshwater Turtles and Tortoises: A Compilation Project of the IUCN/SSC Tortoise and Freshwater Turtle Specialist Group. 5: Chelonian Research Monographs; 2011. p. 051.1 - .6.

108. Lindeman PV. Growth Curves for Graptemys, with a Comparison to Other Emydid Turtles. American Midland Naturalist. 1999;142:141 - 51.

109. Horne BD, Brauman RJ, Moore MJC, Seigel RA. Reproductive and Nesting Ecolgy of the Yellow-Blotched Map Turtle, *Graptemys flavimaculata*: Implications for Conservation and Management. Copeia. 2003;2003(4):729 - 38.

110. Vogt RC. Natural History of the Map turtles *Graptemys pseudogeographica* and *G. ouachitensis* in Wisconsin. Tulane Studies in Zoology and Botany. 1980;22:17 - 48.

111. White JD, Moll D. Clutch Size and Annual Reproductive Potential of the Turtle Graptemys geographica in a Missouri Stream. Journal of Herpetology. 1991;25(4):493 - 4.

112. Nagle RD, Lutz CL, Pyle AL. Overwintering in the Nest By Hatchling Map Turtles (*Gramptemys geographica*). Canadian Journal of Zoology. 2004;82:1211 - 8.

113. Ryan KM, Lindeman PV. Reproductive Allometry in the Common Map Turtle, *Graptemys geographica*. The American Midland Naturalist. 2007;158(1):49 - 59.

114. Blankenship EL, Butterfield BP, Godwin JC. *Graptemys nigrinoda* Cagle 1954 - Black - Knobbed Map Turtle, Black - Knobbed Sawback. Chelonian Research Monographs. 2008;5.

115. Jones RL. Reproduction and Nesting of the Endangered Ringed Map Turtle, *Graptemys oculifera*, in Mississipi. Chelonian Conservation and Biology. 2006;5(2):195 - 209.

116. Jones RL, Selman W. *Graptemys oculifera* (Baur 1890) - Ringe Map Turtle, Ringe Sawback. In: Rhodin AGJ, Pritchard PCH, van Dijk PP, Saumure RA, Buhlmann KA, Iverson JB, et al., editors. Conservation Biology of Freshwater Turtles and Tortoises: A Compilation Project of the IUCN/SSC Tortoise and Freshwater Turtle Specialist Group. 5: Chelonian Research Monographs; 2009.

117. Lovich JE, Godwin JC, McCoy CJ. *Graptemys pulchra* Baur 1893 - Alabama Map Turtle. In: Rhodin AGJ, Pritchard PCH, van Dijk PP, Saumure RA, Buhlmann KA, Iverson JB, et al., editors. Conservation Biology of Freshwater Turtles and Tortoises: A Compilation Project of the IUCN/SSC Tortoise and Freshwater Turtle Specialist Group. 5: Chelonian Research Monographs; 2014. p. 072.1 - .6.

118. Martins FI, Souza FL. Estimates of Growth of the Atlantic Rain Forest Freshwater Turtle *Hydromedusa maximiliani* (Chelidae). Journal of Herpetology. 2008;42(1):54 - 60.

119. Martins FI, Souza FL. Demographic Parameters of the Neotropical Freshwater Turtle *Hydromedusa maximiliani* (Chelidae). Herpetologica. 2009;65(1):82 - 91.

120. Souza FL, Martins FI. *Hydromedusa maximiliani* (Mikan 1825) - Maximilian's Snake - Necked Turtle, Brazilian Snake - Necked Turtle. Chelonian Research Monographs. 2009;5(261 - 266).

121. Famelli S, Adriano LR, Pinheiro SCP, Souza FL, Bertoluci J. Reproductive Biology of the Freshwater Turtle Hydromedusa maximiliani (Chelidae) from Southeastern Brazil. Chelonian Conservation and Biology. 2014;13(1):81 - 8.

122. Iverson JB, Vogt RC. *Kinosternon acutum* Gray 1831 - Tabasco Mud Turtle, Montera, Chechagua de Monte. In: Rhodin AGJ, Pritchard PCH, van Dijk PP, Saumure RA, Buhlmann KA, Iverson JB, et al., editors. Conservation Biology of Freshwater Turtles and Tortoises: A Compilation Project of the IUCN/SSC Tortoise and Freshwater Turtle Specialist Group. 5: Chelonian Research Monographs; 2011. p. 062.1 - .6.

123. Wilson DS, Mushinsky HR, McCoy ED. Nesting Behavior of the Striped Mud Turlte, *Kinosternon baurii* (Testudines: Kinosternidae). Copeia. 1999;1999(4):958 - 68.

124. Long DR. Clutch Formation in the Turtle, *Kinosternon flavescens* (Testudines: Kinosternidae). The Southwestern Naturalist. 1986;31(1):1 - 8.

125. Iverson JB. Life History and Demography of the Yellow Mud Turtle, *Kinosternon flavescens*. Herpetologica. 1991;47(4):373 - 95.

126. Iverson JB, Barthelmess EL, Smith GR, deRiverea CE. Growth and Reproduction in the Mud Turtle *Kinosternon hirtipes* in Chihuahua, México. Journal of Herpetology. 1991;25(1):64 - 72.

127. Macip-Rios R, Brauer-Robleda P, Zuniga-Vega JJ, Casas-Andreu G. Demography of two populations of the Mexican mud turtle (*Kinosternon integrum*) in central Mexico. Herpetological Journal. 2011;21:235 - 45.

128. Macip-Rios R, Cisneros MLA, Aguilar-Miguel XS, Casas-Andreu G. Population Ecology and Reproduction of the Mexican Mud Turtle (*Kinosternon Integrum*) in Tonatico, Estado De Mexico. Western North American Naturalist. 2009;64(4):501 - 10.

129. Iverson JB. Reproduction in the Mexican Mud Turtle *Kinosternon integrum*. Journal of Herpetology. 1999;33(1):144 - 8.

130. Iverson JB. Reproduction in the Red - Cheeked Mud Turtle (*Kinosternon scorpioides cruentatum*) in Southeastern Mexico and Belize, with Comparisons Across the Species Range. Chelonian Conservation and Biology. 2010;2010(9):2.

131. van Loben Sels RC, Congdon JD, Austin JT. Life History and Ecology of the Sonoran Mud Turtle (*Kinosternon sonoriense*) in Southeastern Arizona: A Prelimenary Report. Chelonian Conservation and Biology. 1997;2(3):339 - 44.

132. Riedle JD, Rosen PC, Kazmaier RT, Holm P, Jones CA. Conservation Status of an Endemic Kinosternid, *Kinosternon sonoriense longifermorale*, in Arizona. Chelonian Conservation and Biology. 2012;11(2):182 - 9.

133. Stone PA. Movements and Demography of the Sonoran Mud Turtle, *Kinosternon sonoriense*. The Southeastern Naturalist. 2001;46(1):41 - 53.

134. Frazer NB, Gibbons JW, Greene JL. Life History and Demography on the Common Mud Turtle Kinosternon suburbrum in South Carolina, USA. Ecology. 1991;72(6):2218 - 31.

135. Wilkinson LR, Gibbons JW. Patterns of Reproductive Allocation: Clutch and Egg Size Variation in Three Freshwater Turtles. Copeia. 2005;2005(4):858 - 79.

136. Riyanto A. Notes on Exploitation, Population Status, Distribution, and Natural History of the Sulawesi Forest Turtle (*Leucocephalon yuwonoi*) in North-Central Sulawesi, Indonesia. Chelonian Conservation and Biology. 2006;5(2):320 - 3.

137. Hagen C, Platt SG, Innis CJ. *Leucocephalon yuwonoi* (Mccord, Iverson, and Boedai 1995) - Sulawesi Forest Turtle, Kura - Kura Sulawesi. In: Rhodin AGJ, Pritchard PCH, van Dijk PP, Saumure RA, Buhlmann KA, Iverson JB, et al., editors. Conservation Biology of Freshwater Turtles and Tortoises: A Compilation Project of the IUCN/SSC Tortoise and Freshwater Turtle Specialist Group. 5: Chelonian Research Monographs; 2009. p. 039.1 - .7.

138. Howey CAF, Dinkelacker SA. Characteristics of a Historically Harvested Alligator Snapping Turtle (*Macrochelys temminckii*) Population. Copeia. 2013;2013(1):58 - 63.

139. Hart KM, McIvor CC. Demography and Ecology of Mangrove Diamondback Terrapins in a Wilderness Area of Everglades National Park, Florida, USA. Copeia. 2008;2008(1):200 - 8.

140. Feinberg JA, Burke RL. Nesting Ecology and Predation of Diamondback Terrapins, Malaclemys terrapin, at Gateway National Recreation Area, New York. Journal of Herpetology. 2003;37(1):517 - 26.

141. Mitro MG. Demography and Viability Analyses of A Diamondback Terrapin Population. Canadian Journal of Zoology. 2003;81:716 - 26.

142. Brophy TR. Allometry and Sexual Dimorphism in the Snail-Eating Turtle *Malayemys macrocephala* from the Chao Phraya River Basin of Central Thailand. Chelonian Conservation and Biology. 2006;5(1):159 - 65.

143. Pewphong R, Kitana J, Kitana N. Effect of Incubation Temperature on the Somatic Development of the Snail - Eating Turtle *Malayemys macrocephala*. Asian Herpetological Research. 2013;4(4):254 - 62.

144. McCormack TEM, Dawson JE, Hendrie DB, Ewert MA, Iverson JB, Hatcher RE, et al. *Mauremys annamensis* (Siebenrock 1903) - Vietnamese Pond Turle, Annam Pond Turtle, Rùa Trung Bộ. In: Rhodin AGJ, Pritchard PCH, van Dijk PP, Saumure RA, Buhlmann KA, Iverson JB, et al., editors. Conservation Biology of Freshwater Turtles and Tortoises: A Compilation Project of the IUCN/SSC Tortoises and Freshwater Turtle Specialist Group. 5: Chelonian Research Monographs; 2014. p. 081.1 - .14.

145. Bertolero A, Oro D. Conservation Diagnosis of Reintroducing Mediterranean Pond Turtles: what is wrong? Animal Conservation. 2009;12:581 - 91.

146. Keller C. Assessment of Reproductive State in the Turtle *Mauremys leprosa*: A Comparison Between Inguinal Palpation and Radiography. Wildlife Research. 1998;25:527 - 31.

147. Cheng Y, Chen T, Yu P, Chi C. Observations on the Female Reproductive Cycles of Captive Asian Yellow Pond Turtles (*Mauremys mutica*) With Radiography and Ultrasonography. Zoo Biology. 2010;29:50 - 8.

148. Artner H. Successful Breeding of the Chinese Red - necked Pond Turtle Mauremys [Chinemys] nigricans (Gray, 1834). Emys. 2009;16(2):4 - 22.

149. Grosse AM, Buhlmann KA, Hagen C. Nesting Behavior of the Red - necked Pond Turtle (Mauremys [Chinemys] nigricans) in Captivity. Turtle Survival Alliance Magazine. 2010.

150. Lovich JE, Yasukawa Y, Ota H. *Mauremys reevesii* (Gray 1831) - Reeves' Turtle, Chinese Three-Keeled Pond Turtle. In: Rhodin AGJ, Pritchard PCH, van Dijk PP, Saumure RA, Buhlmann KA, Iverson JB, et al., editors. Conservation Biology of Freshwater Turtles and Tortoises: A Compilation Project of the IUCN/SSC Tortoise and Freshwater Turtle Specialist Group. 5: Chelonian Research Monographs; 2011. p. 050.1 - .10.

151. Chen T, Lue K. Ecology of the Chinese Stripe-Necked Turtle, *Ocadia sinensis* (Testudines: Emydidae), in the Keelung River, Northern Taiwan. Copeia. 1998;1998(4):944 - 52.

152. Das I. *Melanochelys tricarinata* (Blyth 1856) - Tricarinate Hill Turtle, Three - Keeled Land Turtle. In: Rhodin AGJ, Pritchard PCH, van Dijk PP, Saumure RA, Buhlmann KA, Iverson JB, et al., editors. Conservation Biology of Freshwater Turtles and Tortoises: A Compilation Project of the IUCN/SSC Tortoise and Freshwater Turtle Specialist Group. 5: Chelonian Research Monographs; 2009. p. 025.1 - .5.

153. Bonin F, Devaux B, Dupré A. Turtles of the world. Baltimore: Johns Hopkins University Press; 2006. 416 p. p.

154. Bohm S. Ecology of the Chelid Turtles *Platemys platycephala*, *Mesoclemmys gibba* and *Mesoclemmys nasuta* in French Guyana. With Notes on Short Term Migrations and Dietary Spectrum of *Platemys platycephala* in the Nouragues Field Reserve, French Guyana. Wien, Austria: Universitat Wien; 2010.

155. Das I. *Morenia ocellata* (Dumeril and Bibron 1835) - Burmese Eyed Turtle. In: Rhodin AGJ, Pritchard PCH, van Dijk PP, Saumure RA, Buhlmann KA, Iverson JB, et al., editors. Conservation Biology of Freshwater Turtles and Tortoises: A Compilation Project of the IUCN/SSC Tortoise and Freshwater Turtle Specialist Group. 5: Chelonian Research Monographs; 2010. p. 044.1 - .5.

156. Blamires SJ, Spencer RJ. Influence of Habitat and Predation on Population Dynamics of the Freshwater Turtle *Myuchelys georgesi*. Herpetologica. 2013;69(1):46 - 57.

157. Rao RJ. Notes on the Sexual Cycle of Female *Trionyx gangeticus* in Central India. Journal of Herpetology. 1986;20(3):455 - 7.

158. Baruah C, Sarma PK, Sharma DK. Status and Conservation of Assam Roofed Turtle Pangshura sylhetensis in the Brahmaputra Floodplain, Assam, India. NeBio. 2010;1(3).

159. Minton JSA. A Contribution to the Herpetology of West Pakistan. Bulletin of the American Museum of Natural History. 1966;`134(2):29 - 178.

160. Auffenberg W, Khan NA. Studies of Pakistan Reptiles Notes on *Kachuga smithi*. Hamadryad. 1991;16(1):25 - 9.

161. Das I, Sengupta S, Praschag P. *Pangshura sylhetensis* Jerdon 1870 - Assam Roofed Turtle. In: Rhodin AGJ, Pritchard PCH, van Dijk PP, Saumure RA, Buhlmann KA, Iverson JB, et al., editors. Conservation Biology of Freshwater Turtles and Tortoises: A Compilation Project of the IUCN/SSC Tortoise and Freshwater Turtle Specialist Group. 5: Chelonian Research Monographs; 2010. p. 046.1 - .6.

162. Ernst CH, Altenburg RGM, Barbour RW. Turtles of the World 2006 [cited 2015 January 2]. Available from: <http://wbd.etibioinformatics.nl/bis/turtles.php>.

163. Narain S, Tripathi A, Mishra SB. Population Ecology of A Freshwater Turtle *Kachuga tentoria* Near Panchnada (Etawah: U.P.) and Its Role as Water Purifier. Journal of Environmental Biology. 2006;27(3):589 - 96.

164. Das I. *Pelochelys cantorii* Gray 1864 - Asian Giant Softshell Turtle. In: Rhodin AGJ, Pritchard PCH, van Dijk PP, Saumure RA, Buhlmann KA, Iverson JB, editors. Conservation Biology of Freshwater Turtles and Tortoises: A Compilation Project of the IUCN/SSC Tortoise and Freshwater Turtle Specialist Group. 5: Chelonian Research Monographs; 2008. p. 011.1 - .6.

165. Bour R. *Pelusios adansonii* (Schweigger 1812) - Adanson's Mud Turtle. In: Rhodin AGJ, Pritchard PCH, van Dijk PP, Saumure RA, Buhlmann KA, Iverson JB, editors. Conservation Biology of Freshwater Turtles and Tortoises: A Compilation Project of the IUCN/SSC Tortoise and Freshwwater Turtle Specialist Group. 5: Chelonian Research Monographs; 2008. p. 017.1 - .4.

166. Gerlach J. *Pelusios castanoides intergularis* Bour 1983 - Seychelles Yellow-Bellied Mud Turtle, Seychelles Chestnut Bellied Terrapin. In: Rhodin AGJ, Pritchard PCH, van Dijk PP, Saumure RA, Buhlmann KA, Iverson JB, editors. Conservation Biology of Freshwater Turtles and Tortoises: A Compilation Project of the IUCN/SSC Tortoise and Freshwater Turtle Specialist Group. 5: Chelonian Research Monographs; 2008. p. 010.1 - .4.

167. Gerlach J. Fragmentation and Demography as Causes of Population Decline in Seychelles Freshwater Turtles (Genus Pelusios). Chelonian Conservation and Biology. 2008;7(1):78 - 87.

168. Broadley DG, Boycott RC. *Pelusios rhodesianus* Hewitt 1927 - Variable Mud Turtle, Variable Hinged Terrapin. In: Rhodin AGJ, Pritchard PCH, van Dijk PP, Saumure RA, Buhlmann KA, Iverson JB, editors. Conservation Biology of Freshwater Turtles and Tortoises: A Compilation Project of the IUCN/SSC Tortoise and Freshwater Turtle Specialist Group. 5: Chelonian Research Monographs; 2008. p. 004.1 - .3.

169. Broadley DG, Boycott RC. *Pelusios sinuatus* (Smith 1838) - Serrated Hinged Terrapin. In: Rhodin AGJ, Pritchard PCH, van Dijk PP, Saumure RA, Buhlmann KA, Iverson JB, et al., editors. Conservation Biology of Freshwater Turtles and Tortoises: A Compilation Project of the IUCN/SSC Tortoise and Freshwater Turtle Specialist Group. 5: Chelonian Research Monographs; 2009. p. 036.1 - .5.

170. Gerlach J. *Pelusios subniger parietalis* Bour 1983 - Seychelles Black Mud Turtle. In: Rhodin AGJ, Pritchard PCH, van Dijk PP, Saumure RA, Buhlmann KA, Iverson JB, editors. Conservation Biology of Freshwater Turtles and Tortoises: A Compilation Project of the IUCN/SSC Tortoise and Freshwater Turtle Specialist Group. 5: Chelonian Research Monographs; 2008. p. 016.1 - .4.

171. Junior PDF, Balestra RAM, Morelra JR, Freitas FO, Lustosa APG, Jorge RF, et al. Nesting of *Phrynops geoffoanus* (Testudines: Chelidae) on Sandy Beaches Along the Upper Xingu River, Brazil. Zoologia. 2011;28(5):571 - 6.

172. Schneider L, Ferrara CR, Vogt RC, Guilhon AV. Nesting Ecology and Nest Predation of *Phrynops geoffroanus* (Testudines, Chelidae) in the Guapore´ River of the Brazilian and Bolivian Amazon. Chelonian Conservation and Biology. 2011;10(2):206 - 12.

173. Bujes C, Verrastro L. Nest Temperature, Incubation Time, Hatchling, and Emergence in The Hilaire's Side - Necked Turtle (*Phrynops hilarii*). Herpetological Conservation and Biology. 2009;4(3):306 - 12.

174. Sung YH, Hau BCH, Karraker NE. Reproduction of the endangered Big-headed Turtle, *Platysternon megacephalum* (Reptilia: Testudines: Platysternidae). Acta Herpetologica. 2014;9(2):243 - 7.

175. Sung YH, Hau BCH, Lau MWN, Crow PA, Kendrick RC, Buhlmann KA, et al. Growth Rate and Evaluation of Age Estimation for the Endangered Bih - Headed Turtle (*Platysternon megacephalum*) in China. Journal of Herpetology. 2015;49(1):99 - 103.

176. Batistella AM, Vogt RC. Nesting Ecology of Podocnemis erythrocephala (Testudines, Podocnemididae) of the Rio Negro, Amazonas, Brazil. Chelonian Conservation and Biology. 2008;7(1):12 - 20.

177. Mogollones SC, Rodriguesz DJ, Hernandez O, Barreto G. A Demographic Study of the Arrau Turtle (*Podocnemis expansa*) in the Middle Orinoco River, Venezuela. Chelonian Conservation and Biology. 2010;9(1):78 - 89.

178. Valuenza N. Maternal Effects of Life-History Traits in the Amazonian Giant River Turtle *Podocnemis expansa*. Journal of Herpetology. 2001;35(3):368 - 78.

179. Alho CJR, Padua LFM. Reproductive Parameters and Nesting Behavior of the Amazon Turtle *Podocnemis expansa* (Testudinata: Pelomedusidae) in Brazil. Canadian Journal of Zoology. 1982;60(1):97 - 103.

180. Portelinha TCG, Malvasio A, Pina CI, Bertoluci J. Reproductive Allometry of *Podocnemis expansa* (Testudines: Podocnemididae) in Southern Brazilian Amazon. Journal of Herpetology. 2013;47(2):232 - 6.

181. Junior PDF, Castro PTA. Nesting Ecology of *Podocnemis expansa* (Schweigger, 1812) and *Podocnemis unifilis* (Troschel, 1848) (Testudines, Podocnemididae) in the Javaes River, Brazil. Brazilian Journal of Biology. 2010;70(1):85 - 94.

182. Alves - Junior JRF, Lustosa APG, Bossi ACS, Balestra RAM, Bastos LF, Miranda LB, et al. Reproductive Indices in Natural Nests of Giant Amazon River Turtles *Podocnemis expansa* (Schweigger, 1812) (Testudines, Podocnemididae) in the Environmental Protection Area Meanders of the Araguaia River. Brazilian Journal of Biology. 2012;72(1).

183. Bonach K, Pina CI, Verdade LM. Allometry of Reproduction of *Podocnemis expansa* in Southern Amazon Basin. Amphibia - Reptilia. 2006;27:55 - 61.

184. Paez VP, Correa JC, Cano AM, Bock BC. A Comparison of Maternal and Temperature Effects on Sex, Size, and Growth of Hatchlings of the Magdalena River Turtle (*Podocnemis lewyana*) Incubated Under Field and Controlled Laboratory Conditions. Copeia. 2009;2009(4):698 - 704.

185. Corea - H JC, Cano-Castano AM, Paez VP, Restrepo A. Reproductive Ecology of the Magdalena River Turtle (*Podocnemis lewyana*) in the Mompos Depression, Colombia. Chelonian Conservation and Biology. 2010;9(1):70 - 8.

186. Pezzuti JCB, Vogt RC. Nesting Ecology of *Podocnemis sextuberculata* (Testudines, Pelomedusidae) in the Japura River, Amazonas, Brazil. Chelonian Conservation and Biology. 1999;3(3):419 - 24.

187. Arraes DR, Tavares - Dias M. Nesting and Neonates of the Yellow - Spotted River Turtle (Podocnemis unifilis, Podocnemididae) in the Araguari River Basin, Eastern Amazon, Brazil. Acta Amazonica. 2014;2014(44):3.

188. Escalona T, Fa JE. Survival of nests of the terecay turtle (*Podocnemis uniflis*) in the Nichare-Tawadu Rivers, Venezuela. Journal of Zoology. 1998;244:303 - 12.

189. Pignati MT, Fernandes LF, Miorando PS, Ferreira PD, Pezzuti JCB. Effects of the Nesting Environment on Embryonic Development, Sex Ratio, and Hatching Success in *Podocnemis unifilis* (Testudines: Podocnemididae) in An Area of Varzea Floodplain on the Lower Amazon River in Brazil. Copeia. 2013;2013(2):303 - 11.

190. Nelson DH, Langford GJ, Borden JA, Turner WM. Reproductive and Hatchling Ecology of the Alabama Red-Bellied Cooter (*Pseudemys alabamensis*): Implications For Conservation and Management. Chelonian Conservation and Biology. 2009;8(1):66 - 73.

191. Lindeman PV. Diet, Growth, Body Size, and Reproductive Potential of the Texas River Cooter (*Pseudemys texana*) in the South Llano River, Texas. The Southwestern Naturalist. 2007;52(4):586 - 94.

192. Bancroft GT, Godley JS, Gross DT, Nan Rojas N, Sutphen DA, McDiarmid RW. Large - scale Operations Management Test of Use of The White Amur for Control of Problem Aquatic Plants: The Herpetofauna of Lake Conway: Species Account. Vicksburg, Mississipi: U.S. Army Engineer Waterways Experiment Station, 1983.

193. Hrycyshyn GE. Survival Probabilities and Density of Four Sympatric Species of Freshwater Turtles in Florida. USA: University of Florida; 2007.

194. Ghaffari H, Plummer MV, Karami M, Mahroo BS, Ahmadzadeh F, Rodder D. Notes on a Nest and Emergence of Hatchlings of the Euphrates Softshell Turtle (*Rafetus euphraticus*) at the Dez River, Iran. Chelonian Conservation and Biology. 2013;12(2):319 - 23.

195. Biricik M, Turga S. Description of An Euphrates Softshell Turtle (*Rafetus euphraticus*) Nest From the Tigris River (SE Turkey). Salamandra. 2011;47(2):99 - 102.

196. Bin HE, Yuxiang L, Haitao S, Jie Z, Maogui H, Yonggang MA, et al. Captive Breeding of the Four-eyed Turtle (*Sacalia quadriocellata*). Asian Herpetological Research. 2010;1(2):111 - 7.

197. Diesmos AC, Buskirk JR, Schoppe S, Diesmos MLL, Emerson YS, Browm RM. *Siebenrockiella leytensis* (Taylor 1920) - Palawan Forest Turtle, Philippine Forest Turtle. In: Rhodin AGJ, Pritchard PCH, van Dijk PP, Saumure RA, Buhlmann KA, Iverson JB, et al., editors. Conservation Biology of Freshwater Turtles and Tortoises: A Compilation Project of the IUCN/SSC Tortoise and Freshwater Turtle Specialist Group. 5: Chelonian Research Monographs; 2012.

198. Iverson JB. Reproduction in Female Razorback Musk Turtles (*Sternotherus carinatus*: Kinosternidae). The Southwestern Naturalist. 1992;47(2):215 - 24.

199. Dodd J, C. K. *Sternotherus depressus* Tinkle and Webb 1955 - Flattened Musk Turtle. In: Rhodin AGJ, Pritchard PCH, van Dijk PP, Saumure RA, Buhlmann KA, Iverson JB, editors. Conservation Biology of Freshwater Turtles and Tortoises: A Compilation Project of the IUCN/SSC Tortoise and Freshwater Turtle Specialist Group. 5: Chelonian Research Monographs; 2008. p. 013.1 - .7.

200. Attum O, Cutshall CD, Eberly K, Day H, Tietjen B. Is There Really No Place Like Home? Movement, Site Fidelity, and Survival Probability of Translocated and Resident Turtles. Biodiversity Conservation. 2013;22:3185 - 95.

201. Bellau P. Habitat Selection, Movement Patterns, and Demography of Common Musk Turtles (*Sternotherus odoratus*) in Southwestern Quebec. Montreal: McGill University; 2008.

202. Dodd J, C. K.,, Ozgul A, Oli MK. The Influence of Disturbance Events on Survival and Dispersal Rates of Florida Box Turtles. Ecological Applications. 2006;16(5):1936 - 44.

203. Dodd J, C. K. Clutch Size and Frequency in Florida Box Turtles (*Terrapene carolina bauri*): Implications for Conservation. Chelonian Conservation and Biology. 1997;2(3):370 - 7.

204. Buchman AB, Cureton II JC, Lutterschmidt WI, Wilson ED. Seasonal Occurrence of Activity and Reproduction of the Three - Toed Box Turtle (*Terrapene carolina triunguis*) in East Texas. Bios. 2010;81(3):84 - 90.

205. Clair RCS. Patterns of Growth and Sexual Size Dimorphism in Two Species of Box Turtles with Environmental Sex Determination. Oecologia. 1998;115:501 - 7.

206. Nazdrowicz NH, Bowman JL, Roth RR. Population Ecology of the Eastern Box Turtle in a Fragmented Landscape. The Journal of Wildlife Management. 2008;72(3):745 - 53.

207. Schwartz ER, Schwartz CW, Kiester AR. The Three - Toed Box Turtle in Central Missouri Part II: a nineteen - year study of home range, movements and population. Missouri Department of Consevation Terrestrial Series. 1984;12.

208. Currylow AF, Zollner PA, Macgowwan BJ, Willliams RN. A Survival Estimate of Midwestern Adult Eastern Box Turtles Using Radiotelemetry. The American Midland Naturalist. 2011;165:143 - 9.

209. Burke RL, Capitano W. Nesting Ecology and Hatching Success of the Eastern Box Turtle, Terrapene carolina, on Long Island, New York. American Midland Naturalist. 2011;165:137 - 42.

210. Milstead WW, Tinkle DW. *Terrapene* of Western Mexico, with Comments on the Species Groups in the Genus. Copeia. 1967;1967(1):180 - 7.

211. Milstead WW. Studies on the Evolution of Box Turtles (Genus *Terrapene*). Bulletion of the Florida State Museum. 1969;14(1):1 - 113.

212. Germano DJ. Activity, Growth, Reproduction, and Population Structure of Desert Box Turtles (*Terrapene ornata luteola*) at the Northern Edge of the Chihuahuan Desert. Chelonian Conservation and Biology. 2014;13(1):56 - 64.

213. Nieuwolt-Dacanay PM. Reproduction in the Western Box Turtle, *Terrapene ornata luteola*. Copeia. 1997;1997(4):819 - 26.

214. Converse SJ, Iverson JB, Savidge JA. Activity, Reproduction and Overwintering Behavior of Ornate Box Turtles (Terrapene ornata ornata) in the Nebraska Sandhills. The American Midland Naturalist. 2003;148(2):416 - 22.

215. Converse SJ, Iverson JB, Savidge JA. Demographics of an Ornate Box Turle Population Experiencing Minimal Human - Induced Disturbances. Ecological Applications. 2005;15(6):2171 - 9.

216. Bowen KD, Colbert PL, Janzen FJ. Survival and Recruitment in a Human - Impacted Population of Ornate Box Turtle, *Terrapene ornat*a, with Recommendation for Conservation and Management. Journal of Herpetology. 2004;38(4):562 - 8.

217. Doroff AM, Keith LB. Demography and Ecology of an Ornate Box Turtle (Terrapene ornata) Population in South-Central Wisconsin. Copeia. 1990;1990(2):387 - 99.

218. Quinn HR, Quinn H, Higa A. Notes on Reproduction and Growth of South Dakota Ornate Box Turtles (*Terrapene ornata*). Chelonian Conservation and Biology. 2014;13(1):65 - 71.

219. Bock BC, Paez VP, Daza JM. *Trachemys callirostris* (Gray 1856) - Colombian Slider, Jicotea, Hicotea, Galapago, Morrocoy de Agua. Chelonian Research Monographs. 2010;5.

220. Bager A, De Freitas TRO, Krause L. Nesting Ecology of a Population of *Trachemys dorbignyi* (Emydidae) in Southern Brazil. Herpetologica. 2007;63(1):56 - 65.

221. Fagundes CK, Bager A, Cechin STZ. *Trachemys dorbigni* in an Anthropic Environment in Southern Brazil: II) Reproductive Ecology. Herpetological Journal. 2010;20:195 - 9.

222. Morjan CL, Stuart JN. Nesting Record of a Big Bend Slider Turtle (*Trachemys gaigeae*) in New Mexico, and Overwintering of Hatchlings in the Nest. The Southwestern Naturalist. 2001;46(2):230 - 4.

223. Stuart JN, Ward JP. *Trachemys gaigeae* (Hartweg 1939) - Big Bend Slider, Mexican Plateau Slider, Jicotea de la Meseta Mexicana. In: Rhodin AGJ, Pritchard PCH, van Dijk PP, Saumure RA, Buhlmann KA, Iverson JB, et al., editors. Conservation Biology of Freshwater Turtles and Tortoises: A Compilation Project of the IUCN/SSC Tortoise and Freshwater Turtle Specialist Group. 5: Chelonian Research Monographs; 2009. p. 032.1 - .12.

224. Frazer NB, Gibbons JW, Greene JL. Life Tables of a Slider Turtle Population. In: Gibbons JW, editor. Life History and Ecology of the Slider Turtle. Washington, D. C.: Smithsonian Institution Press; 1990.

225. Frazer NB, Gibbons JW, Greene JL. Exploring Fabens' Growth Interval Model with Data on a Long-Lived Vertebrate, *Trachemys scripta* (Reptilia: Testudinata). Copeia. 1990;1990(1):112 - 8.

226. Gibbons JW, Semlitsch RD, Greene JL, Schibauer JP. Variation in Age and Size at Maturity of the Slider Turtle (*Pseudemys scripta*). The American Naturalist. 1981;117(5):841 - 5.

227. Hays KA, McBee K. Population Demographics of Red - Eared Slider Turtles (*Trachemys scripta*) From Tar Creek Superfund Site. Journal of Herpetology. 2010;44(3):441 - 6.

228. Tucker JK, Paukstis GL, Janzen FJ. Annual and Local Variation in Reproduction in the Red-Eared Slider, *Trachemys scripta elegans*. Journal of Herpetology. 1998;32(4):515 - 26.

229. Gidis M, Kaska Y. Population Size, Reproductive Ecology and Hevay Metal Levels in Eggshell of the Nile Soft-Shell Turtle (*Trionyx triunguis*) Around Thermal Lake Kukurtlu, Mugla-Turkey (Sulphurous) Fresenius Environmental Bulletin. 2004;13(5):405 - 12.

230. Whitaker N, Vijaya J. Biology of the Forest Cane Turtle, *Vijayachelys silvatica*, in South India. Chelonian Conservation and Biology. 2009;8(2):109 - 15.

231. Moll EO, Groombridge B, Vijaya J. Redescription of the Cane Turtle With Notes on Its Natural History and Classification. Journal Bombay Natural History Society 1986;83:112 - 26.
