# Supplementary material for: Population dynamics and biological feasibility of sustainable harvesting as a conservation strategy for tropical and temperate freshwater turtles
Source: PLoS One. 2020 Feb 27;15(2):e0229689. doi: 10.1371/journal.pone.0229689 (PMC7046234; doi:10.1371/journal.pone.0229689)
Supplement: S1 File — (DOCX) [file pone.0229689.s002.docx]

Carapace length correlations

Correlations between explanatory variables were examined prior to running the Generalized Additive Models (GAMs).

|  | Untransformed | Transformed |
| --- | --- | --- |
|  | 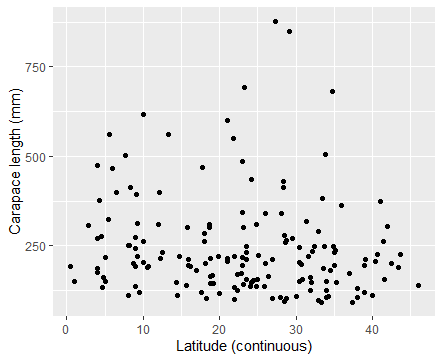 | 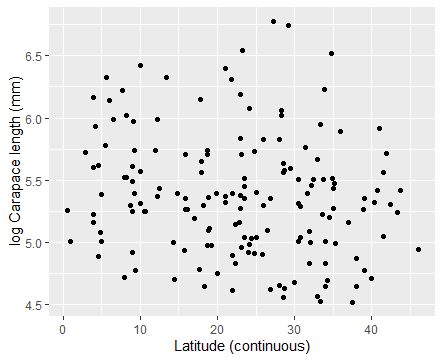 |
|  | 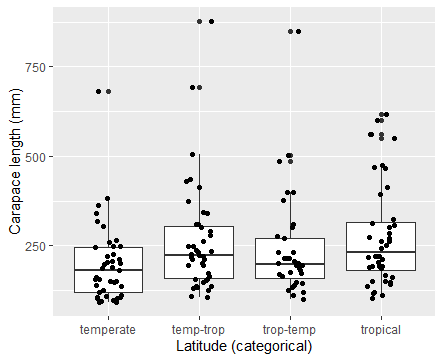 | 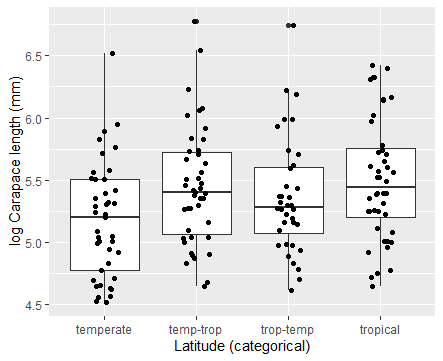 |
|  | 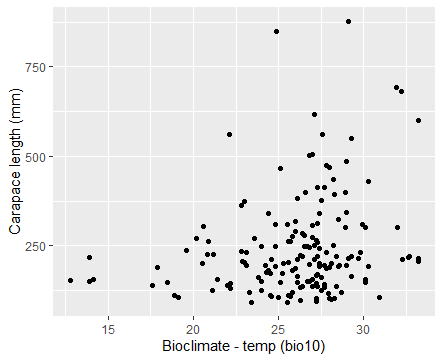 | 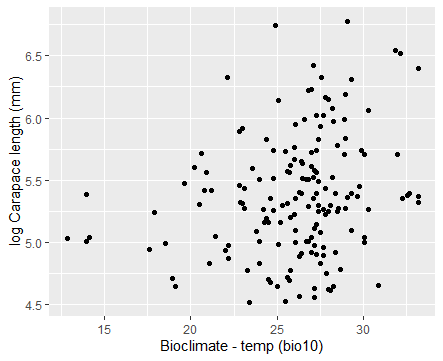 |
|  | 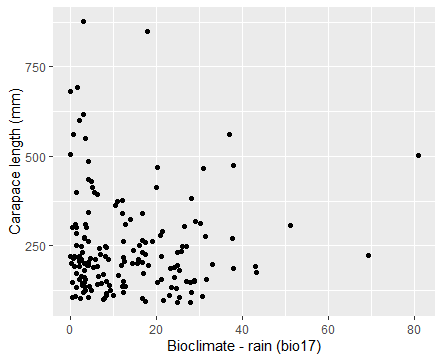 | 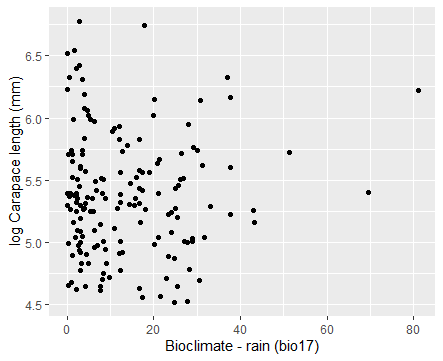 |
|  |  |  |

Table 1. Correlation between explanatory variables.

|  | Carapace length | log Carapace length |
| --- | --- | --- |
| Continuous latitude^a^ | -0.13 | -0.17 |
| Categorical latitude^b^ | 0.19 | 0.23 |
| Bioclimate - temp^a^ | 0.24 | 0.24 |
| Bioclimate - rain^a^ | -0.01 | 0.01 |

^a^ Pearson correlation values

^b^ Polyserial rho values

References

Drasgow, F. (1986) Polychoric and polyserial correlations. Pp. 68–74 in S. Kotz and N. Johnson, eds., The Encyclopedia of Statistics, Volume 7. Wiley.
