# Supplementary material for: Population dynamics and biological feasibility of sustainable harvesting as a conservation strategy for tropical and temperate freshwater turtles
Source: PLoS One. 2020 Feb 27;15(2):e0229689. doi: 10.1371/journal.pone.0229689 (PMC7046234; doi:10.1371/journal.pone.0229689)
Supplement: Attachment — Submitted filename: PONE-D-19-28843_reviewer.pdf [file pone.0229689.s004.pdf]

PLOS ONE

Population Dynamics and Biological Feasibility of Sustainable Harvesting as a Conservation Strategy for Tropical and Temperate Freshwater Turtles

--Manuscript Draft--

**Manuscript Number:**	PONE-D-19-28843
**Article Type:**	Research Article
**Full Title:**	Population Dynamics and Biological Feasibility of Sustainable Harvesting as a Conservation Strategy for Tropical and Temperate Freshwater Turtles
**Short Title:**	Biological Feasibility of Sustainable Harvesting for Freshwater Turtles
**Corresponding Author:**	Darren Norris, Ph.D.<br>Universidade Federal de Amapa<br>Macapa, BRAZIL
**Keywords:**	age; Clutch size; Fecundity; Growth rates; Isotherm; life history; Mortality; survival; Population Dynamics; Population ecology; Reproduction; Reptile; Sexual maturity; Sustainable harvest; Turtles
**Abstract:**	Background: Conservation strategies are urgently needed for tropical turtles. Studies conducted exclusively in the temperate zone have revealed that the suite of life history traits that characterizes turtles and includes delayed sexual maturity and high adult survivorship makes sustainable harvest programs an unviable strategy for turtle conservation. However, most turtles are tropical in distribution and the tropics have higher, more constant and more extended ambient temperature regimes that, in general, are more favorable for population growth.<br>Methods: To estimate the capacity of freshwater turtle species from temperate and tropical regions to sustain harvest we synthesized life history traits from 165 freshwater turtle species in 12 families (Carettochelydae, Chelidae, Chelydridae, Dermatemydidae, Emydidae, Geoemydidae, Kinosternidae, Pelomedusidae, Platysternidae, Podocnemididae, Staurotypidae and Trionychidae). The influence of climate variables and latitude on freshwater turtle life history traits (clutch size, clutch frequency, age at sexual maturity, and annual adult survival) were examined using Generalized Additive Models. The biological feasibility of sustainable harvest in temperate and tropical species was evaluated using a sensitivity analysis of population growth rates obtained from stage structured matrix population models.<br>Results: Turtles at low latitudes (tropical zones) exhibit smaller clutch sizes, higher clutch frequency, and earlier age at sexual maturity than those at high latitudes (temperate zone). Adult survival increased weakly with latitude and declined significantly with increasing bioclimatic temperature (mean temperature of warmest quarter). A modeling synthesis of these data indicates that the interplay of life history traits does not create higher harvest opportunity in adults of tropical species. Yet we found potential for sustainable exploitation of eggs in tropical species.<br>Conclusions: Sustainable harvest as a conservation strategy for tropical turtles appears to be as biologically problematic as in temperature zones and likely only possible if the focus is on limited harvest of eggs. Further studies are urgently needed to understand how the predicted population surplus in early life stages can be most effectively incorporated into conservation programs for tropical turtles increasingly threatened by unsustainable exploitation, climate change and deforestation.
**Order of Authors:**	Angga Rachmansah
	Darren Norris, Ph.D.
	James Peter Gibbs
**Additional Information:**	
**Question**	**Response**
**Financial Disclosure**<br><br>Enter a financial disclosure statement that	The author(s) received no specific funding for this work.

Powered by Editorial Manager® and ProduXion Manager® from Aries Systems Corporation

describes the sources of funding for the work included in this submission. Review the submission guidelines for detailed requirements. View published research articles from *PLOS ONE* for specific examples.<br><br>This statement is required for submission and **will appear in the published article** if the submission is accepted. Please make sure it is accurate.<br><br>**Unfunded studies**<br>Enter: *The author(s) received no specific funding for this work.*<br><br>**Funded studies**<br>Enter a statement with the following details:<br>• Initials of the authors who received each award<br>• Grant numbers awarded to each author<br>• The full name of each funder<br>• URL of each funder website<br>• Did the sponsors or funders play any role in the study design, data collection and analysis, decision to publish, or preparation of the manuscript?<br>• **NO** - Include this sentence at the end of your statement: *The funders had no role in study design, data collection and analysis, decision to publish, or preparation of the manuscript.*<br>• **YES** - Specify the role(s) played.<br><br>* typeset	
**Competing Interests**<br><br>Use the instructions below to enter a competing interest statement for this submission. On behalf of all authors, disclose any competing interests that could be perceived to bias this work—acknowledging all financial support and any other relevant financial or non-financial competing interests.<br><br>This statement **will appear in the published article** if the submission is accepted. Please make sure it is accurate. View published research articles from *PLOS ONE* for specific examples.	The authors have declared that no competing interests exist.

Powered by Editorial Manager® and ProduXion Manager® from Aries Systems Corporation

**NO authors have competing interests**<br><br>Enter: *The authors have declared that no competing interests exist.*<br><br>**Authors with competing interests**<br><br>Enter competing interest details beginning with this statement:<br><br>*I have read the journal's policy and the authors of this manuscript have the following competing interests: [insert competing interests here]*<br><br>* typeset	
**Ethics Statement**<br><br>Enter an ethics statement for this submission. This statement is required if the study involved:<br><br>• Human participants<br>• Human specimens or tissue<br>• Vertebrate animals or cephalopods<br>• Vertebrate embryos or tissues<br>• Field research<br><br>Write "N/A" if the submission does not require an ethics statement.<br><br>General guidance is provided below. Consult the submission guidelines for detailed instructions. **Make sure that all information entered here is included in the Methods section of the manuscript.**	N/A

**Format for specific study types**<br><br>**Human Subject Research (involving human participants and/or tissue)**<br>• Give the name of the institutional review board or ethics committee that approved the study<br>• Include the approval number and/or a statement indicating approval of this research<br>• Indicate the form of consent obtained (written/oral) or the reason that consent was not obtained (e.g. the data were analyzed anonymously)<br><br>**Animal Research (involving vertebrate animals, embryos or tissues)**<br>• Provide the name of the Institutional Animal Care and Use Committee (IACUC) or other relevant ethics board that reviewed the study protocol, and indicate whether they approved this research or granted a formal waiver of ethical approval<br>• Include an approval number if one was obtained<br>• If the study involved *non-human primates*, add *additional details* about animal welfare and steps taken to ameliorate suffering<br>• If anesthesia, euthanasia, or any kind of animal sacrifice is part of the study, include briefly which substances and/or methods were applied<br><br>**Field Research**<br><br>Include the following details if this study involves the collection of plant, animal, or other materials from a natural setting:<br>• Field permit number<br>• Name of the institution or relevant body that granted permission	
**Data Availability**<br><br>Authors are required to make all data underlying the findings described fully available, without restriction, and from the time of publication. PLOS allows rare exceptions to address legal and ethical concerns. See the PLOS Data Policy and FAQ for detailed information.	Yes - all data are fully available without restriction

A Data Availability Statement describing where the data can be found is required at submission. Your answers to this question constitute the Data Availability Statement and **will be published in the article**, if accepted.<br><br>**Important:** Stating 'data available on request from the author' is not sufficient. If your data are only available upon request, select 'No' for the first question and explain your exceptional situation in the text box.<br><br>Do the authors confirm that all data underlying the findings described in their manuscript are fully available without restriction?	
**Describe where the data may be found in full sentences. If you are copying our sample text, replace any instances of XXX with the appropriate details.**<br><br>• If the data are **held or will be held in a public repository**, include URLs, accession numbers or DOIs. If this information will only be available after acceptance, indicate this by ticking the box below. For example: *All XXX files are available from the XXX database (accession number(s) XXX, XXX.)*.<br>• If the data are all contained **within the manuscript and/or Supporting Information files**, enter the following: *All relevant data are within the manuscript and its Supporting Information files.*<br>• If neither of these applies but you are able to provide **details of access elsewhere**, with or without limitations, please do so. For example:<br><br>*Data cannot be shared publicly because of [XXX]. Data are available from the XXX Institutional Data Access / Ethics Committee (contact via XXX) for researchers who meet the criteria for access to confidential data.*<br><br>*The data underlying the results presented in the study are available from (include the name of the third party*	All relevant data are within the manuscript and its Supporting Information files.

and contact information or URL).<br>• This text is appropriate if the data are owned by a third party and authors do not have permission to share the data.<br><br>* typeset	
Additional data availability information:	

Manuscript

Click here to access/download;Manuscript;Rachmansah_manuscript_plosone.

Title: Population Dynamics and Biological Feasibility of Sustainable Harvesting as a Conservation Strategy for Tropical and Temperate Freshwater Turtles

Short title: Biological Feasibility of Sustainable Harvesting for Freshwater Turtles

Angga Rachmansah[1,#a,] , Darren Norris[2,3,*], James P. Gibbs[1]

[1]Department of Environmental and Forest Biology, State University of New York College of Environmental Science and Forestry, Syracuse, New York, United States of America

[2]Ecology and Conservation of Amazonian Vertebrates Research Group, Federal University of Amapá, Rod. Juscelino Kubitschek Km 02, 68903-419 Macapá, Amapá, Brazil

[3]Postgraduate Programme in Tropical Biodiversity, Federal University of Amapá, Rod. Juscelino Kubitschek Km 02, 68903-419 Macapá, Amapá, Brazil

[#a]Current Address: Fauna Flora International – Indonesia Programme, Jakarta, Indonesia

*Corresponding author. Coordenação de Ciências Ambientais, Federal University of Amapá, Rod. Juscelino Kubitschek Km 02, 68903-419 Macapá, Brazil. Email: dnorris75@gmail.com

Keywords: Age; Clutch size; Fecundity; Growth rates; Isotherm; Life history; Mortality; Survival; Population dynamics; Population ecology; Reproduction; Reptile; Sexual maturity; Sustainable harvest; Turtles

Abstract

Background: Conservation strategies are urgently needed for tropical turtles. Studies conducted exclusively in the temperate zone have revealed that the suite of ~~life history traits~~ that characterizes turtles and includes delayed sexual maturity and high adult survivorship makes sustainable harvest programs an unviable strategy for turtle conservation. However, most turtles are tropical in distribution and the tropics have higher, more constant and more extended ambient temperature regimes that, in general, are more favorable for population growth.

Methods: To estimate the capacity of freshwater turtle species from temperate and tropical regions to sustain harvest we synthesized life history traits from 165 freshwater turtle species in 12 families (Carettochelydae, Chelidae, Chelydridae, Dermatemydidae, Emydidae, Geoemydidae, Kinosternidae, Pelomedusidae, Platysternidae, Podocnemididae, Staurotypidae and Trionychidae). The influence of climate variables and latitude on freshwater turtle life history traits (clutch size, clutch frequency, age at sexual maturity, and annual adult survival) were examined using Generalized Additive Models. The biological feasibility of sustainable harvest in temperate and tropical species was evaluated using a sensitivity analysis of population growth rates obtained from stage structured matrix population models.

Results: Turtles at low latitudes (tropical zones) exhibit smaller clutch sizes, higher clutch frequency, and earlier age at sexual maturity than those at high latitudes (temperate zone). Adult survival increased weakly with latitude and declined significantly with increasing bioclimatic temperature (mean temperature of warmest quarter). A modeling synthesis of these data indicates that the interplay of life history traits does not create higher harvest opportunity in adults of tropical species. Yet we found potential for sustainable exploitation of eggs in tropical species.

Conclusions: Sustainable harvest as a conservation strategy for tropical turtles appears to be as biologically problematic as in temperature zones and likely only possible if the focus is on limited harvest of eggs. Further studies are urgently needed to understand how the predicted population surplus in early life stages can be most effectively incorporated into conservation programs for tropical turtles increasingly threatened by unsustainable exploitation, climate change and deforestation.

Introduction

Vertebrate animals are important for human welfare and wellbeing [1-3], particularly as food, medicine, and cultural uses by rural and aboriginal communities [3-6]. Freshwater turtles are a good example — they are frequently targeted for both subsistence and commercial harvest, primarily by local communities that live in the vicinity of river and wetlands [7-9]. High biomass [10, 11], ease of capture, and extended survival with minimal care in captivity make freshwater turtles a focus for harvest [7-9].

Unsustainable harvesting is recognized as one of the major factors driving global freshwater turtle decline [12-15] . Over 40% of turtle species are endangered as a result of overexploitation [13, 15, 16]. Although turtles are harvested for various purposes (e.g. pets, medicine, and curios), the most heavy use of turtles is for food [7, 16, 17]. Large adult turtles [18-21] and eggs [18] are usually the primary target of harvesting, because these life stages are the most valuable for food [7, 8, 16] and the easiest life stages to encounter. The greatest harvesting pressure occurs in tropical areas [7, 8] where the most freshwater turtles occur [22]. For many local people in these areas, turtle meat and eggs are not only important as sources of protein and lipid, but also support them economically [7, 16, 23]. Additionally, unsustainable exploitation in tropical areas can also lead to regional population collapse and as a consequence create pressures in other regions of the world [24].

Sustainable harvesting programs have been widely promoted as a strategy for wildlife conservation [25, 26]. Moreover, active involvement of local people in these sustainable harvest programs generally creates better outcomes for conserving wildlife [26, 27]. However, this conservation strategy is assumed not viable for turtle conservation [7, 28]. A corpus of research

on the topic has revealed that turtles are poor candidates for any sustainable use program [29-31]. In general, turtles exhibit delayed sexual maturity, high adult survivorship, low fecundity, and long life span [29-34]. This combination of life history traits limits their ability to compensate for additive adult mortality from harvesting [9, 28, 32, 34, 35].

It is notable, however, that virtually all research on sustainability of harvest as a conservation strategy for turtles has been conducted in temperate zones. Variation in life history traits occur within and between turtle species that inhabit different environments [32, 36-40]. Variation in clutch size [36, 41], clutch frequency [33], growth rate, and age at sexual maturity [36] in relation to latitude have been observed in turtles. The interplay of these different life history traits has been suggested to create more opportunity to harvest turtles sustainably, at least in one tropical freshwater species in Northern Australia [19, 42]. Earlier age at sexual maturity, higher fecundity, and faster growth rates in this tropical freshwater turtle compared to other turtles [42] may allow their populations to be harvested at 20% annual harvest rate [19], suggesting that the widely held assumption of the biological infeasibility of sustainable harvest programs for freshwater turtles based almost entirely on temperate zone species should be reassessed given the challenges of conserving turtles in rapidly developing tropical regions where most turtle diversity occurs [9, 13].

In this study, we investigated global patterns of life history traits (clutch size, clutch frequency, age at sexual maturity, and adult survival) in freshwater turtles using published data and contrasted them between freshwater turtle species from temperate and tropical regions. We then developed a population projection model to estimate the capacity of freshwater turtle species from temperate and tropical regions to sustain harvest. The primary goal of this study was to evaluate the hypothesis that freshwater turtle species from tropical and temperate regions

have the same, widely speculated incapacity to absorb additive mortality caused by population harvest [29, 30, 35].

Materials and Methods

Data Collection

Life history traits of freshwater turtle species were quantified along with locality of each report (latitude and longitude) from the published literature. We used keywords "life history", "clutch size", "clutch frequency", "reproduction", "age at sexual maturity", "survival", "growth", "natural history", and "turtle" to explore the published literature as indexed in the databases of EBSCO, Google Scholar, and Web of Science. The mean, median, or range values of reproductive parameters (clutch size, clutch frequency), demographic parameters (age at sexual maturity, annual adult survival rate), and morphological characters (carapace length) were extracted from each report acquired. Annual adult survival values were also checked and confirmed against those available for 15 freshwater turtle species in an online demographic database COMADRE [43] (version 3.0.0, accessed 2 September 2019 http://www.comadre-db.org/Data/Comadre).

When the exact coordinates of locality were not described, we estimated location from the nearest locality described in a given report. The coordinates of each turtle life history report were also combined with GBIF records (accessed via GBIF.org on 2019-01-13) and published data [38] to establish species distribution across four latitudinal classes: Temperate (species with latitudinal median and range within temperate zone), Temperate-tropical ("Temp-trop", species with latitudinal median within temperate and range overlapping tropical zone/s), Tropical-

temperate (“Trop-temp”, species with latitudinal median within tropical and range overlapping temperate zone/s), Tropical (species with latitudinal median and range within tropical zone). The tropics of Capricorn and Cancer (latitude -23.5^{o}, 23.5^{o}, respectively) were used to define geographic limits of temperate and tropical zones.

Two bioclimatic variables relevant to freshwater turtle biology, Mean Temperature of Warmest Quarter (bio10, ^{o}C) and Precipitation of Driest Quarter (bio17, mm) were obtained from WorldClim – Global Climate Data (5-arc ≈ 10 km resolution, www.worldclim.org, [44]) and matched to the coordinates of each turtle life history report. These bioclimatic variables were selected as proxies to represent the metabolic, physiological and behavioral differences that freshwater turtles have developed to survive in regions that are not ideal for these temperature and water dependent species [10, 22, 32, 33, 38-41, 45]. Both bioclimatic variables were only weakly correlated with latitude (Spearmans correlation 0.40 and 0.04 for bio10 and bio17 respectively) and were therefore included to represent temperature and rainfall patterns distinct to those most strongly associated with latitudinal gradients.

Statistical Analysis

We used Generalized Additive Models (GAMs, [46, 47]) to examine the influence of climate variables and latitude on freshwater turtle life history traits (clutch size, clutch frequency, age at sexual maturity, and annual adult survival). We treated each freshwater turtle species as a replicate in this analysis (obtaining median life history values within species for species with *n* > 1 reports) to avoid the pitfalls of pseudoreplication associated with treating individual reports as replicates. Because comparative life history studies are not independent from phylogenetic relationships among turtles, which can lead to phylogenetic bias on inference and trait value

estimation, we treated taxonomic family as a random effect (penalized smoothed regression term) [48, 49] based on the Turtles of the World Checklist (8th edition, [50]). In addition, we used carapace length (ln-transformed) as a parametric term to control for its well-established influence on life history traits [33, 36, 38, 41].

A total of four models were developed for each life history trait: latitude as a continuous variable included as a parametric term, latitude as a categorical variable with four classes (Temperate, Temp-Trop, Trop-Temp and Tropical), and two bioclimatic variables (Mean Temperature of Warmest Quarter and Precipitation of Driest Quarter) included as parametric terms. All life history trait estimates were ln-transformed, except for adult survival (arcsine transformed). The mgcv package [46] was used to perform the GAM analysis in R (www.r-project.org, [51]). Akaike Information Criterion corrected for small sample sizes (AICc) that measures fit versus complexity of a model was used to select "best" models based on lowest AICc [52, 53].

Modelling Synthesis

To evaluate whether freshwater turtle species from tropical and temperate zones have comparable capacities to absorb additive mortality caused by population harvest, we implemented a density-independent, stage structured "Lefkovitch" matrix population model [34, 54, 55]. This type of model is commonly used in turtle population dynamics modelling, as age in most turtle species is often difficult to determine [19, 34]. The model consisted of egg, juvenile, and adult stages (Fig 1) projected with a stable stage distribution (with an initial population of 1000, allocated in proportions of 0.544, 0.401, 0.055 to egg, juvenile and adult stages

respectively). The discrete stage based lifecycle (Fig 1) can be presented as a population projection matrix “A” as follows:

$$\boldsymbol{A} = \begin{bmatrix} 0 & 0 & F \\ G_1 & P_1 & 0 \\ 0 & G_2 & P_2 \end{bmatrix}$$

where *P* is the annual probability of surviving and remaining in the same stage, *G* is the annual probability of surviving and growing into next stage, and *F* is the annual fecundity. These parameters were estimated using the following equations [56]:

$$\boldsymbol{P} = \frac{\left(\boldsymbol{1-p_i^{d_i-1}}\right)}{\left(\boldsymbol{1-p_i^{d_i}}\right)}\,\boldsymbol{p_i} \qquad \text{Equation 1}$$

$$\boldsymbol{G} = \frac{\boldsymbol{p_i}^{\boldsymbol{d_i}}\,(\boldsymbol{1-p_i})}{\boldsymbol{1-p_i^{d_i}}} \qquad \text{Equation 2}$$

where p_i is the annual survival probability of *i* stage and d_i is the duration of *i* stage. Annual fecundity (*F*) was estimated by multiplying clutch size with clutch frequency. The model was based on female fraction only; thus half of all eggs produced was assumed to be female [29, 30] . The stable distributions of individuals amongst stage classes, and intrinsic rate of population growth (*r*) were determined with functions available in the R [51] packages “popdemo” [57] and “popbio” [58].

Fig 1. Conceptual diagram of population dynamics of freshwater turtles used for construction of a stage structure matrix model to estimate capacity for sustainable harvest in freshwater turtles.

Median values of clutch size, clutch frequency, age at sexual maturity and adult survival derived from the GAM predictions was used as input for this stage-structured model. Due to the sparsity of records for some traits (e.g. adult survival) predictions were aggregated across two latitudinal classes (temperate and tropical) to compare the intrinsic rate of population growth (*r*) between stages and latitude. Predictions for each trait were obtained from a final model that included all variables in a 95% confidence subset of models [52]. This confidence set was obtained by summing the Akaike weights of the set of all candidate models ordered by Akaike weight from largest to smallest until a sum of ≥0.95 was obtained ([52] pp. 169, 176-177). We estimated the annual survival probability of juvenile stage as 13% less than the annual survival probability of adult stage [59]. Due to lack of available nest / hatchling survival data the annual survival probability of egg stage for all turtle species was set at 0.2 [29, 30, 32]. To simulate the impact of harvest on populations of tropical and temperate freshwater turtle species, we performed a sensitivity analysis by varying each demographic parameter systematically while holding all other parameters constant [29, 30]. In addition, we performed Jackknife randomizations [60] drawing deviates (n = 500 iterations) for each model parameter from the distributions observed in the literature (S1 Table) for these variables to estimate confidence intervals around the estimated intrinsic rates of growth of temperate and tropical species in sensitivity analysis.

Table 1. Demographic parameters in freshwater turtles. Demographic parameters used in population modelling to estimate capacity for sustainable harvest in freshwater turtles. Estimates are median values derived from the scientific literature (S1 Table) and summarized based on the species distributions across four latitudinal classes.

Distribution[a]	Families	Species	Lat[b]	Carapace Length	Clutch Size	Clutch Frequency	Age at sexual maturity	Fecundity
Temperate	6	41	34.0	181.0	8.4	2.0	8.7	7.8
Temp-Trop	6	43	29.1	221.7	11.2	1.7	8.3	6.6
Trop-Temp	10	37	18.3	197.3	6.1	2.5	6.5	6.0
Tropical	10	44	9.6	231.5	7.3	2.0	9.0	3.5
Overall	12	164	23.1	208.0	8.0	2.0	8.3	6.3

[a] Distribution of freshwater turtles in four latitudinal classes: Temperate (species latitudinal median and range within temperate zone), Temp-trop (species latitudinal median within temperate and range overlapping tropical zone/s), Trop-temp (species latitudinal median within tropical and range overlapping temperate zone/s), Tropical (species latitudinal median and range within tropical zone). This classification is unique for each species i.e. a species is only included in one class.

[b] Median latitude from species locations within each distribution class.

Results

A total of 461 reports of life history traits was obtained from 165 species (63% of living freshwater turtle species) among 12 taxonomic families (Fig 2, S1 Table). The data once aggregated represent: 84 species from 7 families in the temperate zone (Temperate and Temperate-Tropical classes) and 81 species from 12 families in the tropical zone (Tropical-Temperate and Tropical classes). Sixty percent of these studies were from temperate areas, with most of these (73%) from North America (Fig 2). Forty percent of these data were from tropical areas, with most of these (36%) from Asia. Only 12 of ~~these life history trait reports~~ were from captive breeding situations while the remainder were from wild populations.

Fig 2. Distribution of freshwater turtle studies. Geographic distribution of data on freshwater turtle life history traits obtained from the literature (S1 Table) to estimate capacity for sustainable harvest in freshwater turtles. Color of study locations represent the distribution of the study

species across four latitudinal classes: Temperate (species with latitudinal median and range within temperate zone), "Temp-Trop" (species with latitudinal median within temperate and range overlapping tropical zone/s), "Trop-Temp" (species with latitudinal median within tropical and range overlapping temperate zone/s), Tropical (species with latitudinal median and range within tropical zone). Dashed horizontal lines show Tropic of Cancer and Tropic of Capricorn (latitude -23.5^{o}, 23.5^{o}, respectively). The background map was obtained from the 1:110m Natural Earth country and geographic lines maps (http://www.naturalearthdata.com).

Latitude as continuous variable significantly influenced all life history traits, except adult survival (Table 2, Table 3). Indeed, latitude was the most informative variable for clutch size, clutch frequency and age at sexual maturity (Table 3). Natural logarithm of clutch size (β = 0.13; P < 0.001) and age at sexual maturity (β = 0.06; P < 0.01) were positively related to latitude, whereas natural logarithm of clutch frequency (β = -0.09; P < 0.05) exhibited a negative relationship with latitude (Fig 1, Table 2). When latitude was treated as categorical variable, only the natural logarithm of clutch size was significantly related to latitudinal zones, such that Tropical (β = -0.21; P < 0.001) and Tropical-Temperate (β = -0.13; P < 0.05) species had reduced clutch size relative to temperate species (Table 2).

Fig 3. Relationships between latitude and (A) clutch size, (B) clutch frequency, (C) age at sexual maturity, and (D) adult survival rate of freshwater turtles. Points are the median species values obtained from the literature (S1Table), colored representing ln carapace length values. Solid black line is the GAM prediction. Grey shaded polygons show 95% confidence bands around the prediction.

Of the two bioclimatic variables assessed, only bioclimatic temperature (Mean Temperature of Warmest Quarter) was a contributor to life history variation (Table 2) and was also the most informative variable for adult survival (Table 3). The bioclimatic temperature models were included in the 95% confidence set for all life history traits, except clutch size (Table 3). Natural logarithm of age at sexual maturity (β = -0.06; P < 0.01) and arcsine adult

survival (β = -0.08; $P < 0.05$) were both negatively related to Mean Temperature of Warmest Quarter (Fig 4).

Fig 4. Relationships between bioclimatic temperature (Mean Temperature of Warmest Quarter) and (A) clutch size, (B) clutch frequency, (C) age at sexual maturity, and (D) adult survival rate of freshwater turtles. Points are the median species values obtained from the literature (S1 Table), colored representing ln carapace length values. Solid black line is the GAM prediction. Grey shaded polygons show 95% confidence bands around the prediction.

The sensitivity analysis performed to examine the impact of harvest on freshwater turtle populations revealed that adult and juvenile survival rates had dramatically more impact on intrinsic rate of population growth than egg survival rate and fecundity (Fig 5). Tropical freshwater turtle species exhibited a moderately higher intrinsic rate of growth than temperate freshwater turtle species (Fig 5). Although, fecundity tended to be less in tropical species (Table 2, Table 4), comparing the minimum values necessary to result in positive population growth with GAM predictions showed that fecundity could be reduced by 28% in tropical compared with only 12% in temperate species (Table 4). Survival rates were estimated to be reducible by 35% in eggs, 24% in juveniles, and 5% in adults for tropical species, and 15%, 16%, and 7%, respectively for temperate species, without causing negative population growth (Table 4). However, overlap in estimations of population growth in relation to survival rates was very broad between tropical and temperate turtle species suggesting that, in aggregate the capacity for sustainable harvest of adults as an additive source of turtle mortality is constrained in tropical turtle species largely as it is in temperate zone species (Fig 5, Table 4).

Fig 5. Relationships between intrinsic rate of growth (*r*) and survival rates of (A) egg, (B) juvenile, and (C) adult, and (D) fecundity in freshwater turtles of tropical and temperate zones. Solid lines are the intrinsic rate of growth for temperate and tropical species, respectively.

Table 2. Influence of climate variables and latitude on freshwater turtle life history traits. Generalized Additive Models were used to predict responses of four freshwater turtle life history traits: clutch size, clutch frequency, age at sexual maturity and adult survival.

**Model**	**ln clutch size (n = 165)**			**ln clutch frequency (n = 102)**			**ln age at sexual maturity (n = 75)**			**arcsine adult survival (n = 37)**		
**Continuous latitude**	**Est.** [a]	**SE**	***p***	**Est.** [a]	**SE**	***p***	**Est.** [a]	**SE**	***p***	**Est.** [a]	**SE**	***p***
Intercept	0.78	0.05	***	0.12	0.05	*	0.75	0.02	***	0.12	0.03	***
Log carapace length	0.29	0.02	***	0.01	0.03		0.09	0.02	***	0.01	0.03	
Latitude	0.13	0.00	***	-0.09	0.04	*	0.06	0.02	**	0.04	0.03	
**Smooth**	**Edf/ref**	**F**	***p***	**Edf/ref**	**F**	***p***	**Edf/ref**	**F**	***p***	**Edf/ref**	**F**	***p***
Family	8.1/11	6.48	***	5.0 / 11	1.2	*	0.0 / 10	0.0		0.0 / 7	0.0	
R^2 ajust / Dev. Exp [b]	0.78 / 77.6%			0.12 / 17.9%			0.19 / 20.2			0.01 / 6.7		
**Categorical latitude**	**Est.** [a]	**SE**	***p***	**Est.** [a]	**SE**	***p***	**Est.** [a]	**SE**	***p***	**Est.** [a]	**SE**	***p***
Intercept	0.85	0.06	***	0.12	0.06	.	0.76	0.05	***	0.10	0.06	
Log carapace length	0.29	0.02	***	0.01	0.03		0.06	0.03	*	-0.01	0.04	
Latitude temp-trop	0.04	0.05		-0.06	0.08		0.02	0.05		0.08	0.06	
trop-temp	-0.13	0.05	*	0.06	0.09		-0.11	0.07		0.07	0.09	
tropical	-0.21	0.05	***	0.04	0.09		-0.02	0.08		-0.05	0.12	
**Smooth**	**Edf/ref**	**F**	***p***	**Edf/ref**	**F**	***p***	**Edf/ref**	**F**	***p***	**Edf/ref**	**F**	***p***
Family	7.9/11	6.27	***	2.7 / 11	0.4		4.7 / 10	0.9	.	2.5 / 7	0.6	
R^2 ajust / Dev. Exp [b]	0.76 / 75.8%			0.03 / 9.3%			0.24 / 32.2%			0.07 / 23.8%		
**Bioclimate - temp**	**Est.** [a]	**SE**	***p***	**Est.** [a]	**SE**	***p***	**Est.** [a]	**SE**	***p***	**Est.** [a]	**SE**	***p***
Intercept	0.76	0.05	***	0.12	0.04	**	0.72	0.04	***	0.11	0.03	**
Log carapace length	0.27	0.02	***	0.01	0.03		0.07	0.03	**	0.02	0.03	
Temp. warm quarter (bio10)	-0.01	0.02		0.03	0.03		-0.06	0.02	**	-0.08	0.03	*
**Smooth**	**Edf/ref**	**F**	***p***	**Edf/ref**	**F**	***p***	**Edf/ref**	**F**	***p***	**Edf/ref**	**F**	***p***

Family	7.0 / 11	4.39	***	3.4 / 11	0.6	.	5.4 / 10	1.2	*	0.0 / 7	0.0	
R^2 ajust / Dev. Exp [b]	0.72 / 69.8%			0.05 / 9.9%			0.31 / 37.7%			0.12 / 16.7%		
**Bioclimate - rain**	**Est.** [a]	**SE**	***p***	**Est.** [a]	**SE**	***p***	**Est.** [a]	**SE**	***p***	**Est.** [a]	**SE**	***p***
Intercept	0.76	0.05	***	0.12	0.04	**	0.73	0.05	***	0.15	0.03	***
Log carapace length	0.27	0.02	***	0.01	0.03		0.06	0.03	*	-0.01	0.03	
Rain dry quarter (bio17)	-0.03	0.02	.	0.02	0.03		0.00	0.02		0.02	0.04	
**Smooth**	**Edf/ref**	**F**	***p***	**Edf/ref**	**F**	***p***	**Edf/ref**	**F**	***p***	**Edf/ref**	**F**	***p***
Family	7.4/11	5.71	***	2.5 / 11	0.3		5.7 / 10	1.5	*	0.0 / 7	0.0	
R^2 ajust / Dev. Exp [b]	0.72 / 70.5%			0.02 / 7.0%			0.24 / 31.6%			-0.04 / 1.4%		

Each model contained Family as a random effect (smooth GAM term specified with "re" basis) and body size (ln transformed carapace length) as a parametric term; Asterisks indicate significant level of estimated parameters (*** $P < 0.001$; ** $P < 0.01$; * $P < 0.05$; '.' $P < 0.1$).

[a] Standardized regression coefficient (obtained by dividing the centered response values by their standard deviations) and associated standard error (SE).

[b] Model adjusted r-squared and deviance explained (%)

Table 3. Freshwater turtle life history model comparisons. Comparisons of the Generalized Additive Models created for each life history trait to estimate capacity for sustainable harvest in freshwater turtles. Models for each trait ordered by decreasing AICc (Akaike information criterion corrected for small sample sizes) values.

Life history trait	Model[a]	Dev. Exp	Loglik	BIC	AICc	Δ AICc	W_i AICc[b]
Clutch size							
	Continuous latitude	77.6	-69.35	207.05	168.09	0.00	1.00
	Categorical latitude	75.8	-80.04	237.57	193.80	25.71	0.00
	Bioclimate - rain	69.8	-95.48	256.12	218.85	50.77	0.00
	Bioclimate - temp	70.5	-98.96	261.27	224.98	56.90	0.00
Clutch frequency							
	Continuous latitude	17.9	-14.42	81.83	54.94	0.00	0.93
	Bioclimate - temp	9.9	-19.25	85.31	61.22	6.28	0.04
	Bioclimate - rain	7.0	-21.01	83.69	62.00	9.79	0.03
	Categorical latitude	9.3	-19.61	91.34	64.84	12.62	0.01
Age at sexual maturity							
	Continuous latitude	20.2	-28.82	79.24	68.52	0.00	0.57
	Bioclimate - temp	37.7	-19.46	92.49	69.13	0.62	0.42
	Bioclimate - rain	31.6	-22.86	99.50	76.08	7.56	0.01
	Categorical latitude	32.2	-22.62	104.54	79.42	10.91	0.00
Adult survival							
	Bioclimate - temp	16.7	14.16	-10.39	-16.31	0.00	0.85
	Continuous latitude	6.7	12.09	-6.27	-12.19	4.12	0.11
	Bioclimate - rain	1.4	11.09	-4.26	-10.18	6.13	0.04
	Categorical latitude	23.8	15.77	7.28	0.73	17.04	0.00

[a] Models used to predict natural history traits. Each model contained Family as a random effect (smooth term with “re” basis) and body size (log transformed carapace length) as a parametric (not smooth) effect. Continuous latitude included median latitude from all records (Table S1). Categorical latitude included four latitudinal classes: Temperate (species with latitudinal median and range within temperate zone), “Temp-Trop” (species with latitudinal median within temperate and range overlapping tropical zone/s), “Trop-Temp” (species with latitudinal median within tropical and range overlapping temperate zone/s), Tropical (species with latitudinal median and range within tropical zone). Bioclimate - temp included Mean Temperature of Warmest Quarter (WorldClim: bio10). Bioclimate - rain included Precipitation of Driest Quarter (WorldClim: bio17). Coefficients for individual variables in all models are presented in Table 2.

[b] Akaike weights (W_i) from largest to smallest. Predictions for each trait were obtained using variables from the 95% confidence subset of models, obtained by first ordering all models in the set by decreasing Akaike weight (W_i), and then sequentially summing the model W_i's in rank order.

Table 4. Demographic parameters used in population modelling to estimate capacity for sustainable harvest in freshwater turtles. Observed are median values derived from the scientific literature (S1 Table) and predicted values are from the 95% confidence set of GAM models (Table 3). "*r* min" are the minimum values necessary to obtain positive intrinsic rate of growth (*r*) as determined via sensitivity analysis (Fig 5).

	**Observed**		**Predicted**		***r* min**	
**Parameter**	**Temp.**	**Trop.**	**Temp.**	**Trop.**	**Temp.**	**Trop.**
Annual egg survival rate	0.200 [a]	0.200 [a]	0.200 [a]	0.200 [a]	0.170	0.130
Annual juvenile survival rate	0.766[b]	0.767[b]	0.746[b]	0.694[b]	0.630	0.530
Annual adult survival rate	0.880	0.882	0.857	0.798	0.800	0.760
Clutch size	8.8	7.0	7.3	5.2		
Clutch frequency	2.0	2.3	2.0	2.3		
Age at sexual maturity	8.3	7.8	8.6	7.3		
Fecundity	7.3	6.0	7.3	6.0	6.4	4.3

[a] Values derived from previous syntheses [32].
[b] Estimated as 13% less than the annual adult survival rates [59].

Discussion

The capacity of any species to cope with additive mortality is determined by the interplay of its life history traits [61-63]. Turtles are often declared to share integrated life history features [64] that make compensation for additive mortality associated with harvest infeasible [28]. Life history traits of many organisms are related to variation in environment [65, 66], climate [67] and their ecological interactions [61-63, 68, 69] and this study revealed that turtle life history is strongly related to latitude and ambient temperature. Yet although these trends might suggest an increase in capacity of tropical freshwater turtles to absorb additional mortality due to anthropogenic sources than in temperate zone species, once integrated in a synthetic population model tropical species appear to be as unable to absorb additive mortality as are temperate zone species.

The positive relationship we observed between clutch size and latitude is consistent with earlier studies [36, 41]. Turtles that inhabit higher (temperate) latitudes, have larger clutch size than turtles that inhabit low (tropical) latitudes. Similar patterns have been observed in mammals [67] and birds [68, 70]. Tokolyi, Schmidt (67) and McNamara, Barta (68) suggest this pattern is related to climate variability. Iverson, Balgooyen (41) concluded that higher juvenile competition due to shorter time period for development along with higher egg mortality associated with winter and climate uncertainty that creates temporary periods of low competition may make it more advantageous for temperate turtle species to produce more offspring ("more eggs in one basket" [33]) as a "bet hedging" strategy to exploit temporary resources. In addition, temperate turtle species typically have small egg size to speed development as an adaptation to short incubation times in temperate zone [17, 41]. As such, our findings support the suggestion that temperature zone turtles may have evolved to produce smaller egg size with larger clutch size than tropical species [33].

Larger clutch size in temperate turtle species may also act as a mechanism to compensate for low nesting frequency [33, 41]. We found that clutch frequency was negatively related to latitude. The general model of the interaction of environmental factors and reproductive output in turtles [33] suggests that high latitudes yield short reproductive seasons for turtles, resulting in lower clutch frequency. In addition, timing of nesting in turtles is correlated with temperature [36, 71]. Because tropical zones have a more stable warmer temperature all year long, more opportunities are available for turtles to lay eggs than in the temperate zone. Additionally, clutch mass (number of eggs x egg size) can also vary with latitude [33, 41], further studies are necessary to examine how egg size correlates to differences in population growth rates,

especially as egg size has been shown to be an important predictor of age at sexual maturity [32, 33].

The relationship between age at sexual maturity and latitude observed in this study is also in agreement with the earlier studies [72-74]. Turtles that inhabit high latitudes reach maturity at a later age than those inhabit low latitudes. This result is likely due to more stable and more productive climate conditions at low latitudes. As growth rate in turtles depends on temperature and food availability [75, 76], thus stable warm temperature and continuous food availability in low latitudes will generate faster growth rate to reach size at sexual maturity [33]. This conclusion is also supported by the inverse relationship between Mean Temperature of Warmest Quarter and age at sexual maturity. Although it has been suggested that turtles tend to have larger body size at higher latitudes [77] a recent review (compilation of 245 species) failed to uncover clear latitudinal trends in turtle body size [38]. These differences between studies (for example [77] evaluated variation within species from a sample of 23 species of mainly northern hemisphere and temperate turtles) seem to support the hypothesis that body size latitude relationships (e.g. Bergmann’s rule) maybe stronger for temperate turtle species. Large body size is thought to provide evolutionary advantages for temperate turtle species to cope with unfavorable environments e.g. via a relative increase in fasting endurance [36, 76]. As a result, temperate turtle species require longer time to reach size at sexual maturity, but increased size may provide for increased adult survivorship [32].

Adult survival and latitude were not strongly related perhaps because all turtles share in common the unique morphological feature: a rigid shell [28, 78, 79]. Turtle shells not only provide physical protection from predators [28], but also important physiological functions [78-80]. The optimum benefits from the shell are achieved when a turtle has reached adult size [28]

such that different environmental conditions at low and high latitudes may have little effect on adult survival rate because the shell ensures high survival regardless of ecological context.

It is important to note, however, that our failure to identify differences in survival rates may result from a lack of statistical power [53, 81]. Relatively few reports were available for survival rates of turtles at low (tropical) latitudes thereby possibly limiting the ability to detect differences might they exist. Clearly more long-term studies of turtle population biology in tropical regions are needed and would inform this analysis. This said, differences that may exist but are currently obscured by sampling variation would likely be modest and not likely to change the overall conclusions of this study.

The distinct life history characteristics of turtles at low latitudes (tropical zone) would seem to translate into greater opportunity for sustainable harvest of early stages than those at high latitudes (temperate zone, Fig 5, Table 4). However, our estimated annual sustainable harvest rate (5%) of adult turtles is considerably lower than typical thresholds for sustainable harvest rates (20%) estimated for long-lived animals [19, 82, 83]. In addition, similar to previous studies [29, 30, 34, 73, 84-87], high adult survival rates are estimated to be critical to maintain population stability due to their relatively greater contribution to population recruitment than other life stages [34]. Considering these results, harvesting wild adults would appear to present a high risk of causing population declines whether in the temperate or tropical regions, reinforcing the need to develop appropriately enforced alternate management options such as farming of captive reared turtles for meat [88].

Although adult harvest is clearly risky [9, 28, 87] there does appear to be some potential for sustainable exploitation of early stages of tropical freshwater turtle species. Indeed, egg

harvest may be more feasible, because it has low risk of causing population declines (Fig 5). Gibbs and Amato (28) suggest that significant additive mortality in the egg stage may not threaten population persistence and, Thorbjarnarson, Lagueux (8) identified that harvesting of eggs is the most promising strategy in the development of sustainable use programs for turtles. Integrating the conservation and harvest of eggs (for consumption, sale and/or rearing of hatchlings for the pet trade) has generated promising results for the conservation of some threatened tropical turtles e.g. *Podocnemis unifilis* in Peru [89, 90] and our analysis supports the idea that such actions could be feasible in other tropical turtle species.

We found that tropical populations could continue to grow if egg survival was reduced by up to 35%. We suggest that this surplus of eggs can be applied for both sustainable exploitation and conservation. A focus on management and sustainable exploitation of early life stages (e.g. consumption, pet trade) would also complement conservation actions that generally protect the most sensitive adult stages [9, 28]. We found that the margins for additive mortality are so tight (<10% in both tropical and temperate species) that the sustainable harvest of adult turtles will likely fail unless additional management actions are incorporated into conservation programs [9].

Integrated management that explicitly considers survival of all life stages is likely to generate more robust and timely increases in exploited turtle populations. Although egg survival produces a relatively small overall effect on population growth rates when compared to adult survival [28, 34], demographic simulations show that increasing survival of eggs and hatchlings can compensate for decreases in adult survival in at least one species of tropical turtle [91]. Additionally, increasing survival of early stages via community-based protection of turtle nesting beaches has been shown to provide conservation success for local communities [90], target species [90-93] and also non-target vertebrate and invertebrate taxa [92]. Further examples are

needed to understand how the predicted surplus in early life stages can be most effectively exploited so that populations can still increase to replace adults that remain widely targeted and threatened by additional anthropogenic impacts across tropical regions including climate change, forest loss and pollution [1, 9, 12, 18, 19].

An important caveat is that the population dynamics of temperate and tropical species in this study were evaluated using the same survival rate values for eggs due to lack of available published data on these parameters both in temperate and tropical species. Mechanisms of protection of egg and juvenile stages do not produce as large an effect on population growth as protecting adult survival [28], so our conclusions are likely to remain valid despite this untested assumption. That said, until data are available on typical nest and juvenile survival in temperate and tropical zones, the relative impact of harvest on populations of temperate and tropical species we estimated must remain tentative.

Together the results of this study imply that sustainable harvesting is difficult to apply as a conservation strategy, both in temperate and tropical turtle species, due to the biological limitations on turtle population growth imposed by their life history strategy everywhere. This said, Eisemberg, Rose (18) suggests that complete prohibition of harvesting as a conservation strategy in turtles will not be possible to implement in tropical areas and developing countries, where local communities have long history in using turtle meat and eggs. Conservation strategies that exclude local communities in their practices are often unsuccessful at protecting wildlife [94]. We reject the assumption often employed in temperate-zone turtle research that “all turtles are the same” yet also note that demographic differences we observed between temperate and tropical turtles do not translate into obviously greater opportunity for sustainable harvest of adults and juveniles in the tropics. Therefore, carefully constructed sustainable harvest programs

may present greater opportunities to succeed in the tropics if based on egg and hatchling stages, and should be considered further but cautiously for the regions that have a long history of harvesting turtles for subsistence use, particularly when the species possess density dependent mechanisms to compensate harvest, such as shown in *C. oblonga* [19, 42].

Acknowledgments

We thank N. E. Karraker, B. Underwood, and P. R. Sievert for discussions on ideas and their comments on this draft manuscript and to Y-H. Sung for providing life history data for the big-headed turtle. We thank Jordi Moya-Larano and two anonymous reviewers for their comments on an earlier version of the text.

Author Contributions

Conceived and designed the experiments: AR, JPG. Performed the experiments: AR, DN, JPG. Analyzed the data: AR, DN, JPG. Contributed reagents/materials/analysis tools: AR, DN, JPG. Wrote the paper: AR, DN, JPG.

Supporting Information

S1 Table. The life history traits data obtained from literature review.

References

1. Gibbons JW, Scott DE, Ryan TJ, Buhlmann KA, Tuberville TD, Metts BS, et al. The Global Decline of Reptiles, Deja Vu Amphibians. Bioscience. 2000;50(8):653 - 66. doi: https://doi.org/10.1641/0006-3568(2000)050[0653:TGDORD]2.0.CO;2.
2. Milner-Gulland EJ, Bennett EL. Wild Meat: The Bigger Picture. TRENDS in Ecology and Evolution. 2003;18(7):351 - 7. doi: https://doi.org/10.1016/S0169-5347(03)00123-X.
3. Santos-Fita D, Naranjo EJ, Rangel-Salazar JL. Wildlife Uses and Hunting Patterns in Rural Communities of the Yucatan Peninsula, Mexico. Journal of Ethnobiology and Ethnomedicine. 2012;8:38.
4. Altrichter M. Wildlife in the Life of Local People of the Semi-Arid Argentine Chaco. Biodiversity and Conservation. 2006;15:2719 - 36. doi: https://doi.org/10.1007/s10531-005-0307-5.

5. Mena P, Stallings JR, Regalado J, Cueva R. The Sustainability of Current Hunting Practices by the Huaorani. In: Robinson JG, Bennett EL, editors. Hunting for Sustainability in Tropical Forests. New York Columbia University Press; 2000.
6. Townsend WR. The Sustainability of Subsistence Hunting by the Siriono Indians of Bolivia. In: Robinson JG, Bennett EL, editors. Hunting for Sustainabillity in Tropical Forests. New York: Columbia University Press; 2000.
7. Moll D, Moll EO. The Ecology, Exploitation, and Conservation of River Turtles. New York: Oxford University Press; 2004.
8. Thorbjarnarson J, Lagueux CJ, Bolze D, Klemens MW, Meylan AB. Human Use of Turtles: A Wordwide Perspective. In: Klemens MW, editor. Turtle Conservation. Washington, DC: The Smithsonian Institution; 2000.
9. Spencer RJ, Dyke JU, Thompson MB. Critically evaluating best management practices for preventing freshwater turtle extinctions. Conservation Biology. 2017;31(6):1340-9. doi: https://doi.org/10.1111/cobi.12930.
10. Congdon JD, Greene JL, Gibbons JW. Biomass of Freshwater Turtles: A Geographic Comparison. American Midland Naturalist. 1986;115(1):165 - 73. doi: https://doi.org/10.2307/2425846.
11. Iverson JB. Biomass in Turtle Populations: A Neglected Subject. Oecologia. 1982;55(1):69 - 76. doi: https://doi.org/10.1007/BF00386720.
12. He F, Bremerich V, Zarfl C, Geldmann J, Langhans SD, David JN, et al. Freshwater megafauna diversity: Patterns, status and threats. Diversity and Distributions. 2018;24(10):1395-404. doi: https://doi.org/10.1111/ddi.12780.
13. Lovich JE, Ennen JR, Agha M, Gibbons JW. Where have all the turtles gone, and why does it matter? Bioscience. 2018;68(10):771-81. doi: https://doi.org/10.1093/biosci/biy095.
14. Saha A, McRae L, Dodd Jr CK, Gadsden H, Hare KM, Lukoschek V, et al. Tracking global population trends: Population time-series data and a living planet index for reptiles. Journal of Herpetology. 2018;52(3):259-68. doi: https://doi.org/10.1670/17-076.
15. Rhodin AG, Stanford CB, Van Dijk PP, Eisemberg C, Luiselli L, Mittermeier RA, et al. Global conservation status of turtles and tortoises (Order Testudines). Chelonian Conservation and Biology. 2018;17(2):135-61. doi: https://doi.org/10.2744/CCB-1348.1.
16. Klemens MW, Thorbjarnarson JB. Reptiles as a Food Resource. Biodiversity and Conservation. 1995;4:281 - 98. doi: https://doi.org/10.1007/BF00055974.
17. Moll EO, Moll D. Conservation of River Turtles. In: Klemens MW, editor. Turlte Conservation. Washington, DC: The Smithsonian Institution; 2000.
18. Eisemberg CC, Rose M, Yaru B, Georges A. Demonstrating Decline of an Iconic Species Under Sustained Indigenous Harvest - The Pig - Nosed Turtle (*Carettochelys insculpta*) in Papua New Guinea. Biological Conservation. 2011;144:2282 - 8. doi: https://doi.org/10.1016/j.biocon.2011.06.005.
19. Fordham DA, Georges A, Brook BW. Indigeneous Harvest, Exotic Pig Predation and Local Persistence of a Long - Lived Vertebrate: Managing a Tropical Freshwater Turtle for Sustainability and Conservation. Journal of Applied Ecology. 2008;45:52 - 62. doi: https://doi.org/10.1111/j.1365-2664.2007.01414.x.
20. Gamble T, Simons AM. Comparison of Harvested and Nonharvested Painted Turtle Populations. Wildlife Society Bulletin. 2004;32(4):1269 - 77. doi: https://doi.org/10.2193/0091-7648(2004)032[1269:COHANP]2.0.CO;2.
21. Sung YH, Karraker NE, Hau BCH. Demographic Evidence of Illegal Harvesting of an Endangered Asian Turtle. Conservation Biology. 2013;27(6):1421 - 8. doi: https://doi.org/10.1111/cobi.12102.
22. Iverson JB. Golbal Correlates of Species Richness in Turtles. The Herpetological Journal. 1992;2(3):77 - 81.

23. Mittermeier RA. South America's River Turtles: Saving Them by Use Oryx. 1978;14:222 - 30. doi: https://doi.org/10.1017/S0030605300015532.
24. Mali I, Vandewege MW, Davis SK, Forstner MRJ. Magnitude of the Freshwater Turtle Exports from the US: Long Term Trends and Early Effects of Newly Implemented Harvest Management Regimes. PLOS ONE. 2014;9(1):e86478. doi: https://doi.org/10.1371/journal.pone.0086478.
25. McNeely JA, Miller KR, Reid WV, Mittermeier RA, Werner TB. Conserving the World's Biological Diversity. Washington, D. C.: IUCN, Gland, Switzerland, WRI, CI, WWF - US, and the World Bank; 1990.
26. Webb GJ. Conservation and Sustainable Use of Wildlife - An Evolving Concept. Pacific Conservation Biology. 2002;8:12 - 26.
27. Fitzgerald LA. Tupinambis Lizards and People: A Sustainable Use Approach to Conservation Development. Conservation Biology. 1994;8(1):12 - 5.
28. Gibbs JP, Amato GD. Genetics and Demography in Turtle Conservation. In: Klemens MW, editor. Turtle Conservation. Washington, DC: The Smithsonian Institution; 2000.
29. Congdon JD, Dunham AE, van Loben Sels RC. Delayed Sexual Maturity and Demographics of Blanding's Turtles (*Emydoidea blandingii*): Implications for Conservation and Management of Long - Lived Organisms. Conservation Biology. 1993;7(4):826 - 33. doi: https://doi.org/10.1046/j.1523-1739.1993.740826.x.
30. Congdon JD, Dunham AE, van Loben Sels RC. Demographics of Common Snapping Turtles (*Chelydra serpentina*): Implications for Conservation and Management of Long - Lived Organisms. American Zoologist. 1994;34(3):397 - 408. doi: https://doi.org/10.1093/icb/34.3.397.
31. Doroff AM, Keith LB. Demography and Ecology of an Ornate Box Turtle (Terrapene ornata) Population in South-Central Wisconsin. Copeia. 1990;1990(2):387 - 99. doi: https://doi.org/10.2307/1446344
32. Iverson JB. Patterns of survivorship in turtles (order Testudines). Canadian Journal of Zoology. 1991;69:385 - 91. doi: https://doi.org/10.1139/z91-060.
33. Iverson JB. Correlates of Reproductive Output in Turtles (Order Testudines). Herpetological Monographs. 1992;6:25 - 42. doi: https://doi.org/10.2307/1466960
34. Heppell SS. Application of Life - History Theory and Population Model Analysis to Turtle Conservation. Copeia. 1998;1998(2):367 - 75. doi: https://doi.org/10.2307/1447430.
35. Burke VJ, Gibbons JW, Greene JL. Prolonged Nesting Forays by Common Mud Turtles (*Kinosternon subrurbrum*). American Midland Naturalist. 1994;131(190 - 195). doi: https://doi.org/10.2307/2426622.
36. Iverson JB, Higgins H, Sirulink A, Griffiths C. Local and Geographic Variation in the Reproductive Biology of the Snapping Turtle (*Chelydra serpentina*). Herpetologica. 1997;53(1):96 - 117.
37. Shine R, Iverson JB. Patterns of survival, growth and maturation in turtles. OIKOS. 1995;72:343 - 8. doi: https://doi.org/10.2307/3546119.
38. Angielczyk KD, Burroughs RW, Feldman CR. Do turtles follow the rules? Latitudinal gradients in species richness, body size, and geographic range area of the world's turtles. Journal of Experimental Zoology Part B: Molecular and Developmental Evolution. 2015;324(3):270-94. doi: https://doi.org/10.1002/jez.b.22602.
39. Santilli J, Rollinson N. Toward a general explanation for latitudinal clines in body size among chelonians. Biological Journal of the Linnean Society. 2018;124(3):381-93. doi: https://doi.org/10.1093/biolinnean/bly054.
40. Storey KB, Storey JM. Molecular physiology of freeze tolerance in vertebrates. Physiological Reviews. 2017;97(2):623-65. doi: https://doi.org/10.1152/physrev.00016.2016.
41. Iverson JB, Balgooyen CP, Byrd KK, Lyddan KK. Latitudinal Variation in Egg and Cutch Size in Turtles. Canadian Journal of Zoology. 1993;71:2448 - 61. doi: https://doi.org/10.1139/z93-341.

42. Fordham DA, Georges A, Brook BW. Demographic Response of Snake - Necked Turtles Correlates with Indigenous Harvest and Feral Pig Predation in Tropical Northern Australia. Journal of Animal Ecology. 2007;76:1231 - 43. doi: https://doi.org/10.1111/j.1365-2656.2007.01298.x.

43. Salguero-Gómez R, Jones OR, Archer CR, Bein C, de Buhr H, Farack C, et al. COMADRE: a global data base of animal demography. Journal of Animal Ecology. 2016;85(2):371-84. doi: https://doi.org/10.1111/1365-2656.12482.

44. Fick SE, Hijmans RJ. WorldClim 2: new 1-km spatial resolution climate surfaces for global land areas. International Journal of Climatology. 2017;37(12):4302-15. doi: https://doi.org/10.1002/joc.5086.

45. Sunday JM, Bates AE, Dulvy NK. Global analysis of thermal tolerance and latitude in ectotherms. Proceedings of the Royal Society B: Biological Sciences. 2010;278(1713):1823-30. doi: https://doi.org/10.1098/rspb.2010.1295.

46. Wood SN. Fast stable restricted maximum likelihood and marginal likelihood estimation of semiparametric generalized linear models. Journal of the Royal Statistical Society: Series B (Statistical Methodology). 2011;73(1):3-36. doi: https://doi.org/10.1111/j.1467-9868.2010.00749.x.

47. Wood SN. Generalized additive models: an introduction with R: Chapman and Hall/CRC; 2017.

48. Wood SN. A simple test for random effects in regression models. Biometrika. 2013;100(4):1005-10. doi: https://doi.org/10.1093/biomet/ast038.

49. Pedersen EJ, Miller DL, Simpson GL, Ross N. Hierarchical generalized additive models in ecology: an introduction with mgcv. PeerJ. 2019;7:e6876. doi: https://doi.org/10.7717/peerj.6876.

50. Rhodin A, Iverson J, Bour R, Fritz U, Georges A, Shaffer H, et al. Turtles of the world: annotated checklist and atlas of taxonomy, synonymy, distribution, and conservation status. Conservation biology of freshwater turtles and tortoises: a compilation project of the IUCN/SSC Tortoise and Freshwater Turtle Specialist Group Chelonian Research Monographs. 2017;7:1-292. doi: 10.3854/crm.7.checklist.atlas.v8.2017.

51. R Development Core Team. R: A language and environment for statistical computing. Vienna, Austria: R Foundation for Statistical Computing; 2019.

52. Burnham KP, Anderson DR. Model Selection and Multi-Model Inference: A Practical Information-Theoretic Approach. 2 ed. New York: Springer; 2002. 496 p.

53. Zuur AF, Ieno EN, Smith GM. Analysing Ecological Data. Gail M, Krickeberg K, Samet JM, Tsiatis A, Wong W, editors. New York: Springer; 2007.

54. Caswell H. Matrix population models: construction, analysis, and interpretation: Sinauer Associates; 1989.

55. Lefkovitch L. The study of population growth in organisms grouped by stages. Biometrics. 1965:1-18.

56. Crouse DT, Crowder LB, Caswell H. A Stage - Based Population Model fro Loggerhead Sea Turtles and Implications for Conservation. Ecology. 1987;68(5):1412 - 23. doi: https://doi.org/10.2307/1939225.

57. Stott I, Hodgson D, Townley S. popdemo: Demographic Modelling Using Projection Matrices. R package version 0.2-3 ed2016.

58. Stubben C, Milligan B. Estimating and analyzing demographic models using the popbio package in R. Journal of Statistical Software. 2007;22(11):1-23.

59. Pike DA, Pizzato L, Pike BA, Shine R. Estimating Survival Rates of Uncatchable Animals: The Myth of High Juvenile Mortality in Reptiles. Ecology. 2008;89(3):607 - 11. doi: https://doi.org/10.1890/06-2162.1.

60. Manly BF. Randomization, bootstrap and Monte Carlo methods in biology: Chapman and Hall/CRC; 2018.

61. Benton TG, Plaistow SJ, Coulson TN. Complex Population Dynamics and Complex Causation: Devils, Details and Demography. Proceedings of The Royal Society B. 2006;273:1173 - 81. doi: https://doi.org/10.1098/rspb.2006.3495.

62. Stearns SC. The Evolution of Life Histories. New York: Oxford University Press; 1992.

63. Stearns SC. The evolution of life history traits: a critique of the theory and a review of the data. Annual Review of Ecology and Systematics. 1977;8(1):145-71. doi: https://doi.org/10.1146/annurev.es.08.110177.001045.

64. Congdon JD, Gibbons JW. The Evolution of Turtle Life Histories. In: Gibbons JW, editor. Life History and Ecology of the Slider Turtle. Washington, D. C.: Smithsonian Institution Press; 1990.

65. Jonsson B, Jonsson N, Brodtkorb E, Ingebrigtsen PJ. Life-history traits of brown trout vary with the size of small streams. Functional Ecology. 2001;15(3):310-7. doi: https://doi.org/10.1046/j.1365-2435.2001.00528.x.

66. Macip-Ríos R, Ontiveros RN, Sánchez-León AT, Casas-Andreu G. Evolution of reproductive effort in mud turtles (Kinosternidae): the role of environmental predictability. Evolutionary Ecology Research. 2017;18(5):539-54.

67. Tokolyi J, Schmidt J, Barta Z. Climate and Mammalian Life Histories. Biological Journal of the Linnean Society. 2014;111(719 - 736). doi: https://doi.org/10.1111/bij.12238.

68. McNamara JM, Barta Z, Wikelski M, Houston AI. A Theoritical Investigation of the Effect of Latitude on Avian Life Histories. The American Naturalist. 2008;172(3):331 - 45. doi: https://doi.org/10.1086/589886.

69. Hutchings JA, Myers RA, García VB, Lucifora LO, Kuparinen A. Life-history correlates of extinction risk and recovery potential. Ecological Applications. 2012;22(4):1061-7. doi: https://doi.org/10.1890/11-1313.1.

70. Martin TE, Martin PR, Olson CR, Heidinger BJ, Fontaine JJ. Parental Care and Clutch Sizes in North and South American Birds. Science. 2000;287:1482 - 5. doi: https://doi.org/10.1126/science.287.5457.1482

71. Obbard ME, Brooks RJ. Nesting Migrations of the Common Snapping Turtle *Chelyydra serpentina*. Herpetologica. 1980;36:158 - 62.

72. Galbraith DA, Brooks RJ, Obbard ME. The Influence of Growth Rate on Age and Body Size at Maturity in Female Snapping Turtles (*Chelydra serpentina*) Copeia. 1989;1989(4):896 - 904. doi: https://doi.org/10.2307/1445975.

73. Spencer RJ. Growth Patterns of Two Widely Distributed Freshwater Turtles and a Comparison of Common Methods Used to Estimate Age. Australian Journal of Zoology. 2002;50:477 - 90. doi: https://doi.org/10.1071/ZO01066.

74. Tinkle DW. Geographic Variation in Reproduction, Size, Sex Ratio and Maturity of *Sternotherus odoratus* (Testudinata: Chelydridae). Ecology. 1961;42:68 - 76. doi: https://doi.org/10.2307/1933268

75. Dunham AE, Gibbons JW. Growth of the Slider Turtle. In: Gibbons JW, editor. Life History and Ecology of the Slider Turtle. Washington, D. C.: Smithsonian Institution Press; 1990. p. 135 - 45.

76. Gibbons JW, Semlitsch RD, Greene JL, Schibauer JP. Variation in Age and Size at Maturity of the Slider Turtle (*Pseudemys scripta*). The American Naturalist. 1981;117(5):841 - 5. doi: https://doi.org/10.1086/283774.

77. Ashton KG, Feldman CR. Bergmann's rule in nonavian reptiles: turtles follow it, lizards and snakes reverse it. Evolution. 2003;57(5):1151-63. doi: https://doi.org/10.1111/j.0014-3820.2003.tb00324.x.

78. Gilbert SF, Cebra - Thomas JA, Burke AC. How the Turtle Gets Its Shell. In: Wyneken J, Godfrey MH, Bels V, editors. Biology of Turtles: From Structures to Strategies of Life. USA: CRC Press, Taylor & Francis Group; 2008.

79. Zug GR, Vitt LJ, Caldwell JP. Herpetology: An Introductory Biology of Amphibians and Reptiles. 2nd ed. San Diego, California, USA: Academic Press; 2001.

80. Jackson DC. How a Turtle's Shell Helps It Survive Prolonged Anoxic Acidosis. News Physiological Science. 2000;15:181 - 5. doi: https://doi.org/10.1152/physiologyonline.2000.15.4.181.

81. Crawley MJ. The R Book. UK: John Wiley & Sons, Ltd.; 2007.
82. Robinson JG, Bennett EL. Carrying Capacity Limits to Sustainable Hunting in Tropical Forests. In: Robinson JG, Bennett EL, editors. Hunting for Sustainability in Tropical Forests. New York: Columbia University Press; 1990.
83. Robinson JG, Bodmer RE. Towards Wildlife Management in Tropical Forests. The Journal of Wildlife Management. 1999;63(1):1 - 13. doi: https://doi.org/10.2307/3802482.
84. Blamires SJ, Spencer RJ, King P, Thompson MB. Population Parameters and Life - Table Analysis of Two Coexisting Freshwater Turtles: Are the Bellinger River Turtle Populations Threatened? Wildlife Research. 2005;32(4):339 - 47. doi: https://doi.org/10.1071/WR04083.
85. Macip-Rios R, Brauer-Robleda P, Zuniga-Vega JJ, Casas-Andreu G. Demography of two populations of the Mexican mud turtle (*Kinosternon integrum*) in central Mexico. Herpetological Journal. 2011;21:235 - 45.
86. Spencer RJ, Thompson MB. Experimental Analysis of the Impact of Foxes on Freshwater Turtle Populations. Conservation Biology. 2005;19(3):845 - 54. doi: https://doi.org/10.1111/j.1523-1739.2005.00487.x.
87. Zimmer-Shaffer SA, Briggler JT, Millspaugh JJ. Modeling the effects of commercial harvest on population growth of river turtles. Chelonian Conservation and Biology. 2014;13(2):227-36. doi: https://doi.org/10.2744/CCB-1109.1.
88. Mali I, Wang H-H, Grant WE, Feldman M, Forstner MRJ. Modeling Commercial Freshwater Turtle Production on US Farms for Pet and Meat Markets. PLOS ONE. 2015;10(9):e0139053. doi: https://doi.org/10.1371/journal.pone.0139053.
89. Sinovas P, Price B, King E, Hinsley A, Pavitt A. Wildlife trade in the Amazon countries: an analysis of trade in CITES listed species. Cambridge, UK: Technical report prepared for the Amazon Regional Program, 2017.
90. Harju E, Sirén AH, Salo M. Experiences from harvest-driven conservation: Management of Amazonian river turtles as a common-pool resource. Ambio. 2017;47:327. doi: https://doi.org/10.1007/s13280-017-0943-5.
91. Norris D, Peres CA, Michalski F, Gibbs JP. Prospects for freshwater turtle population recovery are catalyzed by pan-Amazonian community-based management. Biological Conservation. 2019;233:51-60. doi: https://doi.org/10.1016/j.biocon.2019.02.022.
92. Campos-Silva JV, Hawes JE, Andrade PC, Peres CA. Unintended multispecies co-benefits of an Amazonian community-based conservation programme. Nature Sustainability. 2018;1(11):650. doi: https://doi.org/10.1038/s41893-018-0170-5.
93. Norris D, Michalski F, Gibbs JP. Community involvement works where enforcement fails: conservation success through community-based management of Amazon river turtle nests. PeerJ. 2018;6:e4856. doi: https://doi.org/10.7717/peerj.4856.
94. Pimhert MP, Pretty JN. Diversity and Sustainability in Community Based Conservation. In: Kothari A, Pathak N, Anuradha RV, Taneja B, editors. Communities and Conservation: Natural Resource Management in South and Central Asia. Sage Publication: New Delhi, India; 1998. p. 58 - 77.

Figure 1

Click here to access/download;Figure;Fig1.tif

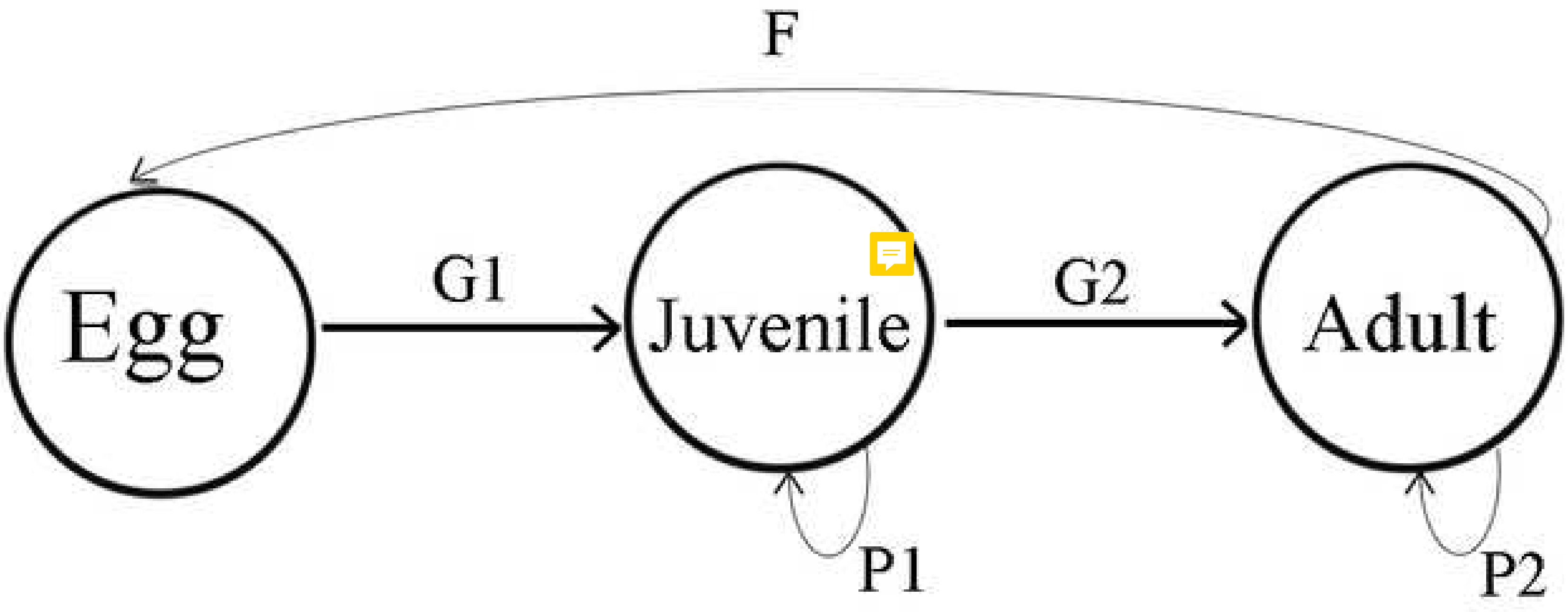

Figure 2

Click here to access/download;Figure;Fig2.tif

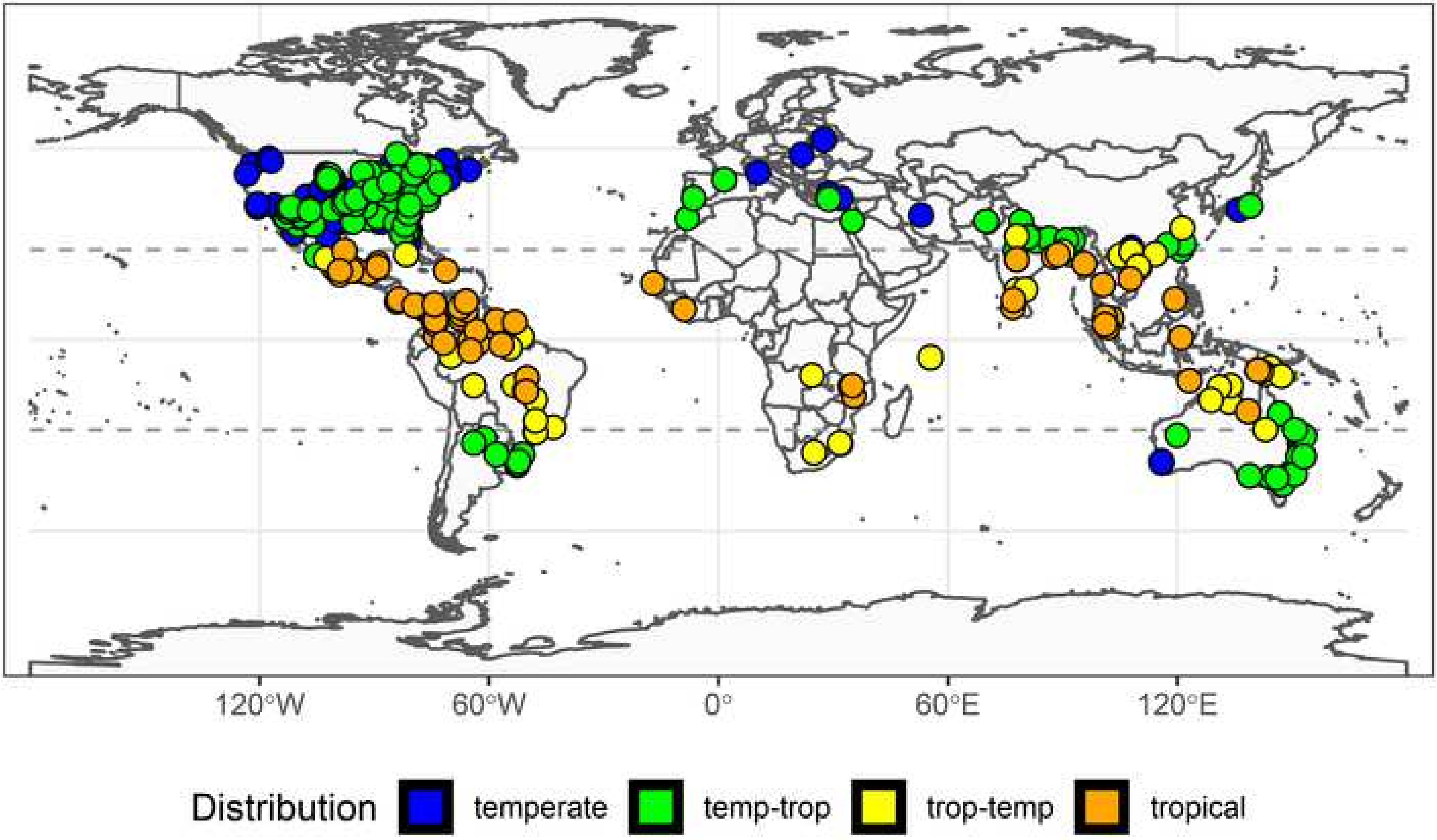

Figure 3

Click here to access/download;Figure;Fig3.tif

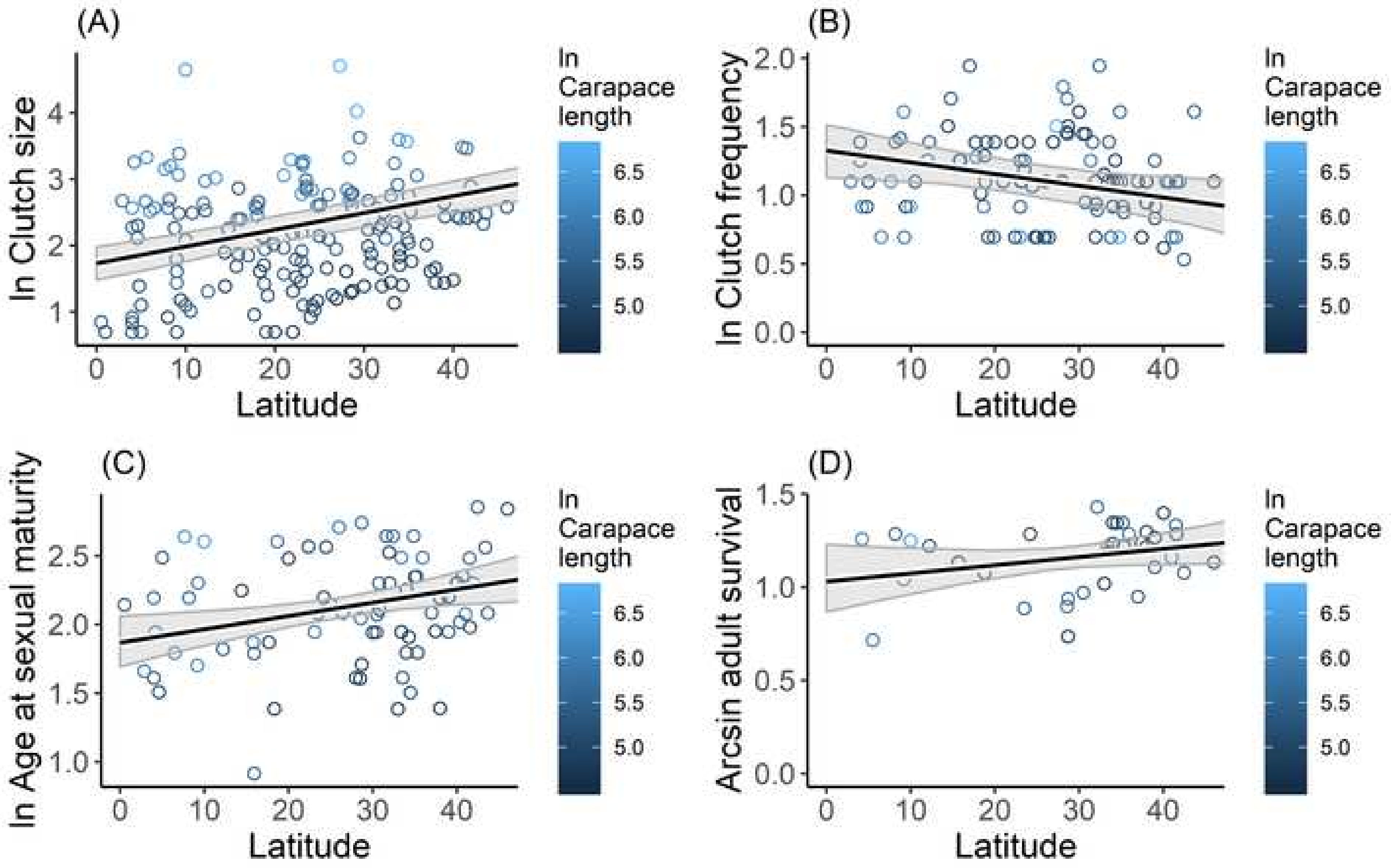

Figure 4

Click here to access/download;Figure;Fig4.tif

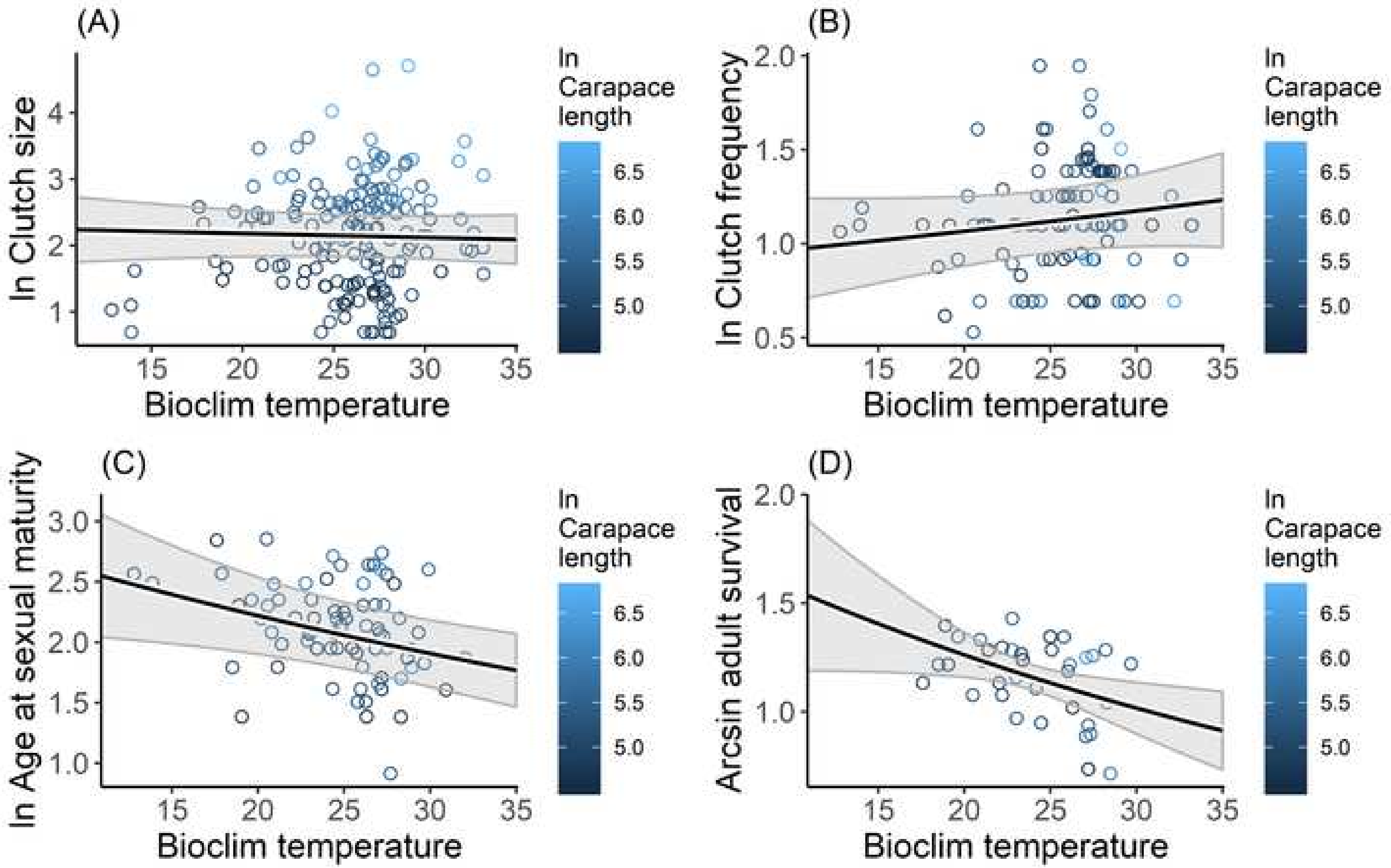

Figure 5

Click here to access/download;Figure;Fig5.tif

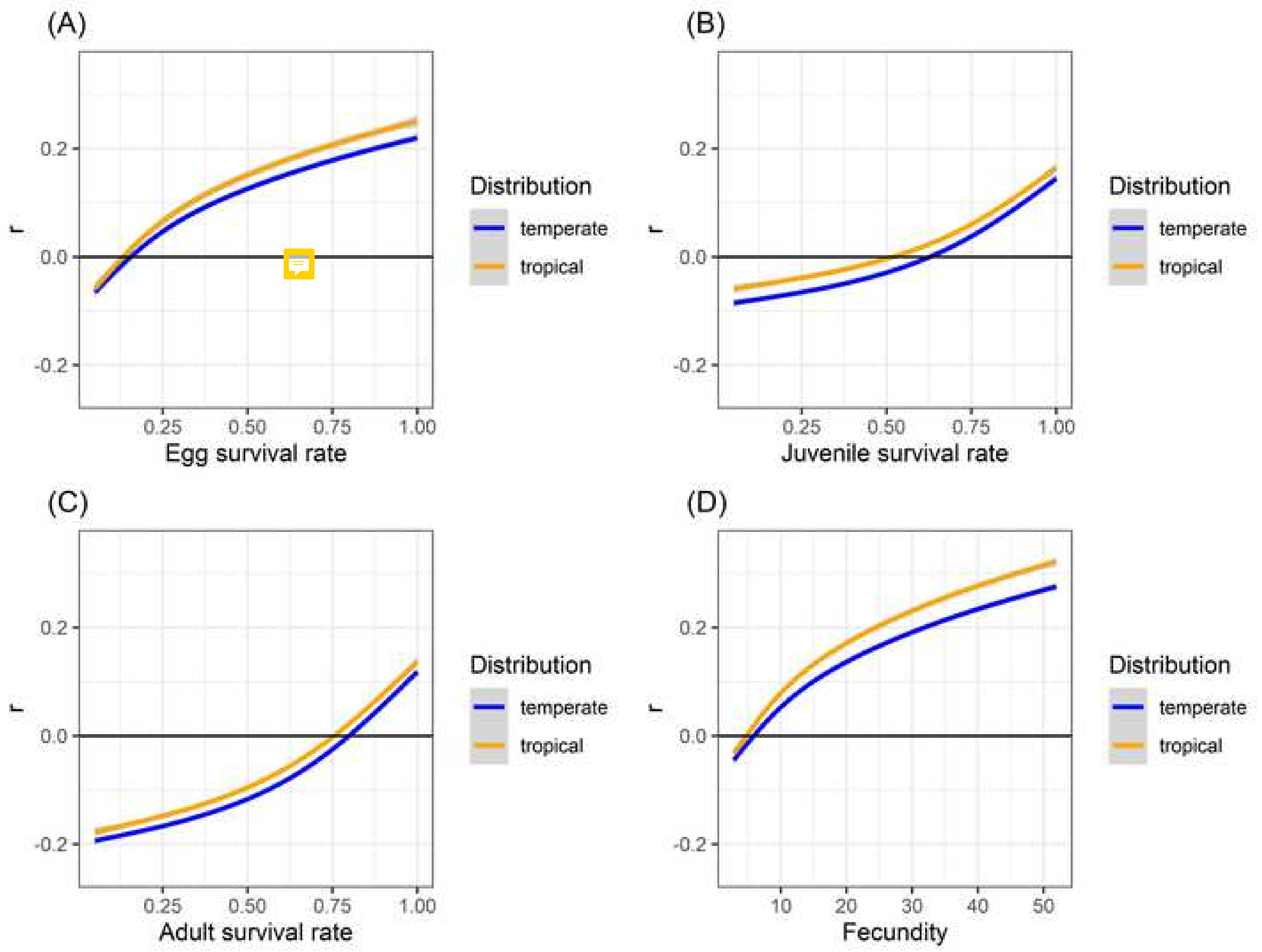

Click here to access/download
Supporting Information
S1_Table.docx

From: **PLOS ONE** <em@editorialmanager.com>
Date: Sat, Dec 5, 2015 at 5:08 PM
Subject: PLOS ONE Decision: Revise [PONE-D-15-44735] - [EMID:be9d1b1768505f52]
To: angga rachmansah <angga.rachmansah@gmail.com>

PONE-D-15-44735
Biological Feasibility of Sustainable Harvesting Programs as a Conservation Strategy for Tropical Freshwater Turtles
PLOS ONE

Dear Mr rachmansah,

Thank you for submitting your manuscript to PLOS ONE. After careful consideration, we feel that it has merit, but is not suitable for publication as it currently stands. Therefore, my decision is "Major Revision."

We invite you to submit a revised version of the manuscript that addresses the points below:

*** I liked this ms very much and think that after you address the comments of the referees and those of my own it should be ready for publication. However, I must warn you that these changes may be substantial.

Both reviews are excellent and hope that you can make a good use of them. Rev. #1 provides relevant comments to improve your ms and especially points to missing literature that should be cited and Rev. #2 suggests expanding and changing the statistical analyses and has main concerns on the modelling which should be addressed before publication is granted. This reviewer suggests to look at explanatory patterns within each latitude (basically what it could be summarized as looking for a predictor*latitude interaction on life history traits). He/she also asks for you to state your predictions/expectations beforehand in your Intro and Methods section.

I found that the Discussion is the weakest part of the ms, particularly because you mostly address the turtle literature but do not frame it in the extensive literature on life history traits. I provide some hints to help improving the Discussion and finding support to your findings from what we know across organisms and from theoretical standpoints.

Unfortunately your ms did not have line nor page numbers. In order to comment on it I therefore copied the actual text that I am referring to without reference to any page or line numbers.

What is “A” in the model?
Our reply: We have clarified the text (p l) as follows “The discrete stage based lifecycle (Fig 1) can be presented as a population projection matrix “A” as follows:”

A sensitivity analysis changing the 0.2 egg survival rate value would be advisable, as we know that usually interactions are stronger in the tropics (literature by Dobzhansky, Schemske, summarized in Bekmam 2013 Ecol. Lett. 16:1054-1060) and this value could actually change with latitude. The same could apply to other sources of (adult and juvenile) mortality, as in principle predation and parasitism (sources of mortality) should be higher in the tropics.
Our reply: We include results from the sensitivity analysis in Figure 5. We have now included estimate of variation around this 0.2 value in our sensitivity analysis (standard deviation from Iverson 1991). The egg survival value is likely to vary but the most robust synthesis available

suggest that 0.2 is a suitable value for freshwater species (Iverson 1991). Our estimates of juvenile mortality are expressed as a percentage of adult mortality. These values and their range (from species level data) are included in the sensitivity analysis. There are therefore differences in juvenile survival values between temperate and tropical zone species (see updated Table 4 and Figure 5). We have updated and clarified the Methods to reflect this. We are not aware of any studies that present empirical data necessary to robustly inform a more in-depth analysis. We hope that future studies can further refine these analyses.

Gibbs JP, Amato GD. Genetics and Demography in Turtle Conservation. In: Klemens MW, editor. Turtle Conservation. Washington, DC: The Smithsonian Institution; 2000.

Iverson JB. Patterns of survivorship in turtles (order Testudines). Canadian Journal of Zoology. 1991;69:385 - 91.

In particular, the above could also respond to this paragraph: "An important caveat is that the population dynamics of temperate and tropical species in this study were evaluated using the same survival rate values for egg and juvenile stages due to lack of available published data on these parameters both in temperate and tropical species. Mechanisms of protection of egg and juvenile stages do not produce as large an effect on population growth as to protections adult survival [24], so our conclusions may remain valid despite this unassessed assumption. That said, until data are available on typical nest and juvenile survival in temperate and tropical zones, the relative impact of harvest on populations of temperate and tropical species we estimated must remain tentative."
Our reply: We share data and code to encourage further studies to address the broad range of questions that can be asked with the data we have compiled and shared. We believe that until data are available on typical nest and juvenile survival in temperate and tropical zones, the relative impact of harvest on populations of temperate and tropical species we estimated must remain tentative.

The reported values do not match the text, please check: "whereas natural logarithm of clutch frequency ($\beta = -0.04$; $P < 0.001$) was positively related to annual mean temperature (Fig 4)."
Our reply: Based on the helpful suggestions from both the editor and reviewers we have extensively updated the analysis and results. The text has been revised to ensure consistency of the reported values.

"confidence intervals" in regression fits (where the spread of the confidence region changes with X) should be "confidence bands". Please, change accordingly.
Our reply: We have updated the text accordingly.

Discussion
Please provide R2 values of your lmer models (see Nakagawa 2013 Meth Ecol Evol and library MuMIn in R) to support this sentence in the Discussion: "The results of this study revealed that turtle life history is strongly related to latitude and ambient temperature."

Our reply: Based on the helpful suggestions from the editor and reviewers we have extensively updated the analysis and results. We have included r2 values in the revised version (Table 3).

A low p-value or a regression coefficient without standardization is not enough to support your claims. A large proportion of the variance may be still left to be explained.
Our reply: Based on the helpful suggestions from the editor and reviewers we have extensively

updated the analysis and results. We have also revised the text to ensure conclusions and results are supported by the data and analysis presented.

"Iverson et al. [32] concluded that higher juvenile competition due to shorter time period for development along with higher egg mortality associated with winter and climate uncertainty that creates temporary periods of low competition may make it more advantageous for temperate turtle species to produce more offspring as a "bet hedging" strategy to exploit temporary resources." This is too speculative, especially since you use a 0.2 egg survival rate from one single place because there are not comparative data available. Is there evidence that buried eggs suffer mortality from cold temperatures?

Our reply: We have now added the reference of Iverson 1991 [1] to the sentence. This is one of the classic turtle demography references, including a pioneering and meticulous compilation of 81 age-class-specific survivorship values representing 30 turtle species. It is not a "single place". Iverson [1] provides a combined early stage (egg – to 1 year) annual survival estimate of 0.215+0.188 for freshwater turtles (includes eggs, hatchlings, 1 year that were combined due to "lack of significant differences in survivorship across these three age-classes"). Egg mortality is affected by myriad factors (see supporting references below). But for freshwater turtle species survival estimates of all these early stages are firmly anchored around 0.2 and the SD value (see above) also suggests a consistently low survival value for early stages. In addition for long lived species with multiple reproductive events and in the case of turtles multiple egg laying events, egg survival has consistently been demonstrated to be far less important than survival in juvenile and adult stages [2,3]. Buried eggs can suffer mortality from extreme temperatures (too hot and too cold) and desiccation in both temperate and tropical zones. Turtles have a number of behavioral (e.g. nest depth, substrate choice) and physiological (egg shell thickness, size and shape) adaptations that can help reduce egg mortality (see examples in supporting references below). Such factors are species and location specific. However, we feel that such factors do not affect our conclusions and are not highly relevant to our Discussion. For example, our main finding is that adult harvest is extremely risky (likely to be unsustainable) in both tropical and temperate turtle species. This conclusion is based on an unprecedented compilation of species level demographic data and robust modeling of population dynamics (including sensitivity analysis). For all these reasons we prefer to retain the use of 0.2 egg survival and hope future studies can further refine these analyses based on the data and code we share.

[1] Iverson JB. Patterns of survivorship in turtles (order Testudines). Canadian Journal of Zoology. 1991 Feb 1;69(2):385-91.
[2]Gibbs JP, Amato GD. Genetics and Demography in Turtle Conservation. In: Klemens MW, editor. Turtle Conservation. Washington, DC: The Smithsonian Institution; 2000.
[3] Heppell SS. Application of Life - History Theory and Population Model Analysis to Turtle Conservation. Copeia. 1998;1998(2):367 - 75.

Supporting references relevant for egg survivorship.
Iverson JB, Ewert MA. Physical characteristics of reptilian eggs and a comparison with avian eggs. Egg Incubation: Its Effect on Embryonic Development in Birds and Reptiles. Cambridge University Press, New York, New York, USA. 1991 Dec 5:87-100.
Mitchell NJ, Rodriguez N, Kuchling G, Arnall SG, Kearney MR. Reptile embryos and climate change: modelling limits of viability to inform translocation decisions. Biological Conservation. 2016 Dec 1;204:134-47.

Packard GC, Tracy CR, Roth JJ. The physiological ecology of reptilian eggs and embryos and the evolution of viviparity within the Class Reptilia. Biological Reviews. 1977 Feb;52(1):71-105.
Packard MJ, DeMarco VG. Eggshell structure and formation in eggs of oviparous reptiles. Egg Incubation: Its Effect on Embryonic Development in Birds and Reptiles. Cambridge University Press, New York, New York, USA. 1991 Dec 5:53-69.
Packard GC. Water relations of chelonian eggs and embryos: is wetter better? American Zoologist. 1999 Apr 1;39(2):289-303.

Furthermore, "bet hedging" could equally be important in abiotic (temperate) driven environments as in biotic (interactions) driven environments. Actually, a biotic environment could be more variable and unpredictable (see Moya-Laraño 2010 Open Ecol. J. 3:1-10). More eggs with latitude go indeed against a pattern for higher mortality in the tropics. However, this could merely reflect that selection is targeting egg size in the tropics (larger eggs could help offspring escaping stronger predation pressure in the tropics) and that a fundamental egg-size/egg-number trade-off makes then fewer eggs to be laid in the tropics (see Verdeny-Vilalta et al. 2015 J. Evol. Biol. 28:1225-1233 for a similar pattern). Of course, you the explain later in the text that the shell may make a big difference for predation. Is that the case also for turtle-lings? I see you refer to this issue later on in the text and indeed adults are better protected, thus.

This hypothesis seems very plausible according to what we know. But please, do explicitly refer to the egg-size/egg-number trade-off explicitly, not just implicitly as you do here. "In addition, Moll and Moll [12] concluded that temperate turtle species typically have small egg size to speed development as an adaptation to short incubation times in temperate zone [32]. As such, temperature zone turtles may have evolved to produce smaller egg size with larger clutch size than tropical turtle species."

Our reply: We have included egg-size/egg-number trade off specifically as follows "Additionally, clutch mass (number of eggs x egg size) can also vary with latitude[33, 41], further studies are necessary to examine how egg size correlates to differences in population growth rates, especially as egg size has been shown to be an important predictor of age at sexual maturity [32, 33]."…….

"The interaction between age at sexual maturity and latitude observed in this study is also in agreement with the earlier studies [62-64]."
please change it with
"The relationship between age at sexual maturity and latitude observed in this study is also in agreement with the earlier studies [62-64]."
you are not testing statistical interactions, which mean rather the opposite (multiplicative vs additive effects).
Our reply: We have made the correction.

The following sentence is fundamentally incomplete:
"Large body size provides advantages for temperate turtle species to cope with unfavorable environment through increasing their fitness [33, 66]. As a result, temperate turtle species require longer time to reach size at sexual maturity."
Our reply: We have rephrased as follows to provide a link to the following paragraph which deals with adult survival: "As a result, temperate turtle species require longer time to reach size at sexual maturity, but increased size may provide for increased adult survivorship [32]. "

Even though larger body sizes increase cold hardiness, to reach this body size turtles will have to face with longer periods of time (several seasons!), which increases the probability of death before reproduction and, in addition, they will have to go through all those body size stages that are more dangerous (in which they are more vulnerable). You are implicitly referring to Bergmann's and inverse Bergmann's rule here. Please, do check a bit the extensive recent body of literature on this issue and discuss the explanations researchers have offered for what it may be occurring in ectotherms. A temperature constraint to reach maturation in a single season may be more appropriate as an explanation for the increase in large body size with latitude, I think. furthermore, this part "species to cope with unfavorable environment through increasing their fitness" is a circular argument. You mean that body size is larger because it provided a fitness advantage in the (evolutionary) past? increasing the fitness to cope with the unfavorable environment is biologically non-sense as fitness is an (evolutionary meaningful) response. Please change accordingly.
Our reply: We have extensively revised this paragraph on page 23 (L) to clarify the text following the Editors suggestions.

For more information on how to upload your revised submission, see our video: http://blogs.plos.org/everyone/2011/05/10/how-to-submit-your-revised-manuscript/

If you choose not to submit a revision, please notify us.

Yours sincerely,

Jordi Moya-Larano
Academic Editor
PLOS ONE

Journal requirements:

When submitting your revision, we need you to address these additional requirements.

Please ensure that your manuscript meets PLOS ONE's style requirements, including those for file naming. The PLOS ONE style templates can be found at http://www.plosone.org/attachments/PLOSOne_formatting_sample_main_body.pdf and http://www.plosone.org/attachments/PLOSOne_formatting_sample_title_authors_affiliations.pdf

** It was noted by our internal staff that Figure 2 in your manuscript have been previously copyrighted.
Our reply: The revised version has figures that are fully compliant with plosone copyright guidelines. We clarify this in the figure legend as follows: "Fig 2. Distribution of freshwater turtle studies. Geographic distribution of data on freshwater turtle life history traits obtained from the literature (S1 Table) to estimate capacity for sustainable harvest in freshwater turtles. Color of study locations represent the latitudinal distribution of the study species. Dashed horizontal lines show Tropic of Cancer and Tropic of Capricorn. The background map was obtained from the 1:110m Natural Earth country and geographic lines maps (http://www.naturalearthdata.com)."

Should your paper be accepted, all images will published under PLOS' CC BY 4.0 license, which means that they will be freely available online, and any third party is permitted to access, download, copy, distribute, and use these materials in any way, even commercially, with proper attribution. For more information, see our Figure guidelines:
http://www.plosone.org/static/figureGuidelines#policies

With regards to the previously copyrighted figures in your submission, we require you to either present written permission from the copyright holder to publish these figures, or remove the figures from your submission.
Our reply: The revised version has figures that are fully compliant with plosone copyright guidelines. We clarify this in the figure legend as follows: "Fig 2. Distribution of freshwater turtle studies. Geographic distribution of data on freshwater turtle life history traits obtained from the literature (S1 Table) to estimate capacity for sustainable harvest in freshwater turtles. Color of study locations represent the latitudinal distribution of the study species. Dashed horizontal lines show Tropic of Cancer and Tropic of Capricorn. The background map was obtained from the 1:110m Natural Earth country and geographic lines maps (http://www.naturalearthdata.com)."

1. To seek permission from the original copyright holder of Figure 2 to publish it under the specific Creative Commons Attribution License (CCAL), CC BY 4.0, we recommend that you contact the original copyright holder with the following text:

"I request permission for the open-access journal PLOS ONE to publish XXX under the Creative Commons Attribution License (CCAL) CC BY 4.0 (http://creativecommons.org/licenses/by/4.0/). Please be aware that this license allows unrestricted use and distribution, even commercially, by third parties. Please reply and provide explicit written permission to publish XXX under a CC BY license."

Please upload the granted permission to the manuscript as a supporting information file. In the figure caption of the copyrighted figure, please include the following text: "Reprinted from [ref] under a CC BY license, with permission from [name of publisher], original copyright [original copyright year]."

2. If you are unable to obtain permission from the original copyright holder, please either i) remove the figure or ii) supply a replacement figure that complies with the CC BY 4.0 license, which can be for illustrative purposes only. Please check copyright information on all replacement figures and update the figure caption with source information. If applicable, please specify in the figure caption text when a figure is similar but not identical to the original image, and is therefore for illustrative purposes only.

3. It was noted that the figure(s) in question contains a map or satellite image. PLOS ONE is unable to publish previously copyrighted maps or satellite images, or images created using proprietary data. For these reasons, we cannot publish images generated by Google software (Google Maps, Street View, and Earth). If the content of the manuscript depends on the use of Google software, you may need to provide replacement images that are representative of the Google-generated images. The following resources for replacing copyrighted map figures may be helpful:

OpenStreetMap (data, but not generated images, are open): http://www.openstreetmap.org/

USGS National Map Viewer (public domain): http://viewer.nationalmap.gov/viewer/

The Gateway to Astronaut Photography of Earth (public domain): http://eol.jsc.nasa.gov/sseop/clickmap/

Maps at the CIA (public domain): https://www.cia.gov/library/publications/the-world-factbook/index.html and https://www.cia.gov/library/publications/cia-maps-publications/index.html

NASA Earth Observatory (public domain): http://earthobservatory.nasa.gov/

USGS EROS (Earth Resources Observatory and Science (EROS) Center) (public domain): http://eros.usgs.gov/#

Grass GIS (geographic information system) analysis software (data, but not generated images, are open): http://grass.osgeo.org/

Grass GIS (geographic information system) analysis software (data, but not generated images, are open): http://grass.osgeo.org/

Our reply: The revised version has figures that are fully compliant with plosone copyright guidelines. We clarify this in the figure legend as follows: "Fig 2. Distribution of freshwater turtle studies. Geographic distribution of data on freshwater turtle life history traits obtained from the literature (S1 Table) to estimate capacity for sustainable harvest in freshwater turtles. Color of study locations represent the latitudinal distribution of the study species. Dashed horizontal lines show Tropic of Cancer and Tropic of Capricorn. The background map was obtained from the 1:110m Natural Earth country and geographic lines maps (http://www.naturalearthdata.com)."

Reviewers' comments:

Reviewer's Responses to Questions

Comments to the Author

1. Is the manuscript technically sound, and do the data support the conclusions?

The manuscript must describe a technically sound piece of scientific research with data that supports the conclusions. Experiments must have been conducted rigorously, with appropriate controls, replication, and sample sizes. The conclusions must be drawn appropriately based on the data presented.

Reviewer #1: Yes

Reviewer #2: Yes

---

2. Has the statistical analysis been performed appropriately and rigorously?

Reviewer #1: Yes

Reviewer #2: No

---

3. Does the manuscript adhere to the PLOS Data Policy?

Authors must follow the PLOS Data policy, which requires authors to make all data underlying the findings described in their manuscript fully available without restriction. Please refer to the author's Data Availability Statement in the manuscript. All data and related metadata must be deposited in an appropriate public repository, unless already provided as part of the submitted article or supporting information. If there are restrictions on the ability of authors to publicly share data—e.g. privacy or use of data from a third party—these reasons must be specified.

Reviewer #1: Yes

Reviewer #2: Yes

---

4. Is the manuscript presented in an intelligible fashion and written in standard English?

PLOS ONE does not copyedit accepted manuscripts, so the language in submitted articles must be clear, correct, and unambiguous. Any typographical or grammatical errors should be corrected at revision, so please note any specific errors here.

Reviewer #1: Yes

Reviewer #2: Yes

---

5. Review Comments to the Author

Please use the space provided to explain your answers to the questions above. You may also include additional comments for the author, including concerns about dual publication, research ethics, or publication ethics. (Please upload your review as an attachment if it exceeds 20,000 characters)

Reviewer #1: This is a well written and appropriately analyzed manuscript about freshwater turtle sustainability. I have several minor comments that are worth mentioning in the manuscript:
Although extensive amount of literature has been cited that shows overall understanding of turtle natural history, there are several articles that have been recently published and that are relevant to this article:
1. In the introduction in the second paragraph, the authors should mention that although the greatest pressure on turtle harvest occurs in tropical areas, these high pressures can lead to regional population collapse and as a consequence create pressures in other regions of the world (Mali et al. 2014)
Mali, I., M.W. Vandewege, S.K. Davis, and M.R.J. Forstner. 2014. Magnitude of the freshwater turtle exports from the US: long term trends and early effects of newly implemented harvest management regimes. PLoSONE 9(1): e86478. doi:10.1371/journal.pone.0086478.
Our reply: Based on the helpful comments from the Editor and reviewers we have extensively revised and updated the literature cited. We have included in the revised text as follows "Additionally, unsustainable exploitation in tropical areas can also lead to regional population collapse and as a consequence create pressures in other regions of the world [24]."

2. Discussion paragraph 6- In talking about risks of harvesting adults and juveniles, the study of Zimmer-Shaffer et al. 2014 should also be mentioned.
Zimmer-Shaffer, S.A., J.T. Briggler, and J.J. Millspaugh. 2014. Modeling the effects of commercial harvest on population growth of river turtles. Chelonian Conservation and Biology 13(2):227-237.
Our reply: We have added as follows "In addition, similar to previous studies [29, 30, 34, 74, 85-88], high adult and juvenile survival rates are estimated to be critical to maintain population stability due to their relatively greater contribution to population recruitment than other life stages [34]."

Additional minor comments:
Citation [45] is missing in the body of the article. Please address
Our reply: Based on the helpful comments from the Editor and reviewers we have extensively revised and updated the literature cited. We believe references and citations are consistent.

Abstract- "Turtles at low latitudes (tropical zones) exhibit similar adult survival rates...."
Delete "similar adult survival rates"
Our reply: Corrected as suggested.

Introduction, Paragraph 2 and 3- Em dash was used for "15-18" and "25-27" citation and in the rest of the article hyphen was used.
Our reply: We have carefully revised all formatting in the text and references.

Introduction, Paragraph 3- Last citation could be fixed from [24,29-29] to [24,29]
Our reply: We have carefully revised all formatting in the text and references.

Results, Paragraph 1- In the last sentence replace "Just" with "Only" and ";" with "while"
Our reply: Corrected as suggested.

Discussion, Paragraph 5- delete "only" in the sentence "It is important to note....."
Our reply: Corrected as suggested. We have extensively revised the Discussion to improve flow and clarity.

Discussion, Paragraph 6- Correct the sentence "Egg harvest is may be....." to "Egg harvest may be more feasible..."
Our reply: Corrected as suggested. We have extensively revised the Discussion to improve flow and clarity.

In Paragraph 6, it would be worth noting that the potential of farming turtles in the tropics for meat markets should be further explored as it may represent a potential decrease in pressures on wild populations (Mali et al. 2015)
Mali, I., H.H. Wang, W.E. Grant, M. Feldman, and M.R.J. Forstner. 2015. Modeling commercial freshwater turtle production on US farms for pet and meat markets. PLoSONE 10(9): e0139053. doi: 10.1371/journal.pone.0139053.
Our reply: Included as follows: "Considering these results, harvesting wild adults would appear to present a high risk of causing population declines whether in the temperate or tropical regions, reinforcing the need to develop appropriately enforced alternate management options such as farming of captive reared turtles for meat [89]."

Thank you for addressing these comments.

Reviewer #2: This is an interesting piece of work focused on the potential of sustainable harvest as a conservation measure for turtles, comparing the life history traits and demographic sensitivities of temperate vs. tropical species. Despite similar studies focused on particular species exist, none has previously investigated the harvest potential of freshwater turtles at a global scale. Research objectives are clear, the literature review effort behind is impressive and the manuscript is well

written overall. However, I think the manuscript still needs major improvements for it to be publishable, especially regarding the methodology and presentation of results.

Major concerns:

Methodological coherence: the main aim of this study is to compare demographic parameters of tropical vs temperate freshwater turtle species. This is done when describing life history parameters and when projecting population growth but not when constructing the GLMMs; instead, all data is analysed together. Why not conducting two separate analysis? I suggest to conduct one for tropical species and another for temperate ones and compare results regarding bioclimatic effects on each group. This could help show the differences/similarities between tropical and temperate taxa more clearly and focus the discussion on these comparison.
Our reply: This is explicitly included via including latitudinal zones in the revised analysis.

Variables chosen in GLMMs: the authors choose latitudinal and bioclimatic variables as fixed effects in their models, but the hypotheses behind this decision or their expected effects on life history parameters are not described and should be presented in the introduction or in the methods section.

Our reply: We have extensively revised the Methods to clarify variable choice.

Also, there could be some redundancy as latitude may be a proxy of annual temperature, so you seem to be testing the same hypotheses or effects twice in your analysis. Indeed, results (beta slopes) of the model considering latitude as continuous variable and those of the bioclimatic model have identical interpretation (they both show significant temperature effects on clutch size, clutch frequency and age at sexual maturity). I suggest removing the continuous latitude variable and keep only continuous bioclimatic variables in the analysis.
Our reply: We have now included two bioclimatic variables that are only weakly correlated with latitude. This choice is detailed in the Methods and the updated results presented in Table 2 and Table 3. This enables us to focus the Discussion on the effects of temperature.

Missing information and omitted findings: It seems to me that some relevant information is missing regarding the population projections and the results of the analysis with GLMMs. What was the initial population size chosen in the population projection models? Do you consider a stable age distribution (SAD)? What is the proportion of individuals in each stage of the population (egg, juvenile, adult)?

Our reply: Yes, we use a stable stage distribution. We have added these details to Methods (p l) as follows: "The model consisted of egg, juvenile, and adult stages (Fig 1) projected with a stable stage distribution (initial population of 1000, allocated in proportions of 0.544, 0.401, 0.055 to egg, juvenile and adult stages respectively)."

In the methods section, annual precipitation (bio12) is mentioned as one of the variables considered in the GLMMs, but it never appears in tables 2 and 3 and only results for the temperature variable (bio1) are shown. I could not find any explanation for that in the manuscript, except that precipitation variables were not as good as temperature variables (but no statistical significance values nor beta slopes are given). All models constructed (including those receiving low statistical support) must be presented in the results tables as well as the estimated parameters (beta slopes).
Our reply: The revised version includes new bioclimatic variables bio10 and bio17 and the updated results (including beta slopes) are presented in Table 2 and Table 3.

Minor comments

Line and page numbers are missing in the manuscript, please add them prior to re-submission in order to ease future reviews.

Our reply: we have added line and page numbers.

The manuscript is well written but I have detected some errors in spelling (“meant” instead of “mean”) and in word use (“temperature” when referring to “temperate” species) that indicates correct spelling and redaction should be checked in the manuscript.

Our reply: we have carefully reviewed the text to ensure such typos have been corrected.

Table 2: I suggest to remove this table and present it as supporting information, given that interesting beta values are already given in the text (results section) and the relevant information regarding the GLMMs analysis is provided in table 3.

Our reply: We thank the reviewer for the suggestion. We prefer to retain Table 2 in the main text. We believe this facilitates a clear text for readers as Table 2 is heavily cited in the Results and we therefore prefer to retain so readers do not have to go back and forth to Supplemental Material to examine these important results. But we are happy to follow editorial guidance regarding the need to reorganize the presentation of results in the tables.

Table 3: Model notation should change and be less “R-like”. Readers are more interested in knowing the hypotheses behind each model and less in the R code used, so models should be named in the light of the hypotheses being tested each time (i.e. latitude effect, temperature effect etc.).

Our reply: Following this helpful suggestion we have updated both Table 2 and Table 3 to improve clarity for readers.

Table 3: why only 2 models for Adult survival?

Our reply: As explained in the Methods of the original submission a reduced number of models were tested with adult survival due to the limited sample size. In the revised version we have updated and expanded our literature search and have been able to increase the number of studies with adult survival. This increased sample size has now enabled us to update adult survival with the full model set.

Table 3: this table shows many information criteria for model selection, but only the AIC is defined in the methods section. Alternative criteria such as Log-likelihood or BIC (Bayesian?) are not defined in the methods nor in the table legend but are shown in the table anyway (Why?). I suggest to remove them.

Our reply: We have added selection criteria definitions with citations in the Table 3 footnote to clarify for readers. It is standard (best) practice to include multiple selection criteria. Selection

is via AICc (as explained in the methods) and we add additional criteria so that interested readers can evaluate the results and conclusions in more detail. In our case the multiple criteria show the same genial patterns, which provide additional confidence as to the robustness of the analysis and validity of the conclusions. These are all standard selection criteria and as we do not deal with any evaluation of statistical selection criteria, we therefore feel there is no need to add any further details. But we are happy to follow editorial guidance regarding the need for any additional methodological clarification.

Also, there may be some redundancy between AIC weight and delta AIC as both tend to highlight the best supported model, so one of them could be also removed. Deviance values might be removed too as the AIC is already computed using the deviance.

Our reply: We now retain variables included in a confidence set of models. This approach explicitly uses AIC weight which is obtained by ordering variables by AICc value. We feel including both AIC weight and delta AIC enables readers to fairly evaluate our results and the robustness of the conclusions, but we are happy to follow editorial guidance regarding the need for any additional methodological clarification.

Table 3: for clutch frequency, the bioclimatic model (bio1) is the best one, but the latitude model is also well supported, with almost the same AIC weight (0.45 vs. 0.46). I think this shows that latitudinal and temperature variables are redundant (see major comments). If not, I think this result deserves to be mentioned in the manuscript and probably discussed.

Our reply: Based on this and the previous comments we have updated the bioclimatic variables and the analysis, Results and Discussion. For example we now retain all variables included in a confidence set of models.

Figure 3: It shows the same information as in table 2. I suggest to remove table 2 and upload it as supporting information (see previous comment on table 2)

Our reply: We have updated both Figure 3 and Table 2, and feel there is now no duplication of results. We prefer to retain Table 2 in the main text. We believe this facilitates reading of the text for readers as Table 2 is heavily cited in the Results and we therefore prefer to retain so readers do not have to go back and forth to Supplemental Material to examine these important results. But we are happy to follow editorial guidance regarding the need to reorganize the presentation of results in the tables.

Figure 5: fecundity could be more reduced in tropical than in temperate species without causing negative population growth. This is the clearest result I see here, but it is not mentioned in the results nor discussed in the manuscript. Does not fecundity determine egg production? You should discuss the implications of this finding on the capacity to sustain harvest.

Our reply: We thank the reviewer for the suggestion. It is rather like the case of chicken and egg – the question as to whether turtle species are more fecund because of increased egg production or has increased egg production because it is more fecund remains unanswered. Fecundity is not heavily discussed as it is unlikely to be suitable for any sort of conservation management action. Our focus is on exploring the sustainable harvest of turtle species which

directly targets survival of the different stages. Fecundity is obviously important and included in the projection matrix but is not normally of interest for any management actions. We prefer to retain Discussion text focused on the sustainable harvest of different life stages in tropical and temperate turtles and are happy to follow editorial guidance for the need of any additional content in the Discussion.
