# Supplementary material for: Population dynamics and biological feasibility of sustainable harvesting as a conservation strategy for tropical and temperate freshwater turtles
Source: PLoS One. 2020 Feb 27;15(2):e0229689. doi: 10.1371/journal.pone.0229689 (PMC7046234; doi:10.1371/journal.pone.0229689)
Supplement: Attachment — Submitted filename: Rchmansah_replies.docx [file pone.0229689.s003.docx]

From: **PLOS ONE** <[em@editorialmanager.com](mailto:em@editorialmanager.com)>
Date: Sat, Dec 5, 2015 at 5:08 PM
Subject: PLOS ONE Decision: Revise [PONE-D-15-44735] - [EMID:be9d1b1768505f52]
To: angga rachmansah <[angga.rachmansah@gmail.com](mailto:angga.rachmansah@gmail.com)>

PONE-D-15-44735
Biological Feasibility of Sustainable Harvesting Programs as a Conservation Strategy for Tropical Freshwater Turtles
PLOS ONE

Dear Mr rachmansah,

Thank you for submitting your manuscript to PLOS ONE. After careful consideration, we feel that it has merit, but is not suitable for publication as it currently stands. Therefore, my decision is "Major Revision."

We invite you to submit a revised version of the manuscript that addresses the points below:

*** I liked this ms very much and think that after you address the comments of the referees and those of my own it should be ready for publication. However, I must warn you that these changes may be substantial.

Both reviews are excellent and hope that you can make a good use of them. Rev. #1 provides relevant comments to improve your ms and especially points to missing literature that should be cited and Rev. #2 suggests expanding and changing the statistical analyses and has main concerns on the modelling which should be addressed before publication is granted. This reviewer suggests to look at explanatory patterns within each latitude (basically what it could be summarized as looking for a predictor*latitude interaction on life history traits). He/she also asks for you to state your predictions/expectations beforehand in your Intro and Methods section.

I found that the Discussion is the weakest part of the ms, particularly because you mostly address the turtle literature but do not frame it in the extensive literature on life history traits. I provide some hints to help improving the Discussion and finding support to your findings from what we know across organisms and from theoretical standpoints.

Unfortunately your ms did not have line nor page numbers. In order to comment on it I therefore copied the actual text that I am referring to without reference to any page or line numbers.

What is “A” in the model?
**Our reply: We have clarified the text (p l) as follows “The discrete stage based lifecycle (Fig 1) can be presented as a population projection matrix “A” as follows:”**

A sensitivity analysis changing the 0.2 egg survival rate value would be advisable, as we know that usually interactions are stronger in the tropics (literature by Dobzhansky, Schemske, summarized in Bekmam 2013 Ecol. Lett. 16:1054-1060) and this value could actually change with latitude. The same could apply to other sources of (adult and juvenile) mortality, as in principle predation and parasitism (sources of mortality) should be higher in the tropics.
**Our reply: We include results from the sensitivity analysis in Figure 5. We have now included estimate of variation around this 0.2 value in our sensitivity analysis (standard deviation from Iverson 1991). The egg survival value is likely to vary but the most robust synthesis available suggest that 0.2 is a suitable value for freshwater species (Iverson 1991). Our estimates of juvenile mortality are expressed as a percentage of adult mortality. These values and their range (from species level data) are included in the sensitivity analysis. There are therefore differences in juvenile survival values between temperate and tropical zone species (see updated Table 4 and Figure 5). We have updated and clarified the Methods to reflect this. We are not aware of any studies that present empirical data necessary to robustly inform a more in-depth analysis. We hope that future studies can further refine these analyses.**

**Gibbs JP, Amato GD. Genetics and Demography in Turtle Conservation. In: Klemens MW, editor. Turtle Conservation. Washington, DC: The Smithsonian Institution; 2000.**

**Iverson JB. Patterns of survivorship in turtles (order Testudines). Canadian Journal of Zoology. 1991;69:385 - 91.**

In particular, the above could also respond to this paragraph: “An important caveat is that the population dynamics of temperate and tropical species in this study were evaluated using the same survival rate values for egg and juvenile stages due to lack of available published data on these parameters both in temperate and tropical species. Mechanisms of protection of egg and juvenile stages do not produce as large an effect on population growth as to protections adult survival [24], so our conclusions may remain valid despite this unassessed assumption. That said, until data are available on typical nest and juvenile survival in temperate and tropical zones, the relative impact of harvest on populations of temperate and tropical species we estimated must remain tentative.”
**Our reply: We share data and code to encourage further studies to address the broad range of questions that can be asked with the data we have compiled and shared. We believe that until data are available on typical nest and juvenile survival in temperate and tropical zones, the relative impact of harvest on populations of temperate and tropical species we estimated must remain tentative.**

The reported values do not match the text, please check: “whereas natural logarithm of clutch frequency (β = -0.04; P < 0.001) was positively related to annual mean temperature (Fig 4).”
**Our reply: Based on the helpful suggestions from both the editor and reviewers we have extensively updated the analysis and results. The text has been revised to ensure consistency of the reported values.**

“confidence intervals” in regression fits (where the spread of the confidence region changes with X) should be “confidence bands”. Please, change accordingly.
**Our reply: We have updated the text accordingly.**

Discussion
Please provide R2 values of your lmer models (see Nakagawa 2013 Meth Ecol Evol and library MuMIn in R) to support this sentence in the Discussion: “The results of this study revealed that turtle life history is strongly related to latitude and ambient temperature.”

**Our reply: Based on the helpful suggestions from the editor and reviewers we have extensively updated the analysis and results. We have included r2 values in the revised version (Table 3).**

A low p-value or a regression coefficient without standardization is not enough to support your claims. A large proportion of the variance may be still left to be explained.
**Our reply: Based on the helpful suggestions from the editor and reviewers we have extensively updated the analysis and results. We have also revised the text to ensure conclusions and results are supported by the data and analysis presented.**

“Iverson et al. [32] concluded that higher juvenile competition due to shorter time period for development along with higher egg mortality associated with winter and climate uncertainty that creates temporary periods of low competition may make it more advantageous for temperate turtle species to produce more offspring as a “bet hedging” strategy to exploit temporary resources.” This is too speculative, especially since you use a 0.2 egg survival rate from one single place because there are not comparative data available. Is there evidence that buried eggs suffer mortality from cold temperatures?

**Our reply: We have now added the reference of Iverson 1991 [1] to the sentence. This is one of the classic turtle demography references, including a pioneering and meticulous compilation of 81 age-class-specific survivorship values representing 30 turtle species. It is not a “single place”. Iverson [1] provides a combined early stage (egg – to 1 year) annual survival estimate of 0.215+0.188 for freshwater turtles (includes eggs, hatchlings, 1 year that were combined due to “lack of significant differences in survivorship across these three age-classes”). Egg mortality is affected by myriad factors (see supporting references below). But for freshwater turtle species survival estimates of all these early stages are firmly anchored around 0.2 and the SD value (see above) also suggests a consistently low survival value for early stages. In addition for long lived species with multiple reproductive events and in the case of turtles multiple egg laying events, egg survival has consistently been demonstrated to be far less important than survival in juvenile and adult stages [2,3]. Buried eggs can suffer mortality from extreme temperatures (too hot and too cold) and desiccation in both temperate and tropical zones. Turtles have a number of behavioral (e.g. nest depth, substrate choice) and physiological (egg shell thickness, size and shape) adaptations that can help reduce egg mortality (see examples in supporting references below). Such factors are species and location specific. However, we feel that such factors do not affect our conclusions and are not highly relevant to our Discussion. For example, our main finding is that adult harvest is extremely risky (likely to be unsustainable) in both tropical and temperate turtle species. This conclusion is based on an unprecedented compilation of species level demographic data and robust modeling of population dynamics (including sensitivity analysis). For all these reasons we prefer to retain the use of 0.2 egg survival and hope future studies can further refine these analyses based on the data and code we share.**

**[1] Iverson JB. Patterns of survivorship in turtles (order Testudines). Canadian Journal of Zoology. 1991 Feb 1;69(2):385-91.**

**[2]Gibbs JP, Amato GD. Genetics and Demography in Turtle Conservation. In: Klemens MW, editor. Turtle Conservation. Washington, DC: The Smithsonian Institution; 2000.**

**[3]** **Heppell SS. Application of Life - History Theory and Population Model Analysis to Turtle Conservation. Copeia. 1998;1998(2):367 - 75.**

**Supporting references relevant for egg survivorship.**

**Iverson JB, Ewert MA. Physical characteristics of reptilian eggs and a comparison with avian eggs. Egg Incubation: Its Effect on Embryonic Development in Birds and Reptiles. Cambridge University Press, New York, New York, USA. 1991 Dec 5:87-100.**

**Mitchell NJ, Rodriguez N, Kuchling G, Arnall SG, Kearney MR. Reptile embryos and climate change: modelling limits of viability to inform translocation decisions. Biological Conservation. 2016 Dec 1;204:134-47.**

**Packard GC, Tracy CR, Roth JJ. The physiological ecology of reptilian eggs and embryos and the evolution of viviparity within the Class Reptilia. Biological Reviews. 1977 Feb;52(1):71-105.**

**Packard MJ, DeMarco VG. Eggshell structure and formation in eggs of oviparous reptiles. Egg Incubation: Its Effect on Embryonic Development in Birds and Reptiles. Cambridge University Press, New York, New York, USA. 1991 Dec 5:53-69.**

**Packard GC. Water relations of chelonian eggs and embryos: is wetter better? American Zoologist. 1999 Apr 1;39(2):289-303.**

Furthermore, “bet hedging” could equally be important in abiotic (temperate) driven environments as in biotic (interactions) driven environments. Actually, a biotic environment could be more variable and unpredictable (see Moya-Laraño 2010 Open Ecol. J. 3:1-10). More eggs with latitude go indeed against a pattern for higher mortality in the tropics. However, this could merely reflect that selection is targeting egg size in the tropics (larger eggs could help offspring escaping stronger predation pressure in the tropics) and that a fundamental egg-size/egg-number trade-off makes then fewer eggs to be laid in the tropics (see Verdeny-Vilalta et al. 2015 J. Evol. Biol. 28:1225-1233 for a similar pattern). Of course, you the explain later in the text that the shell may make a big difference for predation. Is that the case also for turtle-lings? I see you refer to this issue later on in the text and indeed adults are better protected, thus.

This hypothesis seems very plausible according to what we know. But please, do explicitly refer to the egg-size/egg-number trade-off explicitly, not just implicitly as you do here. “In addition, Moll and Moll [12] concluded that temperate turtle species typically have small egg size to speed development as an adaptation to short incubation times in temperate zone [32]. As such, temperature zone turtles may have evolved to produce smaller egg size with larger clutch size
than tropical turtle species.”

**Our reply: We have included egg-size/egg-number trade off specifically as follows “Additionally, clutch mass (number of eggs x egg size) can also vary with latitude[33, 41], further studies are necessary to examine how egg size correlates to differences in population growth rates, especially as egg size has been shown to be an important predictor of age at sexual maturity [32, 33].”…….**

“The interaction between age at sexual maturity and latitude observed in this study is also in agreement with the earlier studies [62-64].”
please change it with
“The relationship between age at sexual maturity and latitude observed in this study is also in agreement with the earlier studies [62-64].”
you are not testing statistical interactions, which mean rather the opposite (multiplicative vs additive effects).
**Our reply: We have made the correction.**

The following sentence is fundamentally incomplete:
“Large body size provides advantages for temperate turtle species to cope with unfavorable environment through increasing their fitness [33, 66]. As a result, temperate turtle species require longer time to reach size at sexual maturity.”
**Our reply: We have rephrased as follows to provide a link to the following paragraph which deals with adult survival: “As a result, temperate turtle species require longer time to reach size at sexual maturity, but increased size may provide for increased adult survivorship [32]. ”**

Even though larger body sizes increase cold hardiness, to reach this body size turtles will have to face with longer periods of time (several seasons!), which increases the probability of death before reproduction and, in addition, they will have to go through all those body size stages that are more dangerous (in which they are more vulnerable). You are implicitly referring to Bergmann’s and inverse Bergmann’s rule here. Please, do check a bit the extensive recent body of literature on this issue and discuss the explanations researchers have offered for what it may be occurring in ectotherms. A temperature constraint to reach maturation in a single season may be more appropriate as an explanation for the increase in large body size with latitude, I think. furthermore, this part “species to cope with unfavorable environment through increasing their fitness” is a circular argument. You mean that body size is larger because it provided a fitness advantage in the (evolutionary) past? increasing the fitness to cope with the unfavorable environment is biologically non-sense as fitness is an (evolutionary meaningful) response. Please change accordingly.
**Our reply: We have extensively revised this paragraph on page 23 (L) to clarify the text following the Editors suggestions.**

For more information on how to upload your revised submission, see our video: <http://blogs.plos.org/everyone/2011/05/10/how-to-submit-your-revised-manuscript/>

If you choose not to submit a revision, please notify us.

Yours sincerely,

Jordi Moya-Larano
Academic Editor
PLOS ONE


Journal requirements:

When submitting your revision, we need you to address these additional requirements.

Please ensure that your manuscript meets PLOS ONE's style requirements, including those for file naming. The PLOS ONE style templates can be found at <http://www.plosone.org/attachments/PLOSOne_formatting_sample_main_body.pdf> and <http://www.plosone.org/attachments/PLOSOne_formatting_sample_title_authors_affiliations.pdf>

** It was noted by our internal staff that Figure 2 in your manuscript have been previously copyrighted.
**Our reply: The revised version has figures that are fully compliant with plosone copyright guidelines. We clarify this in the figure legend as follows: “Fig 2. Distribution of freshwater turtle studies. Geographic distribution of data on freshwater turtle life history traits obtained from the literature ( S1 Table) to estimate capacity for sustainable harvest in freshwater turtles. Color of study locations represent the latitudinal distribution of the study species. Dashed horizontal lines show Tropic of Cancer and Tropic of Capricorn. The background map was obtained from the 1:110m Natural Earth country and geographic lines maps (http://www.naturalearthdata.com).”**

Should your paper be accepted, all images will published under PLOS’ CC BY 4.0 license, which means that they will be freely available online, and any third party is permitted to access, download, copy, distribute, and use these materials in any way, even commercially, with proper attribution. For more information, see our Figure guidelines: <http://www.plosone.org/static/figureGuidelines#policies>

With regards to the previously copyrighted figures in your submission, we require you to either present written permission from the copyright holder to publish these figures, or remove the figures from your submission.
**Our reply: The revised version has figures that are fully compliant with plosone copyright guidelines. We clarify this in the figure legend as follows: “Fig 2. Distribution of freshwater turtle studies. Geographic distribution of data on freshwater turtle life history traits obtained from the literature ( S1 Table) to estimate capacity for sustainable harvest in freshwater turtles. Color of study locations represent the latitudinal distribution of the study species. Dashed horizontal lines show Tropic of Cancer and Tropic of Capricorn. The background map was obtained from the 1:110m Natural Earth country and geographic lines maps (http://www.naturalearthdata.com).”**

1. To seek permission from the original copyright holder of Figure 2 to publish it under the specific Creative Commons Attribution License (CCAL), CC BY 4.0, we recommend that you contact the original copyright holder with the following text:

“I request permission for the open-access journal PLOS ONE to publish XXX under the Creative Commons Attribution License (CCAL) CC BY 4.0 (<http://creativecommons.org/licenses/by/4.0/>). Please be aware that this license allows unrestricted use and distribution, even commercially, by third parties. Please reply and provide explicit written permission to publish XXX under a CC BY license.”

Please upload the granted permission to the manuscript as a supporting information file. In the figure caption of the copyrighted figure, please include the following text: “Reprinted from [ref] under a CC BY license, with permission from [name of publisher], original copyright [original copyright year].”

2. If you are unable to obtain permission from the original copyright holder, please either i) remove the figure or ii) supply a replacement figure that complies with the CC BY 4.0 license, which can be for illustrative purposes only. Please check copyright information on all replacement figures and update the figure caption with source information. If applicable, please specify in the figure caption text when a figure is similar but not identical to the original image, and is therefore for illustrative purposes only.

3. It was noted that the figure(s) in question contains a map or satellite image. PLOS ONE is unable to publish previously copyrighted maps or satellite images, or images created using proprietary data. For these reasons, we cannot publish images generated by Google software (Google Maps, Street View, and Earth). If the content of the manuscript depends on the use of Google software, you may need to provide replacement images that are representative of the Google-generated images. The following resources for replacing copyrighted map figures may be helpful:

OpenStreetMap (data, but not generated images, are open): <http://www.openstreetmap.org/>

USGS National Map Viewer (public domain): <http://viewer.nationalmap.gov/viewer/>

The Gateway to Astronaut Photography of Earth (public domain): <http://eol.jsc.nasa.gov/sseop/clickmap/>

Maps at the CIA (public domain): <https://www.cia.gov/library/publications/the-world-factbook/index.html> and <https://www.cia.gov/library/publications/cia-maps-publications/index.html>

NASA Earth Observatory (public domain): <http://earthobservatory.nasa.gov/>

USGS EROS (Earth Resources Observatory and Science (EROS) Center) (public domain): [http://eros.usgs.gov/#](http://eros.usgs.gov/)

Grass GIS (geographic information system) analysis software (data, but not generated images, are open): <http://grass.osgeo.org/>

Grass GIS (geographic information system) analysis software (data, but not generated images, are open): <http://grass.osgeo.org/>
**Our reply: The revised version has figures that are fully compliant with plosone copyright guidelines. We clarify this in the figure legend as follows: “Fig 2. Distribution of freshwater turtle studies. Geographic distribution of data on freshwater turtle life history traits obtained from the literature ( S1 Table) to estimate capacity for sustainable harvest in freshwater turtles. Color of study locations represent the latitudinal distribution of the study species. Dashed horizontal lines show Tropic of Cancer and Tropic of Capricorn. The background map was obtained from the 1:110m Natural Earth country and geographic lines maps (http://www.naturalearthdata.com).”**

Reviewers' comments:

Reviewer's Responses to Questions

**Comments to the Author**

1. Is the manuscript technically sound, and do the data support the conclusions?

The manuscript must describe a technically sound piece of scientific research with data that supports the conclusions. Experiments must have been conducted rigorously, with appropriate controls, replication, and sample sizes. The conclusions must be drawn appropriately based on the data presented.

Reviewer #1: Yes

Reviewer #2: Yes

2. Has the statistical analysis been performed appropriately and rigorously?

Reviewer #1: Yes

Reviewer #2: No

3. Does the manuscript adhere to the PLOS Data Policy?

Authors must follow the [PLOS Data policy](http://journals.plos.org/plosone/s/data-availability), which requires authors to make all data underlying the findings described in their manuscript fully available without restriction. Please refer to the author’s Data Availability Statement in the manuscript. All data and related metadata must be deposited in an appropriate public repository, unless already provided as part of the submitted article or supporting information. If there are restrictions on the ability of authors to publicly share data—e.g. privacy or use of data from a third party—these reasons must be specified.

Reviewer #1: Yes

Reviewer #2: Yes

4. Is the manuscript presented in an intelligible fashion and written in standard English?

PLOS ONE does not copyedit accepted manuscripts, so the language in submitted articles must be clear, correct, and unambiguous. Any typographical or grammatical errors should be corrected at revision, so please note any specific errors here.

Reviewer #1: Yes

Reviewer #2: Yes

5. Review Comments to the Author

Please use the space provided to explain your answers to the questions above. You may also include additional comments for the author, including concerns about dual publication, research ethics, or publication ethics. (Please upload your review as an attachment if it exceeds 20,000 characters)

Reviewer #1: This is a well written and appropriately analyzed manuscript about freshwater turtle sustainability. I have several minor comments that are worth mentioning in the manuscript:
Although extensive amount of literature has been cited that shows overall understanding of turtle natural history, there are several articles that have been recently published and that are relevant to this article:
1. In the introduction in the second paragraph, the authors should mention that although the greatest pressure on turtle harvest occurs in tropical areas, these high pressures can lead to regional population collapse and as a consequence create pressures in other regions of the world (Mali et al. 2014)
Mali, I., M.W. Vandewege, S.K. Davis, and M.R.J. Forstner. 2014. Magnitude of the freshwater turtle exports from the US: long term trends and early effects of newly implemented harvest management regimes. PLoSONE 9(1): e86478. doi:10.1371/journal.pone.0086478.
**Our reply: Based on the helpful comments from the Editor and reviewers we have extensively revised and updated the literature cited. We have included in the revised text as follows “Additionally, unsustainable exploitation in tropical areas can also lead to regional population collapse and as a consequence create pressures in other regions of the world [24].”**

2. Discussion paragraph 6- In talking about risks of harvesting adults and juveniles, the study of Zimmer-Shaffer et al. 2014 should also be mentioned.
Zimmer-Shaffer, S.A., J.T. Briggler, and J.J. Millspaugh. 2014. Modeling the effects of commercial harvest on population growth of river turtles. Chelonian Conservation and Biology 13(2):227-237.
**Our reply: We have added as follows “In addition, similar to previous studies [29, 30, 34, 74, 85-88], high adult and juvenile survival rates are estimated to be critical to maintain population stability due to their relatively greater contribution to population recruitment than other life stages [34].”**

Additional minor comments:
Citation [45] is missing in the body of the article. Please address

**Our reply: Based on the helpful comments from the Editor and reviewers we have extensively revised and updated the literature cited. We believe references and citations are consistent.**

Abstract- "Turtles at low latitudes (tropical zones) exhibit similar adult survival rates...."
Delete "similar adult survival rates"
**Our reply: Corrected as suggested.**

Introduction, Paragraph 2 and 3- Em dash was used for "15-18" and "25-27" citation and in the rest of the article hyphen was used.
**Our reply: We have carefully revised all formatting in the text and references.**

Introduction, Paragraph 3- Last citation could be fixed from [24,29-29] to [24,29]
**Our reply: We have carefully revised all formatting in the text and references.**

Results, Paragraph 1- In the last sentence replace "Just" with "Only" and ";" with "while"
**Our reply: Corrected as suggested.**

Discussion, Paragraph 5- delete "only" in the sentence "It is important to note....."
**Our reply: Corrected as suggested. We have extensively revised the Discussion to improve flow and clarity.**

Discussion, Paragraph 6- Correct the sentence "Egg harvest is may be....." to "Egg harvest may be more feasible..."
**Our reply: Corrected as suggested. We have extensively revised the Discussion to improve flow and clarity.**

In Paragraph 6, it would be worth noting that the potential of farming turtles in the tropics for meat markets should be further explored as it may represent a potential decrease in pressures on wild populations (Mali et al. 2015)
Mali, I., H.H. Wang, W.E. Grant, M. Feldman, and M.R.J. Forstner. 2015. Modeling commercial freshwater turtle production on US farms for pet and meat markets. PLoSONE 10(9): e0139053. doi: 10.1371/journal.pone.0139053.
**Our reply: Included as follows: “Considering these results, harvesting wild adults would appear to present a high risk of causing population declines whether in the temperate or tropical regions, reinforcing the need to develop appropriately enforced alternate management options such as farming of captive reared turtles for meat [89].”**

Thank you for addressing these comments.

Reviewer #2: This is an interesting piece of work focused on the potential of sustainable harvest as a conservation measure for turtles, comparing the life history traits and demographic sensitivities of temperate vs. tropical species. Despite similar studies focused on particular species exist, none has previously investigated the harvest potential of freshwater turtles at a global scale. Research objectives are clear, the literature review effort behind is impressive and the manuscript is well written overall. However, I think the manuscript still needs major improvements for it to be publishable, especially regarding the methodology and presentation of results.

Major concerns:

Methodological coherence: the main aim of this study is to compare demographic parameters of tropical vs temperate freshwater turtle species. This is done when describing life history parameters and when projecting population growth but not when constructing the GLMMs; instead, all data is analysed together. Why not conducting two separate analysis? I suggest to conduct one for tropical species and another for temperate ones and compare results regarding bioclimatic effects on each group. This could help show the differences/similarities between tropical and temperate taxa more clearly and focus the discussion on these comparison.
**Our reply: This is explicitly included via including latitudinal zones in the revised analysis.**

Variables chosen in GLMMs: the authors choose latitudinal and bioclimatic variables as fixed effects in their models, but the hypotheses behind this decision or their expected effects on life history parameters are not described and should be presented in the introduction or in the methods section.

**Our reply: We have extensively revised the Methods to clarify variable choice.**

Also, there could be some redundancy as latitude may be a proxy of annual temperature, so you seem to be testing the same hypotheses or effects twice in your analysis. Indeed, results (beta slopes) of the model considering latitude as continuous variable and those of the bioclimatic model have identical interpretation (they both show significant temperature effects on clutch size, clutch frequency and age at sexual maturity). I suggest removing the continuous latitude variable and keep only continuous bioclimatic variables in the analysis.
**Our reply: We have now included two bioclimatic variables that are only weakly correlated with latitude. This choice is detailed in the Methods and the updated results presented in Table 2 and Table 3. This enables us to focus the Discussion on the effects of temperature.**

Missing information and omitted findings: It seems to me that some relevant information is missing regarding the population projections and the results of the analysis with GLMMs. What was the initial population size chosen in the population projection models? Do you consider a stable age distribution (SAD)? What is the proportion of individuals in each stage of the population (egg, juvenile, adult)?

**Our reply: Yes, we use a stable stage distribution. We have added these details to Methods (p l) as follows: “The model consisted of egg, juvenile, and adult stages (Fig 1) projected with a stable stage distribution (initial population of 1000, allocated in proportions of 0.544, 0.401, 0.055 to egg, juvenile and adult stages respectively).”**

In the methods section, annual precipitation (bio12) is mentioned as one of the variables considered in the GLMMs, but it never appears in tables 2 and 3 and only results for the temperature variable (bio1) are shown. I could not find any explanation for that in the manuscript, except that precipitation variables were not as good as temperature variables (but no statistical significance values nor beta slopes are given). All models constructed (including those receiving low statistical support) must be presented in the results tables as well as the estimated parameters (beta slopes).
**Our reply: The revised version includes new bioclimatic variables bio10 and bio17 and the updated results (including beta slopes) are presented in Table 2 and Table 3.**

Minor comments

Line and page numbers are missing in the manuscript, please add them prior to re-submission in order to ease future reviews.

**Our reply: we have added line and page numbers.**

The manuscript is well written but I have detected some errors in spelling (“meant” instead of “mean”) and in word use (“temperature” when referring to “temperate” species) that indicates correct spelling and redaction should be checked in the manuscript.

**Our reply: we have carefully reviewed the text to ensure such typos have been corrected.**

Table 2: I suggest to remove this table and present it as supporting information, given that interesting beta values are already given in the text (results section) and the relevant information regarding the GLMMs analysis is provided in table 3.

**Our reply: We thank the reviewer for the suggestion. We prefer to retain Table 2 in the main text. We believe this facilitates a clear text for readers as Table 2 is heavily cited in the Results and we therefore prefer to retain so readers do not have to go back and forth to Supplemental Material to examine these important results. But we are happy to follow editorial guidance regarding the need to reorganize the presentation of results in the tables.**

Table 3: Model notation should change and be less “R-like”. Readers are more interested in knowing the hypotheses behind each model and less in the R code used, so models should be named in the light of the hypotheses being tested each time (i.e. latitude effect, temperature effect etc.).

**Our reply: Following this helpful suggestion we have updated both Table 2 and Table 3 to improve clarity for readers.**

Table 3: why only 2 models for Adult survival?

**Our reply: As explained in the Methods of the original submission a reduced number of models were tested with adult survival due to the limited sample size. In the revised version we have updated and expanded our literature search and have been able to increase the number of studies with adult survival. This increased sample size has now enabled us to update adult survival with the full model set.**

Table 3: this table shows many information criteria for model selection, but only the AIC is defined in the methods section. Alternative criteria such as Log-likelihood or BIC (Bayesian?) are not defined in the methods nor in the table legend but are shown in the table anyway (Why?). I suggest to remove them.

**Our reply: We have added selection criteria definitions with citations in the Table 3 footnote to clarify for readers. It is standard (best) practice to include multiple selection criteria. Selection is via AICc (as explained in the methods) and we add additional criteria so that interested readers can evaluate the results and conclusions in more detail. In our case the multiple criteria show the same genial patterns, which provide additional confidence as to the robustness of the analysis and validity of the conclusions. These are all standard selection criteria and as we do not deal with any evaluation of statistical selection criteria, we therefore feel there is no need to add any further details. But we are happy to follow editorial guidance regarding the need for any additional methodological clarification.**

Also, there may be some redundancy between AIC weight and delta AIC as both tend to highlight the best supported model, so one of them could be also removed. Deviance values might be removed too as the AIC is already computed using the deviance.

**Our reply: We now retain variables included in a confidence set of models. This approach explicitly uses AIC weight which is obtained by ordering variables by AICc value. We feel including both AIC weight and delta AIC enables readers to fairly evaluate our results and the robustness of the conclusions, but we are happy to follow editorial guidance regarding the need for any additional methodological clarification.**

Table 3: for clutch frequency, the bioclimatic model (bio1) is the best one, but the latitude model is also well supported, with almost the same AIC weight (0.45 vs. 0.46). I think this shows that latitudinal and temperature variables are redundant (see major comments). If not, I think this result deserves to be mentioned in the manuscript and probably discussed.

**Our reply: Based on this and the previous comments we have updated the bioclimatic variables and the analysis, Results and Discussion. For example we now retain all variables included in a confidence set of models.**

Figure 3: It shows the same information as in table 2. I suggest to remove table 2 and upload it as supporting information (see previous comment on table 2)

**Our reply: We have updated both Figure 3 and Table 2, and feel there is now no duplication of results. We prefer to retain Table 2 in the main text. We believe this facilitates reading of the text for readers as Table 2 is heavily cited in the Results and we therefore prefer to retain so readers do not have to go back and forth to Supplemental Material to examine these important results. But we are happy to follow editorial guidance regarding the need to reorganize the presentation of results in the tables.**

Figure 5: fecundity could be more reduced in tropical than in temperate species without causing negative population growth. This is the clearest result I see here, but it is not mentioned in the results nor discussed in the manuscript. Does not fecundity determine egg production? You should discuss the implications of this finding on the capacity to sustain harvest.

**Our reply: We thank the reviewer for the suggestion. It is rather like the case of chicken and egg – the question as to whether turtle species are more fecund because of increased egg production or has increased egg production because it is more fecund remains unanswered. Fecundity is not heavily discussed as it is unlikely to be suitable for any sort of conservation management action. Our focus is on exploring the sustainable harvest of turtle species which directly targets survival of the different stages. Fecundity is obviously important and included in the projection matrix but is not normally of interest for any management actions. We prefer to retain Discussion text focused on the sustainable harvest of different life stages in tropical and temperate turtles and are happy to follow editorial guidance for the need of any additional content in the Discussion.**
