# Supplementary material for: Population dynamics and biological feasibility of sustainable harvesting as a conservation strategy for tropical and temperate freshwater turtles
Source: PLoS One. 2020 Feb 27;15(2):e0229689. doi: 10.1371/journal.pone.0229689 (PMC7046234; doi:10.1371/journal.pone.0229689)
Supplement: Attachment — Submitted filename: Rachmansah_replies02.docx [file pone.0229689.s007.docx]

PONE-D-19-28843R1
Population Dynamics and Biological Feasibility of Sustainable Harvesting as a Conservation Strategy for Tropical and Temperate Freshwater Turtles
PLOS ONE

Dear Dr Norris,

Thank you for submitting your manuscript to PLOS ONE. After careful consideration, we feel that it has merit but does not fully meet PLOS ONE’s publication criteria as it currently stands. Therefore, we invite you to submit a revised version of the manuscript that addresses the points raised during the review process.

I think you have responded to all of the comments and revised the manuscript accordingly. A reviewer provided comments, and I would like to give you an opportunity to respond to them (and possibly incorporate them). Please also read the formatting and other guideline of PLoS One carefully at this stage and resubmit it.

Reply: We are glad that the Editor and reviewer agree that we have adequately responded to the extensive and detailed suggestions that followed our initial submission. We have revised the content to ensure that we follow PLoSone guideline. Based on the reviewers minor suggestion we have added a further sentence to clarify the text for readers. We have added the phrase “species specific” at line 437 :

And extended the following sentence at line 463 to include the examples suggested by the reviewer: “Further examples are needed to understand how the predicted surplus in early life stages can be most effectively exploited in other tropical species, especially small sized and secretive species (e.g. kinosternids in the Americas or geomydids in southeastern Asia).”

We would appreciate receiving your revised manuscript by Mar 22 2020 11:59PM. When you are ready to submit your revision, log on to <https://www.editorialmanager.com/pone/> and select the 'Submissions Needing Revision' folder to locate your manuscript file.

If you would like to make changes to your financial disclosure, please include your updated statement in your cover letter.

To enhance the reproducibility of your results, we recommend that if applicable you deposit your laboratory protocols in protocols.io, where a protocol can be assigned its own identifier (DOI) such that it can be cited independently in the future. For instructions see: <http://journals.plos.org/plosone/s/submission-guidelines#loc-laboratory-protocols>

Please include the following items when submitting your revised manuscript:

- A rebuttal letter that responds to each point raised by the academic editor and reviewer(s). This letter should be uploaded as separate file and labeled 'Response to Reviewers'.
- A marked-up copy of your manuscript that highlights changes made to the original version. This file should be uploaded as separate file and labeled 'Revised Manuscript with Track Changes'.
- An unmarked version of your revised paper without tracked changes. This file should be uploaded as separate file and labeled 'Manuscript'.

Please note while forming your response, if your article is accepted, you may have the opportunity to make the peer review history publicly available. The record will include editor decision letters (with reviews) and your responses to reviewer comments. If eligible, we will contact you to opt in or out.

We look forward to receiving your revised manuscript.

Kind regards,

Masami Fujiwara, PhD
Academic Editor
PLOS ONE


[Note: HTML markup is below. Please do not edit.]

Reviewers' comments:

Reviewer's Responses to Questions

**Comments to the Author**

1. If the authors have adequately addressed your comments raised in a previous round of review and you feel that this manuscript is now acceptable for publication, you may indicate that here to bypass the “Comments to the Author” section, enter your conflict of interest statement in the “Confidential to Editor” section, and submit your "Accept" recommendation.

Reviewer #1: All comments have been addressed

2. Is the manuscript technically sound, and do the data support the conclusions?

The manuscript must describe a technically sound piece of scientific research with data that supports the conclusions. Experiments must have been conducted rigorously, with appropriate controls, replication, and sample sizes. The conclusions must be drawn appropriately based on the data presented.

Reviewer #1: Yes

3. Has the statistical analysis been performed appropriately and rigorously?

Reviewer #1: Yes

4. Have the authors made all data underlying the findings in their manuscript fully available?

The [PLOS Data policy](http://www.plosone.org/static/policies.action#sharing) requires authors to make all data underlying the findings described in their manuscript fully available without restriction, with rare exception (please refer to the Data Availability Statement in the manuscript PDF file). The data should be provided as part of the manuscript or its supporting information, or deposited to a public repository. For example, in addition to summary statistics, the data points behind means, medians and variance measures should be available. If there are restrictions on publicly sharing data—e.g. participant privacy or use of data from a third party—those must be specified.

Reviewer #1: Yes

5. Is the manuscript presented in an intelligible fashion and written in standard English?

PLOS ONE does not copyedit accepted manuscripts, so the language in submitted articles must be clear, correct, and unambiguous. Any typographical or grammatical errors should be corrected at revision, so please note any specific errors here.

Reviewer #1: Yes

6. Review Comments to the Author

Please use the space provided to explain your answers to the questions above. You may also include additional comments for the author, including concerns about dual publication, research ethics, or publication ethics. (Please upload your review as an attachment if it exceeds 20,000 characters)

Reviewer #1: Authors addressed all prevouis comments. I think this is a very complete and improved version of the manuscript. It is clear that your management suggestions should be consider only for large and gregarious species. The application of your results to secretive and small size/clutch species should be wait for more data on specific study systems such emydids and kinosternids in North America or geomydids in Souteastern Asia.

Reply: We are glad that the Editor and reviewer agree that we have adequately responded to the extensive and detailed suggestions that followed our initial submission. We have revised the content to ensure that we follow PLOSone guideline. Based on the reviewers minor suggestion we have added a further sentence to clarify the text for readers. We have added the phrase “species specific” at line 437 :

And extended the following sentence at line 463 to include the examples suggested by the reviewer: “Further examples are needed to understand how the predicted surplus in early life stages can be most effectively exploited in other tropical species, especially small sized and secretive species (e.g. kinosternids in the Americas or geomydids in southeastern Asia).”
